# The Unusual Homodimer of a Heme‐Copper Terminal Oxidase Allows Itself to Utilize Two Electron Donors

**DOI:** 10.1002/anie.202016785

**Published:** 2021-05-06

**Authors:** Guoliang Zhu, Hui Zeng, Shuangbo Zhang, Jana Juli, Linhua Tai, Danyang Zhang, Xiaoyun Pang, Yan Zhang, Sin Man Lam, Yun Zhu, Guohong Peng, Hartmut Michel, Fei Sun

**Affiliations:** ^1^ National Key Laboratory of Biomacromolecules CAS Center for Excellence in Biomacromolecules Institute of Biophysics Chinese Academy of Sciences 15 Datun Road, Chaoyang District Beijing 100101 China; ^2^ Department of Molecular Membrane Biology Max Planck Institute of Biophysics Max-von Laue-Straβe 3 60438 Frankfurt am Main Germany; ^3^ College of Life Sciences University of Chinese Academy of Sciences Beijing 100049 China; ^4^ Center for Biological Imaging Institute of Biophysics Chinese Academy of Sciences 15 Datun Road, Chaoyang District Beijing 100101 China; ^5^ LipidALL Technologies Company Limited Changzhou 213022 Jiangsu Province China; ^6^ State Key Laboratory of Molecular Developmental Biology Institute of Genetics and Developmental Biology Chinese Academy of Sciences No.1 West Beichen Road, Chaoyang District Beijing 100101 China

**Keywords:** cytochrome *c* oxidase, dimerization, enzyme catalysis, naphthoquinone, protein structures

## Abstract

The heme‐copper oxidase superfamily comprises cytochrome *c* and ubiquinol oxidases. These enzymes catalyze the transfer of electrons from different electron donors onto molecular oxygen. A B‐family cytochrome *c* oxidase from the hyperthermophilic bacterium *Aquifex aeolicus* was discovered previously to be able to use both cytochrome *c* and naphthoquinol as electron donors. Its molecular mechanism as well as the evolutionary significance are yet unknown. Here we solved its 3.4 Å resolution electron cryo‐microscopic structure and discovered a novel dimeric structure mediated by subunit I (CoxA2) that would be essential for naphthoquinol binding and oxidation. The unique structural features in both proton and oxygen pathways suggest an evolutionary adaptation of this oxidase to its hyperthermophilic environment. Our results add a new conceptual understanding of structural variation of cytochrome *c* oxidases in different species.

## Graphical abstract

The 3.4 Å structure of cytochrome *c* oxidase from the hyperthermophilic bacterium *Aquifex aeolicus* (AaC*c*O) has been solved. The molecular mechanism that AaC*c*O uses involves both cytochrome *c* and quinol as electron donors through the native quinol molecules (NQs) bound at the dimeric interface.

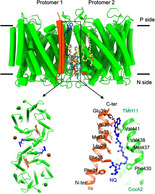

## Introduction

In all respiring organisms electrochemical proton gradients drive the flux of protons back through the membrane via ATP‐synthases, which produces adenosine‐5′‐triphosphate by attaching an inorganic phosphate to adenosine‐5′‐diphosphate. In aerobic organisms, the electrochemical proton gradient is generated by a series of proton translocation reactions in the respiratory chains. Cytochrome *c* oxidase (C*c*O) is the terminal enzyme in the respiratory chains of many aerobic organisms. It is located in the inner membrane of mitochondria and bacteria, and catalyzes the electron transfer from cytochrome *c* to molecular oxygen that is reduced to water. Studies on this integral membrane protein complex revealed that eight protons are taken up from the matrix side of mitochondrial membrane or from the bacterial cytoplasm (N‐side), four protons are pumped across the membrane into the intermembrane space of mitochondria or the periplasm of gram‐negative bacteria (P‐side), while another four protons are used for water formation.^[1]^

C*c*O is a member of the heme‐ and copper‐containing terminal oxidases (HCOs) superfamily,^[2]^ which also includes ubiquinol oxidases (QOXs), for example, the well‐studied cytochrome *bo*_3_ from *Escherichia coli* (*E. coli*)^[3]^ but not the cytochrome *bd* oxidases from the same bacterium.^[4]^ HCOs are classified into three families, A, B and C, based on their amino acid sequences and proton transfer pathways.^[5]^ They are multi‐subunit complexes, for example, they possess 14 protein subunits in mammalian mitochondria^[6]^ and 3 subunits in some bacteria.^[7]^

The conserved central catalytic subunit I contains two heme groups and a copper atom (Cu_B_). The low‐spin heme can be a heme *a* or a heme *b* in prokaryotes,[^3^, ^8^] whereas only heme *a* has been found in mitochondrial cytochrome *c* oxidases.^[9]^ The low‐spin heme *a* in the A‐family C*c*O from *Bos taurus* (BtC*c*O)^[9b]^ and heme *b* in the B‐family C*c*O from *Thermus thermophilus* (TtC*c*O) accept electrons from Cu_A_,^[7]^ and transfer them to the active site that is formed by the high‐spin heme *a*_3_ and Cu_B_. The low‐spin heme *b* in the QOX from *E. coli* directly accepts electrons from ubiquinol and transfers them to the binuclear center that is formed by a high‐spin heme *o*_3_ and Cu_B_.^[10]^ When the binuclear center becomes doubly reduced, dioxygen binds to the heme iron and is reduced to water. The required protons are provided from the cytoplasmic side.

Subunit I of the C*c*Os most often contains 12 transmembrane helices (TMHs). An exception is TtC*c*O whose subunit I possesses 13 transmembrane helices.^[7]^ Subunit II is well conserved in the A‐ and B‐families with its binuclear Cu_A_ center located at the P‐side and accepting electrons from cytochrome *c*,^[11]^ whereas in QOXs subunit II contains two TMHs with Cu_A_ absent.[^10^, ^12^] Subunit III is present in mitochondrial and most bacterial HCOs in A‐family, and could be fused to subunit I.^[13]^

TtC*c*O is the best studied HCO of the B‐family. Its crystal structures in the oxidized state have been reported at resolutions of 2.4 Å^[7]^ and 1.8 Å,^[14]^ respectively. Its proton pathway was found to be similar to the K‐pathway of A‐family C*c*O.^[15]^ Differently, compared to the A‐family C*c*O (PdC*c*O) from *Paracoccus denitrificans* (*P. denitrificans*), subunit II of TtC*c*O only contains one TMH. The position of the second N‐terminal TMH of PdC*c*O subunit II is occupied by the additional subunit IIa of TtC*c*O in an opposite orientation.^[7]^ Although it has been challenged recently,^[16]^ several studies suggested the efficiency of proton pumping in B‐family C*c*Os (H^+^/e^−^=0.5)^[17]^ appears to be lower than that of A‐famliy C*c*Os (H^+^/e^−^=1).^[18]^

*Aquifex aeolicus* (*A. aeolicus*) is a hyperthermophilic chemolithoautotrophic bacterium. The cytochrome *c* oxidase from *A. aeolicus*, AaC*c*O, was previously discovered belonging to B‐family HCO, and interestingly could use both cytochrome *c* and ubiquinol as electron donors,^[19]^ which is a unique feature as a member of B‐family HCO. We originally hypothesized it would be caused by formation of supercomplex between AaC*c*O and complex III, providing additional quinol binding sites to enable its direct oxidation bypass cytochrome *c*. However, by solving the structure of *A. aeolicus* complex III,^[20]^ we did not find novel structural features to support this hypothesis. In addition, our previous study showed the ubiquinol oxidation activity of the potential supercomplex was insensitive to stigmatellin, the inhibitor of ubiquinol binding of complex III.^[19]^ Thus, the ubiquinol oxidation activity of the specimen would most likely come from AaC*c*O itself. It would be the own structural variation of AaC*c*O to gain the function of additional quinol oxidation.

To gain insights into the molecular mechanism of AaC*c*O and also to understand how AaC*c*O adapts its structure to keep stability and activity under hyperthermophilic growth conditions, we purified the AaC*c*O from native membranes and determined its structure at 3.4 Å resolution by using single‐particle electron cryo‐microscopy (cryo‐EM). We found a dimeric form of AaC*c*O with a novel binding site of the native quinol (VII‐tetrahydromultiprenyl‐1,4 naphthoquinone, NQ) at the dimeric interface, which could allow NQH_2_ to be a direct electron donor bypassing cytochrome *c*. Further structure comparisons revealed structural variations of AaC*c*O to increase structural stability and alter the proton transfer pathway as well as the oxygen diffusion pathway for its adaptation of the hyperthermophilic growth environment.

## Results

### Overall structure of AaC*c*O dimer

**Figure 1:**
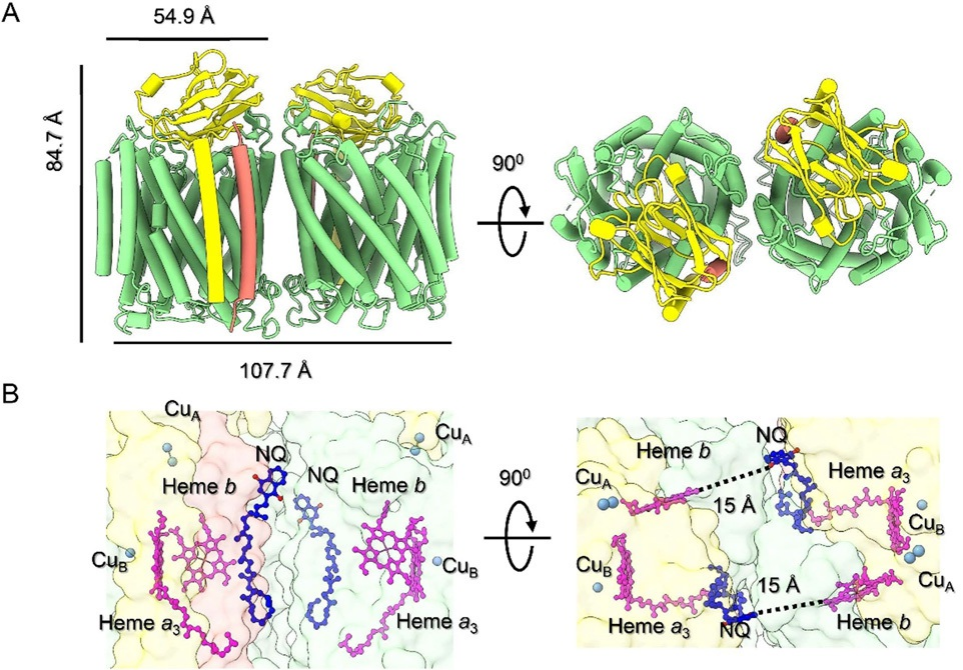
Overall structure of AaC*c*O. A) The protein structure of AaC*c*O is shown in two different views, with the dimensions indicated. The subunits CoxA2, CoxB2, and IIa are colored in green, yellow, and orange, respectively. B) The cofactors and quinols (NQs) are shown as stick representations. The color scheme of subunits CoxA2, CoxB2, and IIa is the same as in (A). The edge‐to‐edge distances between NQ from one protomer and heme *b* from the other protomer are labeled.

The AaC*c*O sample was enriched by anion exchange chromatography and further purified by size‐exclusion chromatography, and the fractions showing a dominant homogenous band around 242 kDa in Blue Native PAGE were used for subsequent cryo‐EM experiments (Figure S1). Based on cryo‐EM 3D classification using RELION^[21]^ (Figure S2), we found two well‐aligned classes of particles existing in the current purified sample. The first class represents the structure of dimeric complex III reported by us before^[20]^ and the second represents the structure of dimeric AaC*c*O. Our substantial image processing does not suggest the existing of any potential supercomplex in this sample. After in silico purification, the dimeric AaC*c*O structure was determined at a final resolution of 3.4 Å according to the gold standard FSC_0.143_ (Fourier Shell Correlation) criterion (Figures S2, S4 and S5; Movie S1). The AaC*c*O dimer exhibits a C2 symmetry and contains three subunits (Figure 1 A), subunit I (CoxA2, 63.9 kDa) with the heme *b* and the heme *a*_3_/Cu_B_ active site, subunit II (CoxB2, 16.8 kDa) with the Cu_A_ center, and subunit IIa (5.2 kDa) (Figure S1). It has dimensions of 84.7 Å in height and 107.7 Å in length. The length of the AaC*c*O monomer is 55.0 Å. The cofactors Cu_A_, Cu_B_, heme *a*_3_ and heme *b* are well resolved (Figure 1 B and Figure S4) with the edge‐to‐edge distance between Cu_A_ and heme *b* Fe 15.4 Å, and the edge‐to‐edge distances from heme *b* to heme *a*_3_ and from heme *a*_3_ to Cu_B_ are 5.0 Å and 5.1 Å, respectively. The overall structure of AaC*c*O is similar to that of TtC*c*O with a root mean square deviation (RMSD) of 1.02 Å for the aligned C_α_ atoms.

### Subunit I: CoxA2

**Figure 2:**
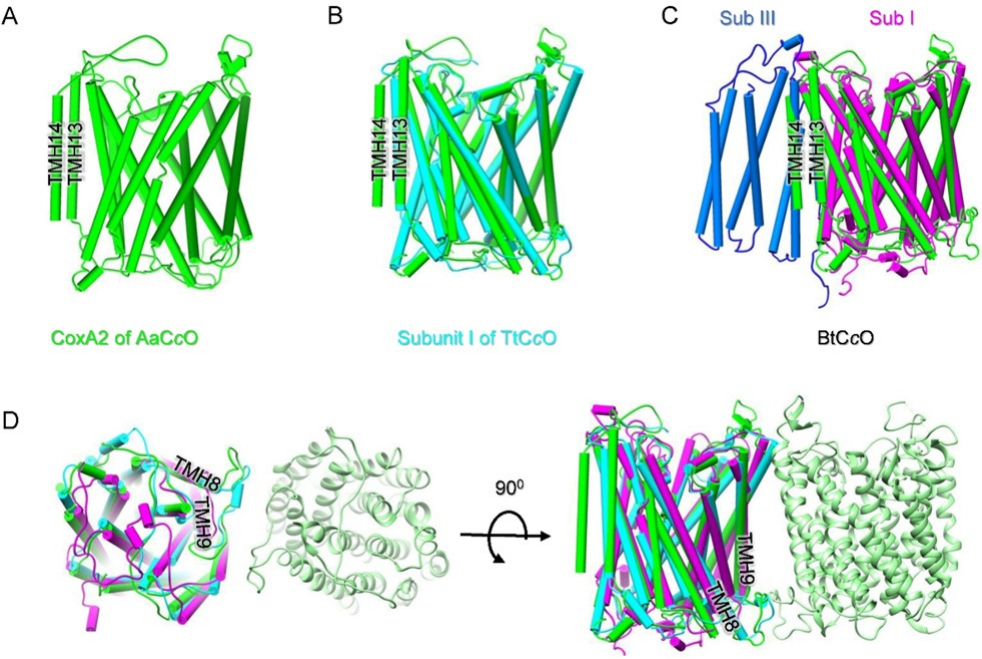
Structure of subunit I (CoxA2). A) The CoxA2 subunit of AaC*c*O possesses 14 TMHs with the C‐terminal TMHs (TMH13 and TMH14) labeled. B) Superposition between CoxA2 subunit of AaC*c*O (green) and subunit I of TtC*c*O (cyan). C) Superposition between CoxA2 subunit of AaC*c*O (green) and subunit I (Sub I) of BtC*c*O (purple). The CoxIII subunit (Sub III) of BtC*c*O is colored in light blue. D) The unique loop between TMH8 and TMH9 in AaC*c*O contributes to the formation of the dimeric structure. Superimposed subunit Is of AaC*c*O, TtC*c*O, and BtC*c*O are colored with the same scheme in (A), (B), and (C).

Surprisingly, subunit I (CoxA2) contains 14 TMHs, two more than the canonical structures of C*c*Os. The two additional TMHs of CoxA2 are found at the C‐terminus (Figure 2 A). This observation is consistent with sequence alignments which show subunit I in prokaryotes has a longer‐C‐terminus than that in eukaryotes (Figure S3). The extra two TMHs bind to the outer surface of TMH5, TMH6, TMH7, and TMH8 of CoxA2. A superimposition of the AaC*c*O and TtC*c*O structures shows that the location of the 13^th^ TMH is same in both complexes (Figure 2 B). The structural superimposition also shows that the additional TMH14 occupies nearly the same site of one TMH of subunit III of the *aa*_3_‐type C*c*O (Figure 2 C). The loops connecting the TMHs of CoxA2 are relatively short and this observation is in accordance with the typical properties of thermostable proteins.^[22]^ Interestingly, the loop between TMH8 and TMH9 at the cytoplasmic surface is longer than that of BtC*c*O. This loop points to the dimer interface (Figure 2 D).

### Subunit II: CoxB2 and Subunit III: IIa

**Figure 3:**
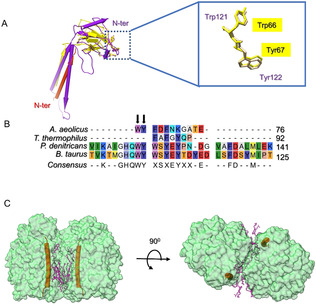
Structures of Subunit II (CoxB2) and Subunit III (IIa). A) Comparison of subunit II from *A. aeolicus* (yellow) and *P. denitrificans* (PDB entry 1AR1, magenta). Subunit IIa from *A. aeolicus* is shown as pipes‐and‐planks representation in red. The N‐termini of subunit IIa from *A. aeolicus* and of subunit II from *P. denitrificans* are marked. The location of the conserved Trp and Tyr is shown on the right in a zoomed‐in view. B) Sequence alignment of subunit IIs from *A. aeolicus*, *T. thermophilus*, *P. denitrificans*, and *B. taurus*. The conserved Trp and Tyr residues are marked by arrows. C) Subunit IIa (orange) is located at the dimer interface, which is occupied by many lipid molecules.

Subunit II (CoxB2) contains one TMH and a ten‐stranded β‐barrel (Figure 3 A). The β‐barrel forms a polar domain that is located at the periplasmic side. The binuclear Cu_A_ center is bound by the conserved residues His96, His139, Cys131, and Cys135. The distance between the two copper atoms is 2.7 Å. The conserved residues Trp121 and Tyr122 in *P. denitrificans* were proposed to play important roles in the electron transfer from cytochrome *c* to Cu_A_.^[11]^ These two conserved residues are also observed in CoxB2 as Trp66 and Tyr67 (Figures 3 A,B).

Subunit IIa of AaC*c*O has been identified previously,^[19]^ and the corresponding density was found and traced in our structure. It contains only one TMH that possesses a location identical to that of the first TMH of subunit II in PdC*c*O but with opposite orientation (Figure 3 A). Subunit IIa is involved in the formation of the dimer interface and interacts with lipids and quinone (Figure 3 C).

### The AaC*c*O dimer

**Figure 4:**
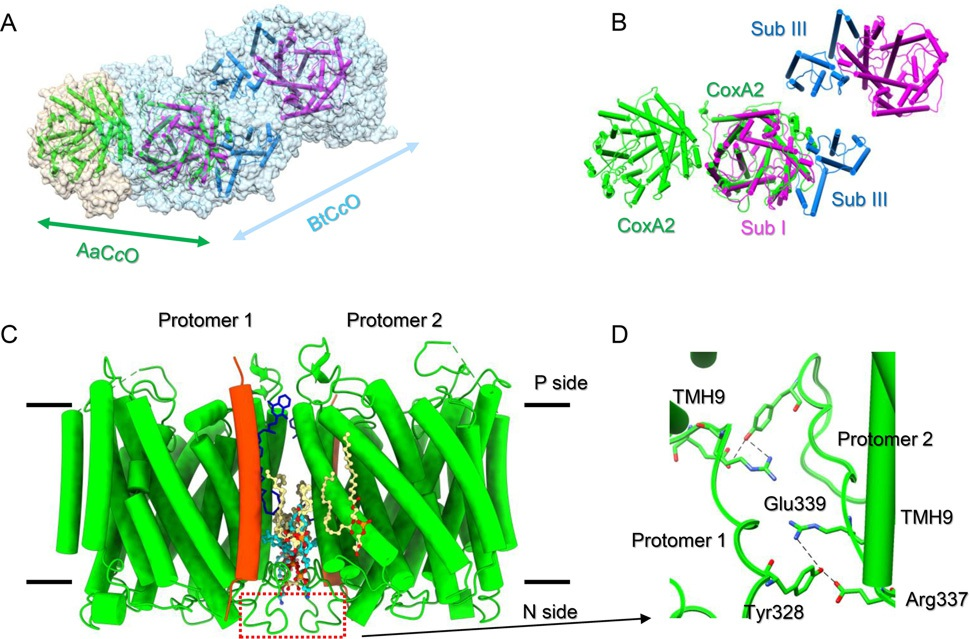
The dimer interfaces. A) Superimposed structures of AaC*c*O and BtC*c*O are illustrated as light tan and sky‐blue transparent surfaces, respectively. AaC*c*O subunit I (CoxA2), BtC*c*O subunit I (Sub I), and subunit III (Sub III) are represented as cartoon and colored in green, purple, and light blue, respectively. The view is perpendicular to the membrane. B) The same view of (A) without showing the surfaces. C) The dimerization of AaC*c*O is mediated by both lipid–protein and protein–protein interactions. Lipid molecules PG, PE, and the quinone molecule NQ are colored in yellow, cyan and blue, respectively. CoxA2 is colored in green. The view is along the membrane. D) Zoomed‐in view of protein–protein interactions at the dimer interface (C). Dotted lines indicate hydrogen bonds or electrostatic interactions.

A comparison of the AaC*c*O dimer structure with the BtC*c*O dimer structure (PDB entry 2OCC) shows that their dimer interfaces are completely different (Figure 4 A). The AaC*c*O dimer is formed via interactions of its major subunit CoxA2 while this is not the case for BtC*c*O (Figure 4 B). Few protein–protein but fruitful protein–lipid interactions are observed in the dimeric interface. Strong hydrogen bond networks between protomers are observed among residues Tyr328, Arg337 and Glu339 at the loop region between TMH8 and TMH9 (Figures 4 C,D). A hydrophobic cavity is found in the interface near the cytoplasmic side, which is occupied by many lipid molecules (Figure S4). Two PEs (phosphatidylethanolamine) and two PGs (phosphatidylglycerol) lipid molecules are identified in the cavity (Figure 4 C). And four more PGs are found at the vicinity (Figure 4 C). Interestingly, two quinol molecules (NQ) are found at the interface with the head group orientation towards the P‐side (Figure 4 C). Our subsequent lipidomics mass spectrometry analysis of co‐purified lipids in the sample confirmed the exact chemical composition of PE and PG and the mass spectrometry analysis of native *A. aeolicus* membranes identified the native quinol molecule as VII‐tetrahydromultiprenyl‐1,4‐naphthoquinone^[23]^ (Figure S6).

### The NQ binding site

**Figure 5:**
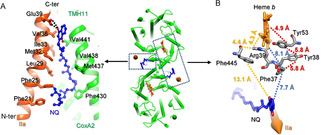
Potential quinol binding site of AaC*c*O. A) The quinone molecule NQ is buried between subunit IIa (orange) and TMH11 (green) of CoxA2. B) The distances between NQ of one protomer and the residues/heme *b* of another protomer are measured, showing potential electron transfer pathways from NQ to heme *b*.

We previously reported that AaC*c*O can use both reduced cytochrome *c* and quinol as electron donors.^[19]^ We originally hypothesized it would be caused by formation of a supercomplex between AaC*c*O and complex III, providing additional quinol binding sites for its direct oxidation bypassing cytochrome *c*. However, our structural study of the *A. aeolicus* complex III^[20]^ does not support this hypothesis. Furthermore, our substantial image processing of the current purified sample does not suggest the existing of any potential supercomplex (Figure S2). Thus, oxidation of NQH_2_ most likely occurs in the AaC*c*O itself, which can be proved by the dimeric structure of AaC*c*O and the existence of NQ molecules at the dimer interface (Figure S4). Each NQ molecule is deeply buried in the hydrophobic groove formed by subunits IIa and coxA2 (Figure 5 A). Many hydrophobic residues interact with the NQ aliphatic chain, including Val36, Ile33, Met32, Leu29, Phe25, and Phe21 of IIa and Phe430, Met437, Val438, Val441 in TMH11 of CoxA2. In particular, one carbonyl oxygen of NQ is found to bind to Glu39 of subunit IIa (Figure 5 A), a residue presents only in *A. aeolicus* (Figure S7A). A deprotonated Glu39 would form a strong hydrogen bond with the hydroxyl group of NQH_2_ and accept one proton upon oxidation of NQH_2_. To be noted, the NQ tail is buried inside one protomer while its carbonyl oxygen is proximal to heme *b* of another protomer (Figures 5 A,B). The edge‐to‐edge distance between NQ and heme *b* is 15.0 Å. With this distance direct electron transfer is possible. In addition, the existence of several aromatic residues (Phe37, Try38, Tyr53, and Phe445) would be also possible involved in the electron transfer from NQH_2_ to heme *b* (Figure 5 B). This quinol binding pocket is similar to the menaquinol binding pocket in cytochrome *aa*_3_‐600 menaquinol oxidase from *Bacillus subtilis* (Figure S7B).^[24]^

### Only a K proton pathway exists in AaC*c*O

**Figure 6:**
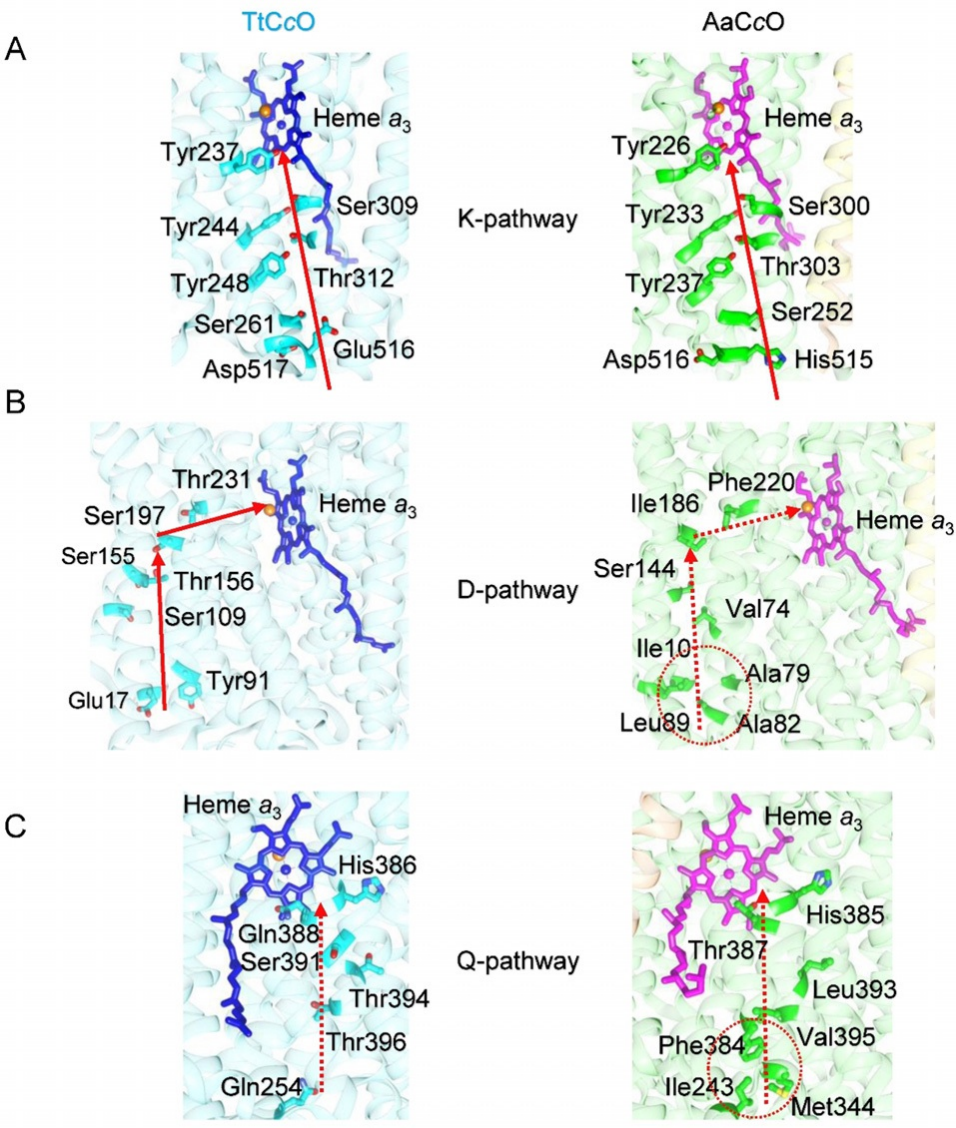
Only the K‐pathway proton channel exists in AaC*c*O. A) The K‐pathway channels of TtC*c*O (left) and AaC*c*O (right) are shown with the red arrow from the cytoplasmic side to the heme *a*_3_ site. B) and C) The potential D‐pathway (B) and Q‐pathway (C) proton channels are blocked in AaC*c*O by various hydrophobic residues. The presumable directions of proton transfer are indicated by red dotted arrows.

Based on the structure of a B‐family C*c*O, the presence of three possible proton pathways, named K‐, D‐, and Q‐pathways, were previously suggested.^[7]^ The residues for forming these pathways are usually conserved between different species with only a limited number of mutations. The K‐pathway, named after its essential lysine residue Lys354, was identified previously.^[25]^ Structural superposition of the crystal structure of TtC*c*O and the cryo‐EM structure of AaC*c*O reveals the presence of the K‐pathway in AaC*c*O, except that at the start Glu516 in the subunit I of TtC*c*O is mutated to His515 in AaC*c*O (Figure 6 A). In this pathway, protons can be transferred from His515 and Asp516 at the N side, via Ser252, Tyr237, Thr303, Tyr233, Ser300, and Tyr226 to the active site that is formed by the high spin heme *a*_3_ and Cu_B_. In the classical K‐pathway, there is usually a conserved Glu of subunit II as a potential proton entry point,^[26]^ which is Glu15 in the subunit II of TtC*c*O and also conserved in AaC*c*O (Glu 5 of subunit II, Figure S8).

Structural superposition also reveals the potential D‐ and Q‐pathway of AaC*c*O (Figures 6 B,C). In the D‐pathway of TtC*c*O, protons can be transferred from Glu17 on the cytoplasmic side, via Tyr91, Ser109, Ser155, Thr156, Ser197, Thr231, and several water molecules, to the heme *a*_3_ active site. However, in AaC*c*O, the entrance for protons is blocked by several hydrophobic residues, including Ala82, Leu89, Ala79, and Ile10. Furthermore, replacing hydrophilic resides to hydrophobic ones Val74, Ile186, and Phe220 does not allow proton transfer (Figure 6 B). A similar situation was also found for the potential Q‐pathway of AaC*c*O (Figure 6 C). Thus, only the K‐pathway does exist for proton transfer in AaC*c*O.

### An unobstructed oxygen diffusion pathway in AaC*c*O

**Figure 7:**
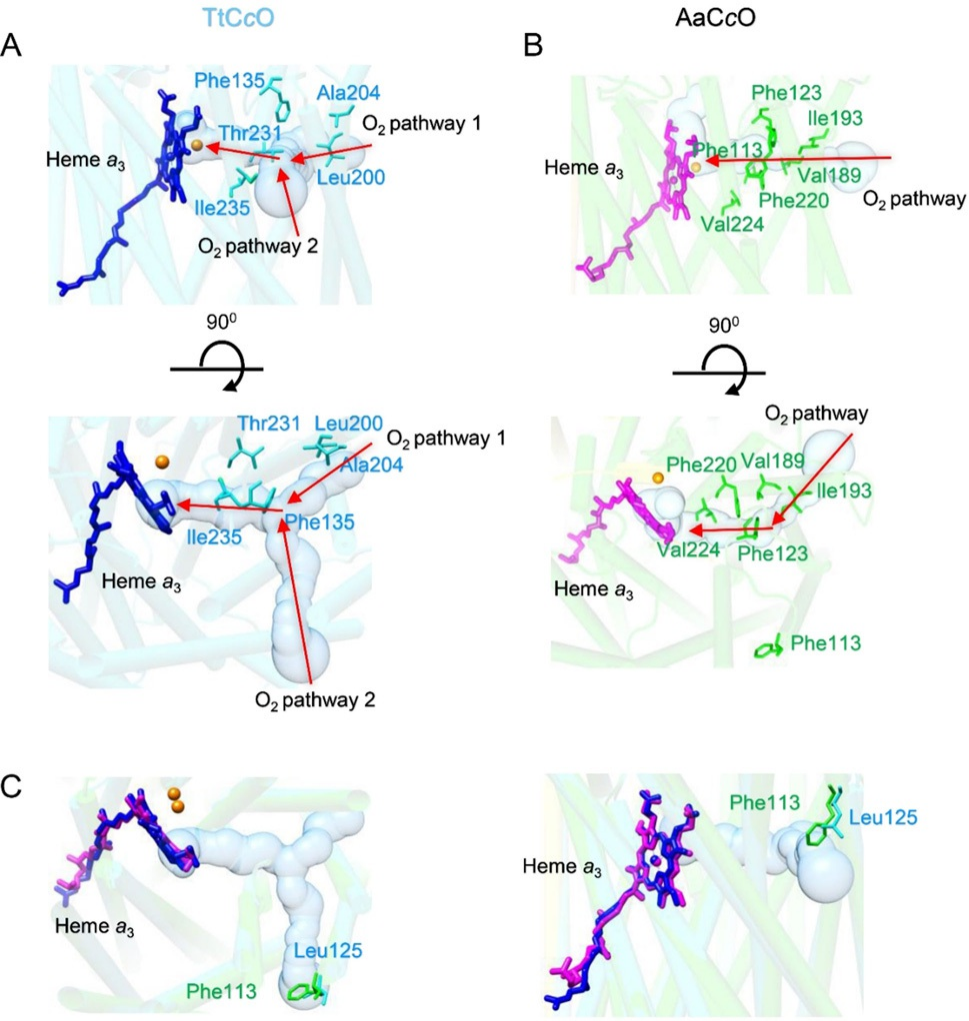
Oxygen channel in AaC*c*O. A) Y‐shaped oxygen channel with two entry points in TtC*c*O is indicated by a solid surface. The entry point 1 starts near Ala204 and Leu200. B) V‐shaped oxygen channel in AaC*c*O. The entry point 1 starts near Ile193 and Val189. C) The entry point 2 near Leu125 in TtC*c*O is blocked by Phe133 in AaC*c*O. Important residues are indicated and shown as sticks. The oxygen channels were calculated and predicted using MOLE2.^[27]^

Based on the crystal structure of TtC*c*O, the presence of a Y‐shaped oxygen diffusion pathway with two entry points was suggested^[7]^ (Figure 7 A). Interestingly, in AaC*c*O one V‐shaped potential oxygen diffusion pathway is observed (Figure 7 B). There is only one entry point to this diffusion pathway, which starts at the middle of the membrane. In this pathway, O_2_ enters a hydrophobic gate formed by Ile194, Ile193, Val138, and Leu135, turning at Phe220, Phe123, Val65, Ile66, and Try121, then passes near Trp228, Val224, Val225, Phe220, Phe217, Trp218 to reach the heme *a*_3_ active center (Figure 7 B). Sequence alignment shows that most of the residues lining the putative oxygen pathway are conserved, except Phe113 in AaC*c*O (Figure S9). A structural superposition of TtC*c*O and AaC*c*O reveals that the oxygen entry point 2 might be blocked by Phe113 in AaC*c*O (Figure 7 C).

## Discussion

The structures and functions of respiratory complexes from different species have been extensively studied in past years.^[28]^ Compared to conventional cytochrome *c* oxidases that use cytochrome *c* as the electron donor, AaC*c*O can directly oxidize quinol substrates besides cytochrome *c*.^[19]^ In the present study, we explored the high‐resolution structure of AaC*c*O by single particle cryo‐EM and got insights into the molecular mechanism of how AaC*c*O could use both cytochrome *c* and quinol as electron donors by discovering the existence of the native quinol molecules NQs bound at the dimeric interface. The edge‐to‐edge distance from NQ to heme *b* is close enough to enable a direct electron transfer from NQH_2_ to the active binuclear center via heme *b*. The proximal aromatic residues between NQ and heme *b* would presumably enhance the rate of electron transfer. Subunit IIa was found to be important for NQ binding by ligation of the head group of NQ and the residue Glu39 of Subunit IIa presumably plays a role of stabilizing NQ during electron transfer. Notably, such electron transfer could only happen between NQ bound to one protomer and heme *b* of another protomer. Thus, the dimerization of AaC*c*O not only provides a new interface for NQ binding but also be necessary for direct electron transfer from NQ. Any regulation factor including thermal fluctuation that alters the formation of AaC*c*O dimer might affect its activity of direct NQH_2_ oxidation, which could likely explain the low quinol oxidation activity measured previously.^[19]^

Previous studies found BtC*c*O to form a homodimer in the crystals^[29]^ while it appears as a monomer in all supercomplex structures.^[30]^ A recent cryo‐EM study discovered another intact 14^th^ subunit (NDUFA4) of human cytochrome *c* oxidase, which is important to keep it in a monomeric active form but was absent in the previous dimeric less active form.^[31]^ At the same time, the structure of an active monomeric form of BtCcO was also determined by X‐ray crystallography.^[32]^ Our present work does not rule out the existence of a supercomplex in *A. aeolicus*, which has been suggested in a previous study.^[33]^ However, the insensitivity of ubiquinol oxidation activity of the potential supercomplex to stigmatellin indicated that AaC*c*O itself has the activity of ubiquinol oxidation.^[19]^ After solving the structure of AaC*c*O, its ubiquinol oxidation activity could be only explained by its dimeric form. Furthermore, this dimeric form is different from that of all other reported C*c*O dimers. We also superimposed the dimeric structure of AaC*c*O into other reported respiratory supercomplexes from *Mycolicibacterium smegmatis* (*M. smegmatis*),^[34]^ *Saccharomyces cerevisiae* (*S. cerevisiae*)^[28e]^ and *Sus scrofa* (*S. scrofa*),^[35]^ and found the dimeric interface of AaC*c*O does not overlap the interface between complex III and complex IV in the supercomplexes from *M. smegmatis* and *S. scrofa* (Figure S10). Thus, to form a supercomplex, such dimeric form of AaC*c*O would not need to be broken.

Considering the hyperthermophilic growth environment of *A. aeolicus*, it would be interesting to investigate the unique structural features of its respiratory chain complex and understand the mechanism of structural adaptation suitable for hyperthermophilic environment. In our previous study of *A. aeolicus* complex III, we discovered an extra transmembrane helix of cyt. *c*_1_ and several unique residues important for the thermostability of the complex.^[20]^ Interestingly, we also found that subunit I of AaC*c*O possesses two additional C‐terminal transmembrane helices, THM13 and TMH14, in comparison with eukaryotic C*c*Os, or still one additional TMH when comparing with TtC*c*O, a thermophilic prokaryotic C*c*O. Thus, it might be possible that the presence of the extra TMHs of AaC*c*O enhances its thermal stability suitable for the hyperthermophilic growth conditions. In addition, the membrane‐anchored cytochrome *c*_555_ might bind to this TMH14, as proposed for the *aa*_3_‐type C*c*O from *P. denitrificans*.^[36]^

Based on the crystal structure of TtC*c*O, three proton transfer pathways (K, D, and Q) were proposed.^[7]^ Mutations of critical residues on D‐pathway (S109A) and Q‐pathway (T396V) showed little influence on the enzymatic activity.[^15^, ^37^] Structural superposition of TtC*c*O and AaC*c*O reveals that the D‐ and Q‐proton pathways in AaC*c*O are both blocked by multiple hydrophobic residues. Therefore, even if the D‐ and Q‐proton pathways in TtC*c*O were active, the same pathways in AaC*c*O should be closed and inactive. Only the K‐pathway appears to be present in AaC*c*O. Besides the proton transfer pathway, the potential oxygen diffusion channel of AaC*c*O also varies in comparison with that of TtC*c*O. Along with conserved oxygen channels being suggested for C*c*Os from *Rhodobacter sphaeroides*,^[38]^ *P. dentrificans* ^[39]^ and *B. taurus*,^[9b]^ a Y‐shaped oxygen channel was also reported in TtC*c*O, suggesting that there are two entry points for oxygen. However, structural superposition of AaC*c*O and TtC*c*O suggest that only one oxygen diffusion channel exists and forms a V‐shape in AaC*c*O. The second oxygen entry point 2 found in TtC*c*O is blocked by residue Phe113 in AaC*c*O at the equivalent position. The larger the void in a protein, the smaller is its stability.^[40]^ The adapted structure of AaC*c*O with only the K proton pathway present and the V‐shaped unobstructed oxygen channel with more hydrophobic residues blocking one entry appears to be evolutionary advantageous to keep the balance between its enzymatic activity and structural stability in the hyperthermophilic environment.

## Conclusion

In summary, we solved the 3.4 Å structure of cytochrome *c* oxidase from the hyperthermophilic bacterium *Aquifex aeolicus*, revealed the molecular mechanism that this oxidase uses both cytochrome *c* and quinol as electron donors, made structural insights into its thermal stability, and suggested an evolutionary adaptation of this oxidase to keep the balance between its enzymatic activity and structural stability for the hyperthermophilic growth condition. These results provide structural basis for molecular mechanism and the evolutionary significance of cytochrome *c* oxidases in an extreme thermal environment.

### Data and materials availability

The atomic coordinates of the cytochrome *c* oxidase of *Aquifex aeolicus* reported in this paper have been deposited in Worldwide Protein Data Bank (PDB) (http://www.rcsb.org) with the accession codes 7DEG. The corresponding maps have been deposited in the Electron Microscope Data Bank (EMDB) (http://emdatabank.org) with the accession codes EMD‐30657.

## Conflict of interest

The authors declare no conflict of interest.

## Supporting information

As a service to our authors and readers, this journal provides supporting information supplied by the authors. Such materials are peer reviewed and may be re‐organized for online delivery, but are not copy‐edited or typeset. Technical support issues arising from supporting information (other than missing files) should be addressed to the authors.

Supplementary

Supplementary
