# Supplementary material for: The Unusual Homodimer of a Heme‐Copper Terminal Oxidase Allows Itself to Utilize Two Electron Donors
Source: Angew Chem Int Ed Engl. 2021 May 6;60(24):13323–30. doi: 10.1002/anie.202016785 (PMC8251803; doi:10.1002/anie.202016785)
Supplement: Supplementary file 1 — Supplementary [file ANIE-60-13323-s002.pdf]

Angewandte Chemie
Eine Zeitschrift der Gesellschaft Deutscher Chemiker

Supporting Information

The Unusual Homodimer of a Heme-Copper Terminal Oxidase Allows Itself to Utilize Two Electron Donors

Guoliang Zhu[+], Hui Zeng[+], Shuangbo Zhang[+], Jana Juli, Linhua Tai, Danyang Zhang, Xiaoyun Pang, Yan Zhang, Sin Man Lam, Yun Zhu,* Guohong Peng,* Hartmut Michel,* and Fei Sun**

anie_202016785_sm_miscellaneous_information.pdf
anie_202016785_sm_Movie_1.mp4

Author Contributions

F. S., H. M., Y. Z. and G. P. started and supervised the project. G. Z., H. Z., S. Z., J. J., L. T., D. Z., X. P., Y. Z., and G. P. performed all the experiments. G. Z. and S. Z. performed image processing and solved the cryo-EM structure. S. M. L. performed mass spectrometry analysis and identified the lipids of the complex. G. Z., H. Z., Y. Z. and G. P. analyzed the data and wrote the paper with the substantial input from H. M. and F. S.

Table of Contents

Experimental Procedures .... S 2
Figure S1. Purification of AaC*c*O........ S 4
Figure S2. Structure determination of AaC*c*O .... S 5
Figure S3. Sequence alignment of subunit I (CoxA2) from different species........ S 6
Figure S4. Representative cryo-EM densities of lipids and prosthetic groups in AaC*c*O .... S 7
Figure S5. Representative cryo-EM densities of AaC*c*O subunits .... S 8
Figure S6. Lipid and quinone identification from *Aquifex aeolicus*. .... S 9
Figure S7. Analysis of the potential quinol binding pocket of AaC*c*O. S10
Figure S8. Sequence alignment of subunit II (CoxB2) from different species S11
Figure S9. Sequence alignment of subunit I (CoxA2) from *A. aeolicus* and *T. thermophilus*. S12
Figure S10. Structural comparisons of respiratory supercomplexes and AaC*c*O dimer. S13
Movie S1 (separate file). The overall map of complex IV dimer in C2 symmetry with model fitted. S14
Table S1. Statistics of data collection, image processing and model building. S15
References S16
Author Contributions S17
Dataset. S1. Full wwPDB/EMDataBank EM Map/Model Validation Report of cytochrome *c* oxidase from *Aquifex aeolicus* S18

Experimental Procedures

Purification and identification of AaC*c*O

20 mL membranes (20 mg/mL) from *Aquifex aeolicus* were solubilized by 2% (w/v) n-dodecyl-β-D-maltoside (DDM) at 52 °C for 1 h with gentle shaking. The solubilized membrane proteins were separated by ultracentrifugation with 50,000 rpm at 4 °C for 1h [1]. The supernatant was applied on a MonoQ 10/100 GL (GE Healthcare) anion exchange column that was equilibrated with buffer containing 20 mM Tris-HCl (pH 7.4), 50 mM NaCl, and 0.05% (w/v) DDM. The solubilized membrane proteins were eluted with linear gradients using 1 M NaCl as eluting salt. The cytochrome *c* oxidase (AaC*c*O) was first eluted with a salt concentration gradient of 60-100 mM NaCl (**Fig. S1A**). The elution was concentrated to 10 mL with concentrators (Amicon Ultra-15, PLQK Ultracel-PL Membran, 50 kDa) and applied to TSK-GEL G4000SW 21.5/30 column (TOSOH Bioscience) connected to an ÄKTA purifier chromatography system (GE Healthcare) with buffer containing 20 mM Tris-HCl (pH 7.4), 150 mM NaCl, and 0.05% (w/v) DDM. The gel filtration chromatography was performed at a flow rate of 0.5 mL/min and absorbance at 280 nm and 415 nm were monitored. The target protein fractions were collected (**Fig. S1B**) and analyzed by anti-CoxA2 western-blot (**Fig. S1C**). The AaC*c*O was further purified using a Yarra SEC-4000 (Phenomenex) gel filtration column with buffer 20 mM Tris-HCl (pH 7.4), 150 mM NaCl, and 0.1% (w/v) digitonin (**Fig. S1D**). The fractions containing AaC*c*O were analyzed by SDS-PAGE (**Fig. S1E**) and Blue Native PAGE (**Fig. S1F**). The fractions 13 and 14 showing a dominant homogenous band around 242 kDa in Blue Native PAGE were used for the subsequent cryo-EM analysis. See **Figure S1** for more details.

Cryo-EM data acquisition

For cryo-EM, 3 µL of the freshly purified complex sample (1.5 mg/mL) was applied to glow-discharged holey carbon film grids (GIG Au R 1/1, 300 mesh), which had been treated under receipts of H_2 and O_2 mixtures in Solarus (Gatan, USA) for 1 min. Grids were blotted for 3s at 16 °C and 100% humidity, then flash frozen in liquid ethane and stored in liquid nitrogen using Vitrobot IV (ThermoFisher Scientific, USA). High resolution images were collected on a 300kV FEI Titan Krios transmission electron microscope (ThermoFisher Scientific, USA) using SerialEM software [2] on K2 detector (Gatan, USA), with super-resolution counting mode at a calibrated magnification of 130,000x, yielding a pixel size of 0.52 Å. A quantum energy filter (Gatan, USA) with energy slot set to 20 eV was applied for zero-loss imaging. Defocus values varied from -1.5 to -2.5 µm. 32 frames per stack were collected with a total exposure time of 7.4s. The dose rate was set to ~9 e^-/pixel/s and the total dose was ~60 e^-/Å^2. Frame alignment, exposure weighting, and contrast transfer function parameters estimation were performed using Center for Biological Imaging (CBI, Institute of Biophysics, Chinese Academy of Sciences) automated data collection scripts integrated with Motioncor2 [3] and CTFFIND4 [4] during data collection.

Image processing

Image processing steps were performed using RELION (version 2.1 and version 3.0-beta) [5] and EMAN2 [6]. Gautomatch (https://www.mrc-lmb.cam.ac.uk/kzhang/Gautomatch/) was used to pick around an initial dataset of 270k particles from 1304 good quality micrographs. They were extracted and sorted by one round of 2D classification. Then 195,313 particles from good 2D classes were selected to generate initial model and for the first round of 3D classification. During 3D classification, C1 symmetry was applied and only two well-aligned classes appeared with one class containing 40.7% portion of the particles and another containing 29% portion of the particles. The first class with 59,117 particles was selected for subsequent image processing, which yielded the 3D reconstruction of the dimer of Complex III reported in our previous study [7]. The second class was selected to yield a subset of 42,128 particles, which were re-extracted with refined particle shifts from the original micrographs using a 256×256 box size. The refinement resulted in a 3D structure with an overall resolution of 6.7 Å. After a second round of 3D classification without alignment, one best class containing 32,982 particles from 14 classes was selected for the final refinement when C2 symmetry was imposed. After refining each particle' defocus value by CtfRefine and motion shift by Bayesian polishing, the overall resolution of the structure was improved to 3.5 Å. Then THUNDER [8] was used to further refine to a final structure with the resolution of 3.4 Å. See **Figure S2** for more details.

Model building and refinement

To build the atomic model of AaC*c*O, the homologous structure of TtC*c*O (PDB entry: 1EHK) [9] was used as an initial model. We were able to trace most regions with side chains using COOT [10]. The ligands and phospholipids were also docked into densities and adjusted using COOT. The built model was further refined in real space using Phenix [11]. All figures were created by Pymol [12], UCSF Chimera [13] and UCSF ChimeraX [14]. The parameters for data collection and structure determination are summarized in **Table S1**.

Lipid and quinol identification by mass spectrometry

For independent lipidomics analysis, co-purified lipids from AaC*c*O sample were extracted with 800 µL of chloroform: methanol: water (1:1:0.1) in glass vials. The samples were incubated at 1500 rpm at 4°C for 30 min, then 350 µL of water were added to induce phase separation. The lower organic phase containing lipids were transferred to new glass vials. Extraction was repeated once with the addition of another 400 µL of chloroform, and the lipid extracts were combined and dry in a SpeedVac. Liquid chromatography-mass spectrometric (LCMS) analyses were carried out on an Exion UPLC coupled with a Sciex QTRAP 6500 Plus as described previously [15]. Normal-phase LCMS analysis of phospholipids was carried out under electrospray ionization mode (ESI) using DMPC, DMPE, DMPG, DMPA and d31-PI (18:1/16:0) purchased from Avanti Polar Lipids as internal standards. Lipid levels were expressed in nmol lipids per g protein.

For quinol identification, homogenized native *A. aeolicus* membrane were mixed with 1 mL mixture of chloroform, methanol and water (8:4:3) in 1.5 mL EP pipe. After inverting the samples about 10 times, the upper water phase was removed. The organic phase was concentrated and analyzed with Triple-TOF mass spectrometer 5600+ (SCIEX, USA) equipped with a Thermofisher U3000 UHPLC. Samples were injected and separated on a waters C18 column ACQUITY CSHTM C18（2.1100 mm, 1.7 µm) with a mobile phase of solution A [MeOH: ACN: H_2O (1:1:1), 5 mM NH_4AC] and solution B [IPA: MeOH (4:1), 5mM NH_4AC] at a flow-rate of 0.25 mL/min as gradient timetable. For normal electrospray ion source setting, the electrospray voltage was set to 5.5 kV, ion source gas 1 was set to 55 psi, ion source gas 2 was set to 55 psi, curtain gas was set to 35 psi and the ion source temperature was set to 550 °C. Survey full scan MS spectra were acquired across the mass range of 50~1250 m/z with CE35.

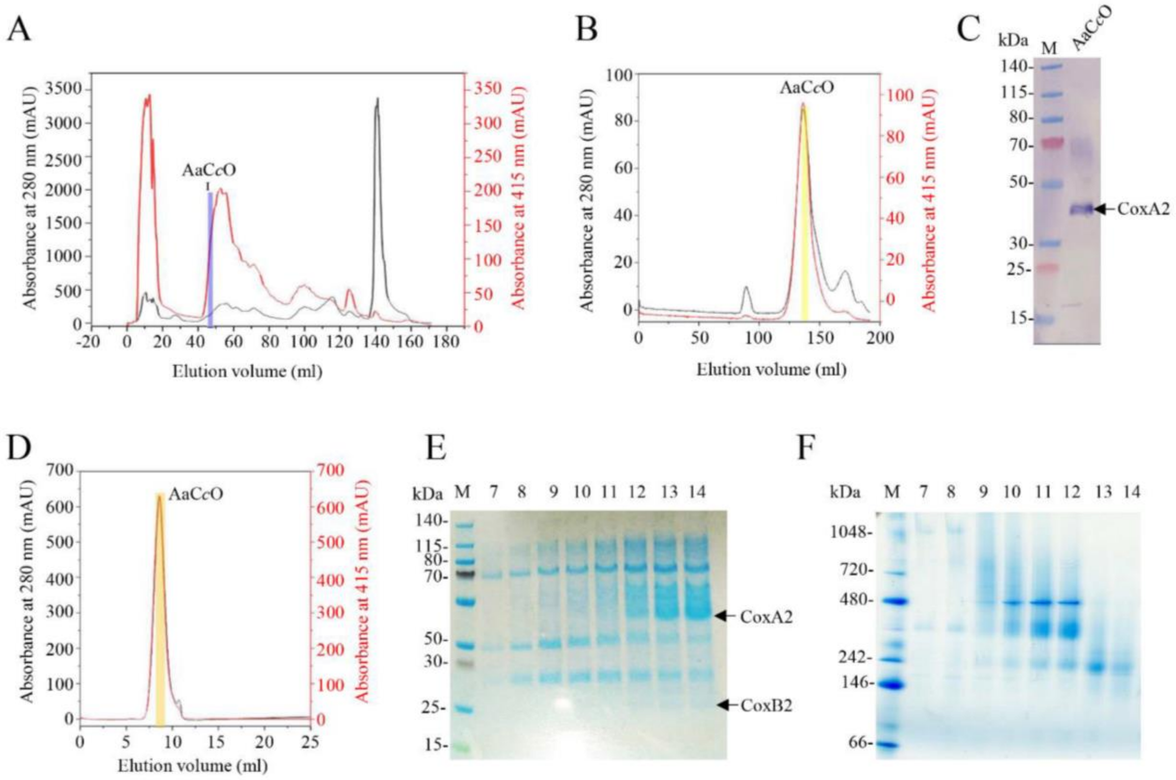

Figure S1. Purification of AaC*c*O. (A) Separation of native *A. aeolicus* membrane proteins by ion exchange chromatography. Fraction 'I' colored in purple was used to purify AaC*c*O. **(B)** The fraction 'I' in (A) was further purified by size exclusion chromatography with TSK-GEL G4000SW 21.5/30 SEC column. The fraction colored in yellow was pooled and used to purify native AaC*c*O. **(C)** The pooled sample in (B) was analyzed by western blot using anti-CoxA antibody. M: PageRuler Prestained protein ladder. Bands of interest CoxA2 is marked on the right. **(D)** Size exclusion chromatography profile of native AaC*c*O with Yarra SEC-4000. The detergent was shift from 0.05% (w/v) DDM to 0.1% digitonin. Fractions 13 and 14 are colored in orange. **(E)** Peak fractions (7,8,9,10,11,12,13 and 14) in (D) were analysed by SDS-PAGE. Bands of interest CoxA2 and CoxB2 are marked on the right. M: PageRuler Prestained protein ladder. **(F)** Peak fractions in (D) were also analysed by blue native PAGE. The molecular weights (kDa) of the marker proteins (lane M) are indicated on the left.

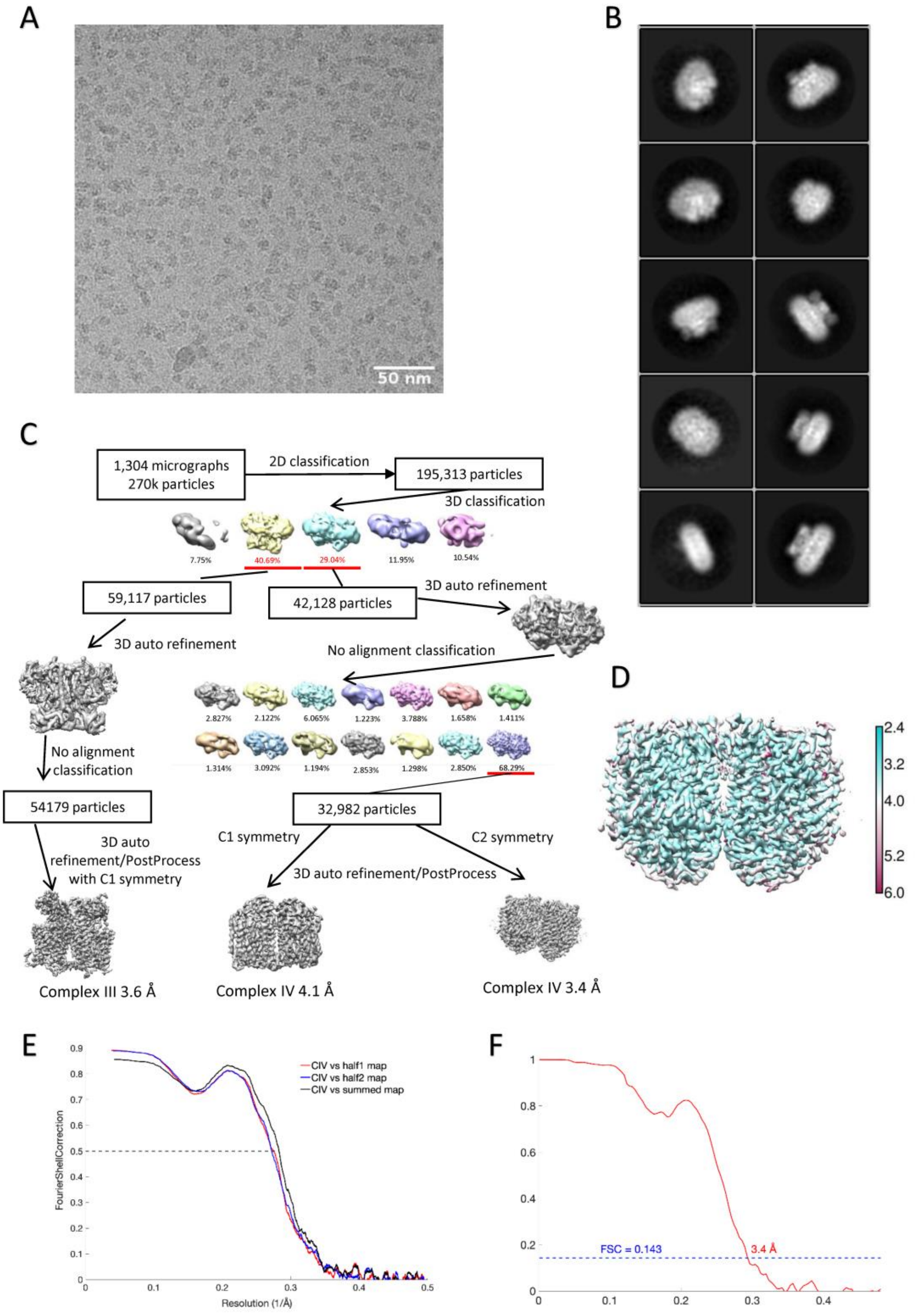

Figure S2. Structure determination of AaC*c*O. (A) Representative cryo-EM micrograph of AaC*c*O sample. **(B)** Representative 2D class averages obtained from reference-free classification. **(C)** Workflow of 3D classification and reconstruction. **(D)** Local resolution analysis of complex IV dimer in C2 symmetry. **(E)** The map-to-model FSC curve of complex IV dimer in C2 symmetry. **(F)** Gold-standard FSC curve for the overall map of complex IV dimer in C2 symmetry.

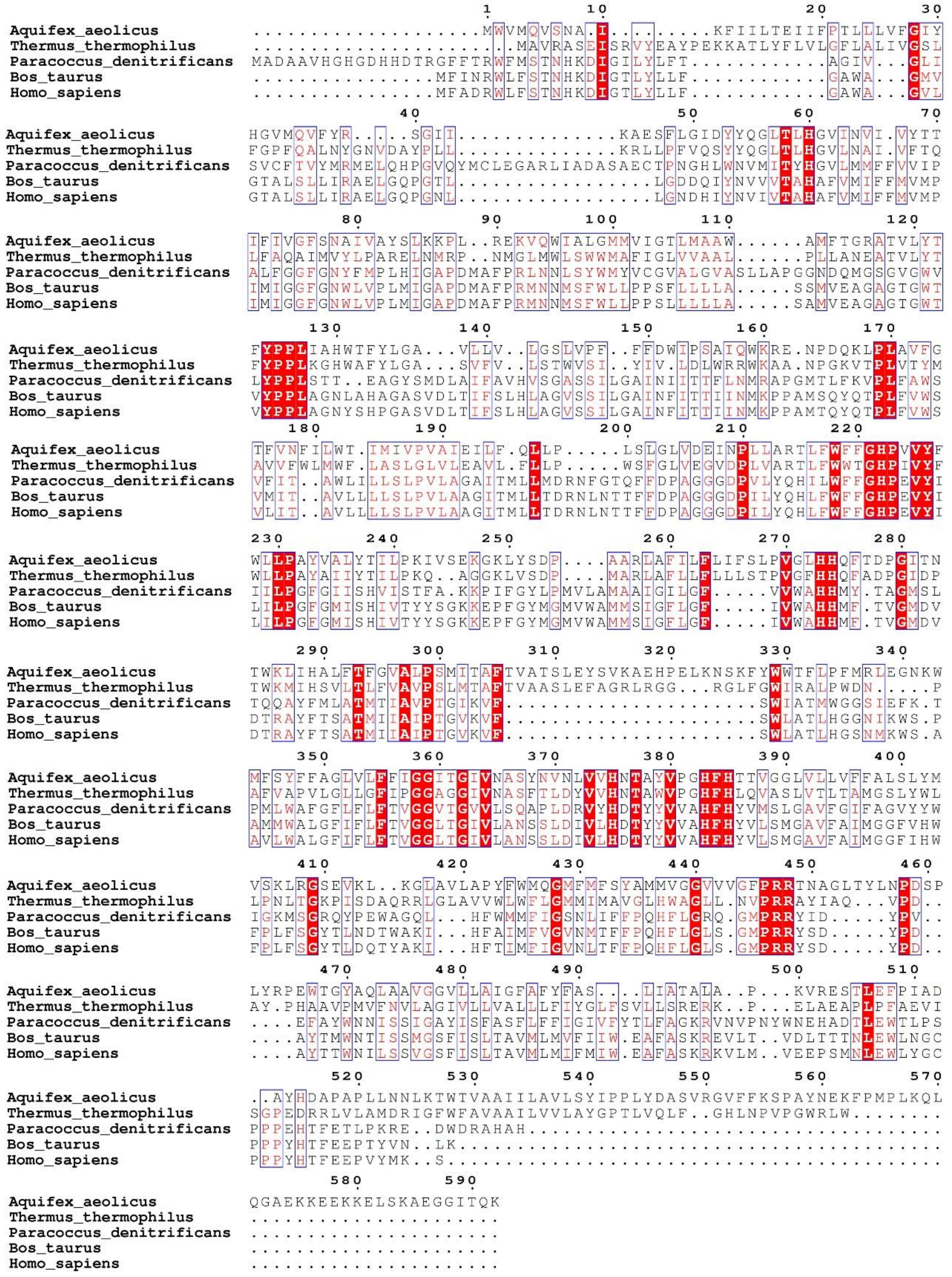

Figure S3. Sequence alignment of subunit I (CoxA2) from different species.

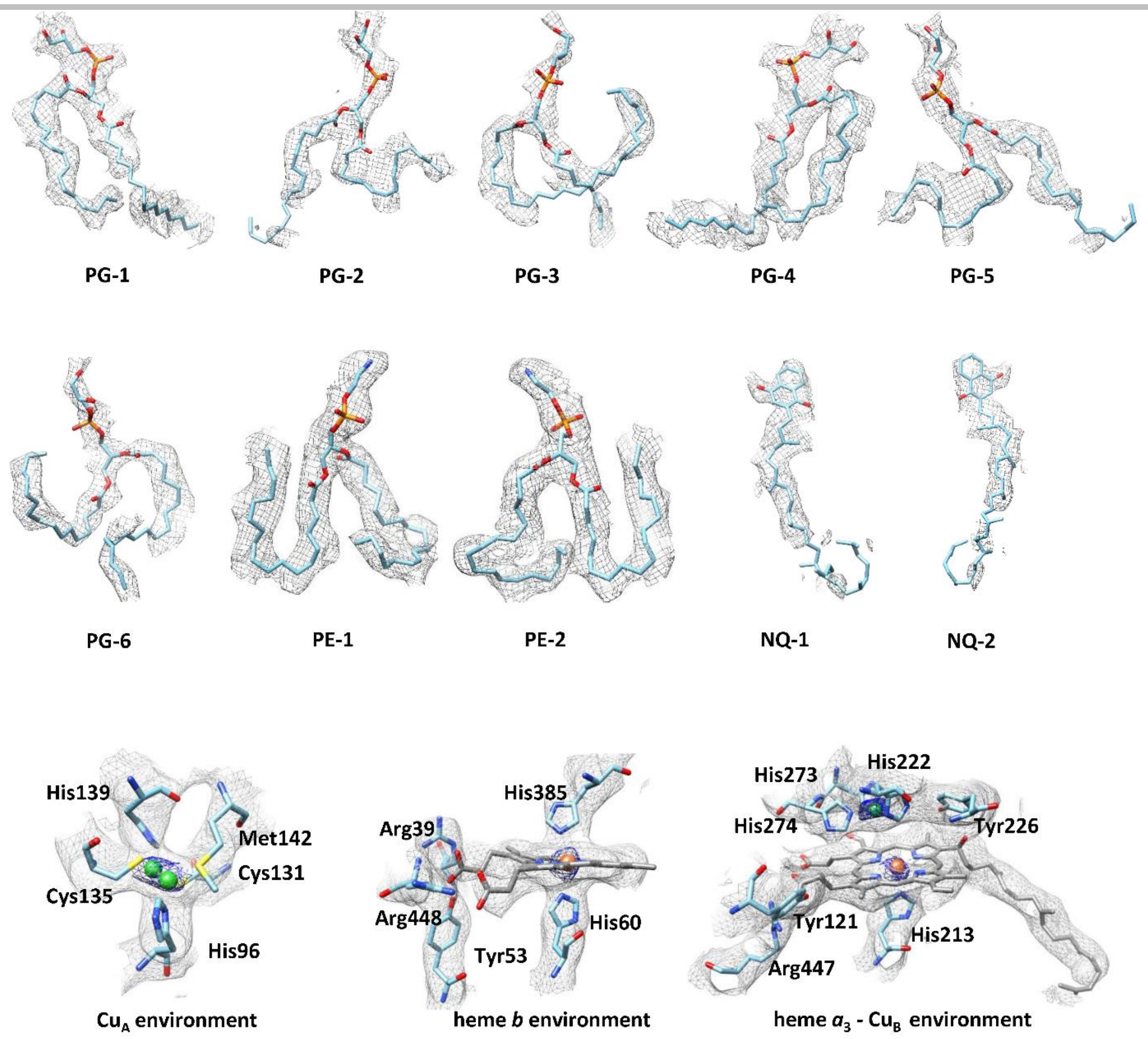

Figure S4. Representative cryo-EM densities of lipids and prosthetic groups in AaC*c*O.

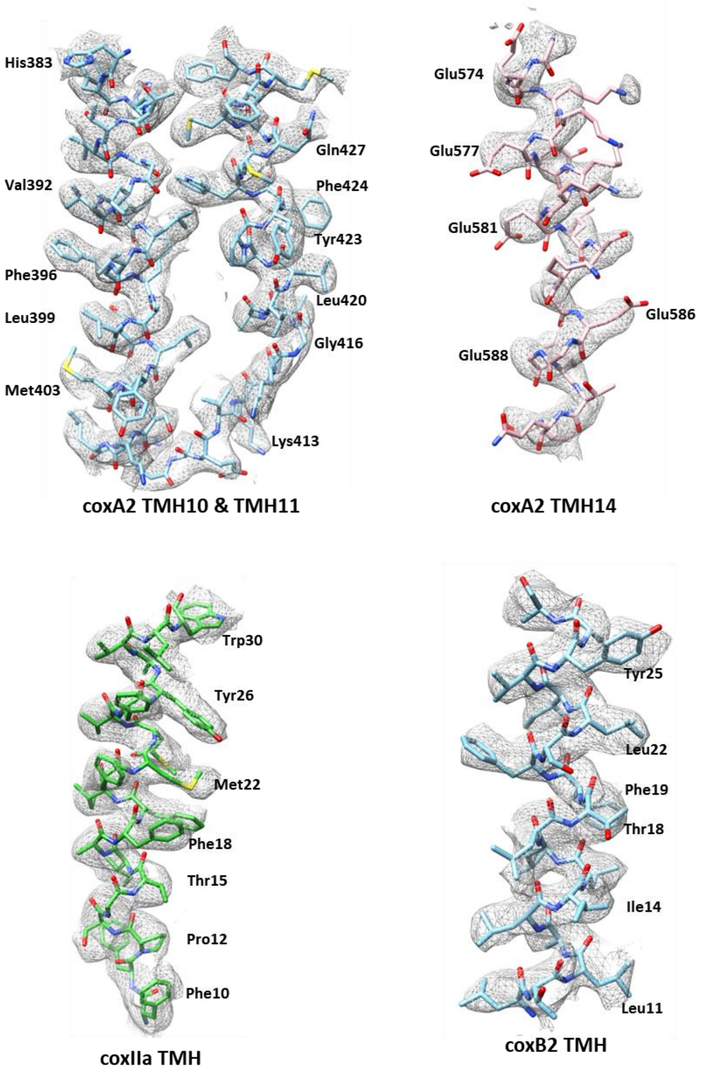

Figure S5. Representative cryo-EM densities of AaC*c*O subunits.

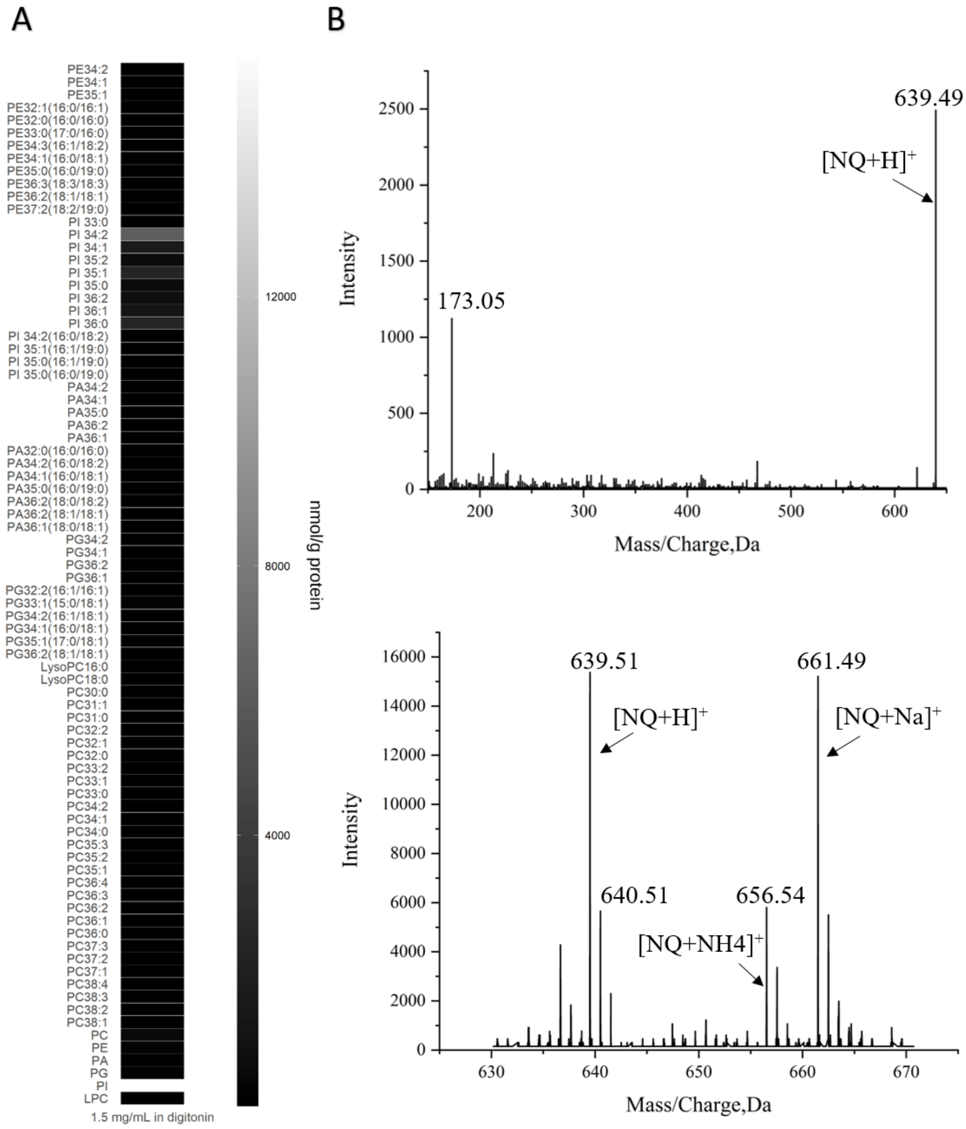

Figure S6. Lipid and quinone identification from *Aquifex aeolicus*. (A) The lipidomics analysis of the lipids co-purified with AaC*c*O. The heatmap shows all lipids that are detected and lipid levels are expressed in nmol/g protein. **(B)** The mass spectrum of naphthoquinone from the native membrane of *Aquifex aeolicus*. The MS/MS profile show fragment ion peaks at m/z values 173.05, 639.49, 656.54, and 661.49 corresponding to the naphthoquinone characteristic fragments.

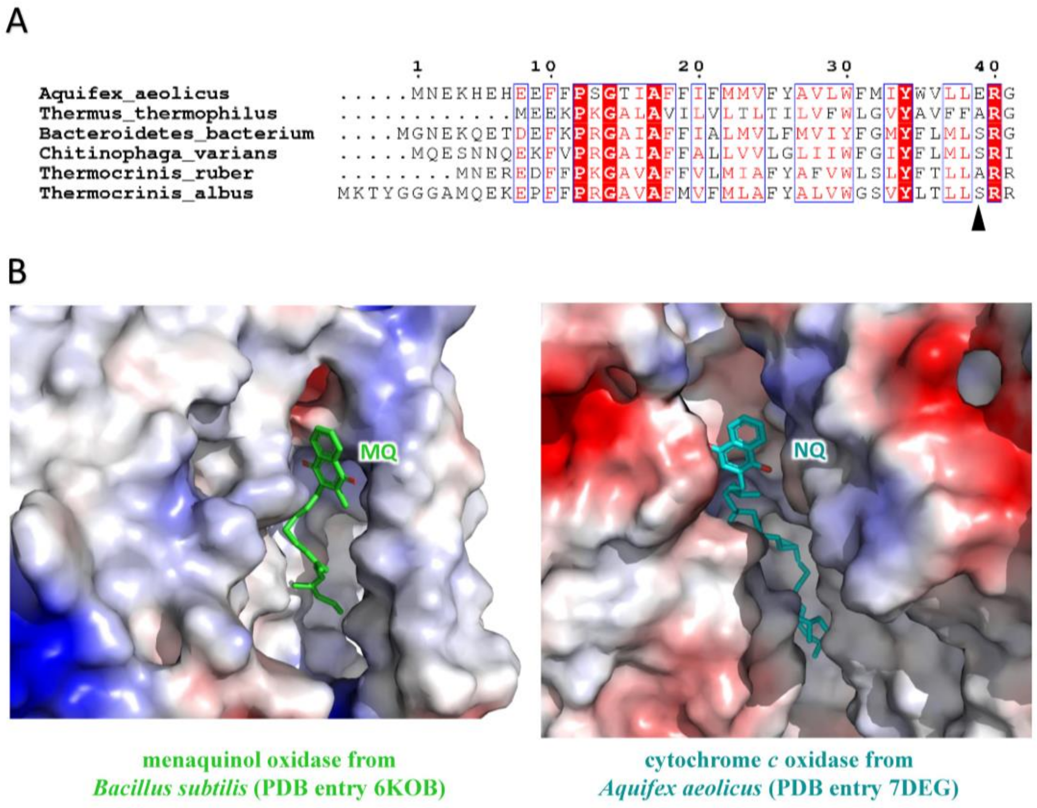

Figure S7. Analysis of the potential quinol binding pocket of AaC*c*O. (**A**) Sequence alignment of subunit IIa from different species. The unique Glu39 of AaC*c*O subunit IIa for quinol binding is labelled. (**B**) Comparison of quinol binding pocket in cytochrome *aa*$_3$-600 menaquinol oxidase from *Bacillus subtilis* (PDB entry 6KOB) [16] and that in the dimeric interface of AaC*c*O.

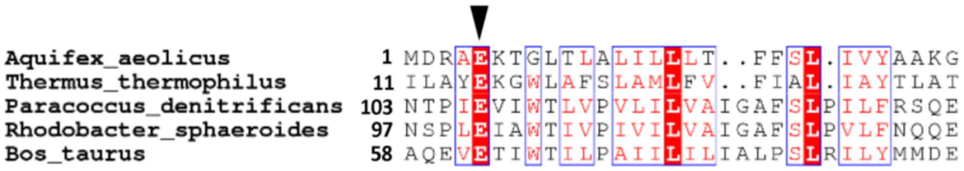

Figure S8. Sequence alignment of subunit II (CoxB2) from different species. The potential proton entrance of K-pathway is indicated by the black arrowhead.

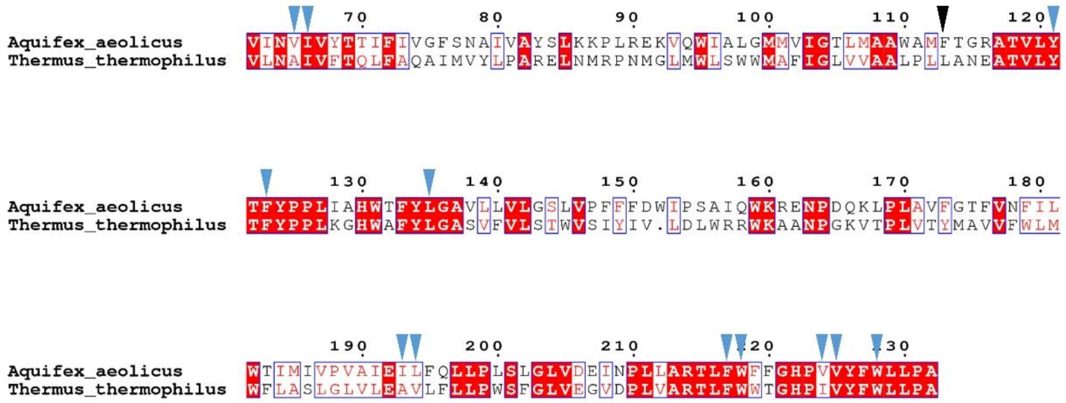

Figure S9. Sequence alignment of subunit I (CoxA2) from *A. aeolicus* and *T. thermophilus*. The blue arrowheads indicate the conserved residues lining the putative oxygen pathway, while the specific Phe113 is indicated by the black arrowhead.

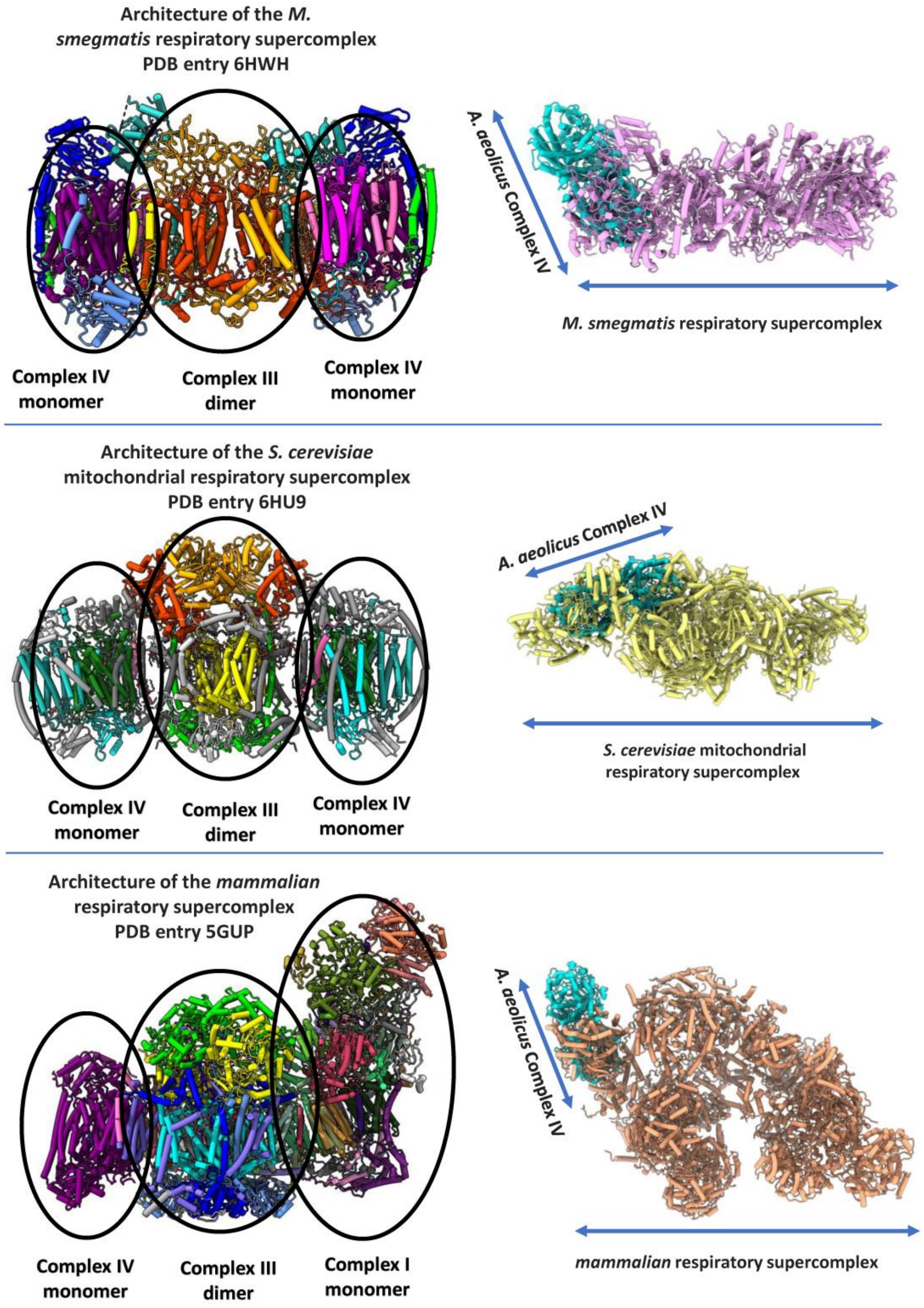

Figure S10. Structural comparisons of respiratory supercomplexes and AaC*c*O dimer. The overall architectures of several reported respiratory supercomplexes are shown on the left (color by chain), and the superimposing of AaC*c*O dimeric structure (cyan) into these structures are shown on the right, including that from *Mycolicibacterium smegmatis* (*M. smegmatis*) (pink) [17], *Saccharomyces cerevisiae* (*S. cerevisiae*) (yellow) [18] and *Sus scrofa* (*S. scrofa*) (orange) [19], respectively.

Movie S1. The overall map of complex IV dimer in C2 symmetry with model fitted. Views are sliced to show the overall quality of the map and the fitted model.

Table S1. Statistics of data collection, image processing and model building

Sample	The respiratory complex IV from *A. aeolicus*
Data collection	
EM equipment	FEI Titan Krios
Voltage (KV)	300
Detector	Gatan Bioquantum K2
Energy filter	20 eV
Pixel size (Å/pixel)	1.04
Electron dose (e^-/Å^2)	60
Defocus range (μm)	-1.5~-2.5
Reconstruction	
Software	RELION 3.0-beta / RELION 2.0
Number of used particles	32982
Accuracy of rotation	2.526
Accuracy of translations (pixel)	0.891
Symmetry	C2
Map sharpening B-factor (Å^2)	-85
Final resolution (Å)	3.41
Model building	
Software	Coot
Model Refinement	
Software	PHENIX
Map CC (whole unit cell)	0.85
Map CC (around atoms)	0.71
Rmsd (bonds) (Å)	0.016
Rmsd (angle) (°)	1.528
Model composition	
Protein residues	1520
Heme groups	4
Cu center	4
Validation	
Ramachandran plot	
Outliers (%)	0.40
Allowed (%)	16.56
Favored (%)	83.05
Rotamer outliers (%)	1.10

References

[1] G. Peng, G. Fritzsch, V. Zickermann, H. Schagger, R. Mentele, F. Lottspeich, M. Bostina, M. Radermacher, R. Huber, K. O. Stetter, H. Michel, *Biochemistry* **2003**, *42*, 3032-3039.
[2] D. N. Mastronarde, *J Struct Biol* **2005**, *152*, 36-51.
[3] S. Q. Zheng, E. Palovcak, J.-P. Armache, K. A. Verba, Y. Cheng, D. A. Agard, *Nature methods* **2017**, *14*, 331.
[4] A. Rohou, N. Grigorieff, *Journal of structural biology* **2015**, *192*, 216-221.
[5] aS. H. W. Scheres, *J Struct Biol* **2012**, *180*, 519-530; bD. Kimanius, B. O. Forsberg, S. H. Scheres, E. Lindahl, *Elife* **2016**, *5*, e18722.
[6] G. Tang, L. Peng, P. R. Baldwin, D. S. Mann, W. Jiang, I. Rees, S. J. Ludtke, *Journal of structural biology* **2007**, *157*, 38-46.
[7] G. L. Zhu, H. Zeng, S. B. Zhang, J. Juli, X. Y. Pang, J. Hoffmann, Y. Zhang, N. Morgner, Y. Zhu, G. H. Peng, H. Michel, F. Sun, *Angew Chem Int Edit* **2020**, *59*, 343-351.
[8] M. Hu, H. Yu, K. Gu, Z. Wang, H. Ruan, K. Wang, S. Ren, B. Li, L. Gan, S. Xu, G. Yang, Y. Shen, X. Li, *Nat Methods* **2018**, *15*, 1083-1089.
[9] T. Soulimane, G. Buse, G. P. Bourenkov, H. D. Bartunik, R. Huber, M. E. Than, *EMBO J* **2000**, *19*, 1766-1776.
[10] P. Emsley, K. Cowtan, *Acta Crystallogr D Biol Crystallogr* **2004**, *60*, 2126-2132.
[11] P. D. Adams, P. V. Afonine, G. Bunkoczi, V. B. Chen, I. W. Davis, N. Echols, J. J. Headd, L. W. Hung, G. J. Kapral, R. W. Grosse-Kunstleve, A. J. McCoy, N. W. Moriarty, R. Oeffner, R. J. Read, D. C. Richardson, J. S. Richardson, T. C. Terwilliger, P. H. Zwart, *Acta Crystallogr D Biol Crystallogr* **2010**, *66*, 213-221.
[12] Schrodinger, LLC, **2015**.
[13] E. F. Pettersen, T. D. Goddard, C. C. Huang, G. S. Couch, D. M. Greenblatt, E. C. Meng, T. E. Ferrin, *J Comput Chem* **2004**, *25*, 1605-1612.
[14] E. F. Pettersen, T. D. Goddard, C. R. C. Huang, E. E. C. Meng, G. S. Couch, T. I. Croll, J. H. Morris, T. E. Ferrin, *Protein Sci* **2020**.
[15] H. R. Gong, J. Li, A. Xu, Y. T. Tang, W. X. Ji, R. G. Gao, S. H. Wang, L. Yu, C. L. Tian, J. W. Li, H. Y. Yen, S. M. Lam, G. H. Shui, X. N. Yang, Y. N. Sun, X. M. Li, M. Z. Jia, C. Yang, B. Jiang, Z. Y. Lou, C. V. Robinson, L. L. Wong, L. W. Guddat, F. Sun, Q. Wang, Z. H. Rao, *Science* **2018**, *362*, 1020-+.
[16] J. Xu, Z. Ding, B. Liu, S. M. Yi, J. Li, Z. Zhang, Y. Liu, J. Li, L. Liu, A. Zhou, R. B. Gennis, J. Zhu, *Proc. Natl. Acad. Sci. USA* **2020**, *117*, 872-876.
[17] B. Wiseman, R. G. Nitharwal, O. Fedotovskaya, J. Schafer, H. Guo, Q. Kuang, S. Benlekbir, D. Sjostrand, P. Adelroth, J. L. Rubinstein, P. Brzezinski, M. Hogbom, *Nat Struct Mol Biol* **2018**, *25*, 1128-1136.
[18] A. M. Hartley, N. Lukoyanova, Y. Zhang, A. Cabrera-Orefice, S. Arnold, B. Meunier, N. Pinotsis, A. Marechal, *Nat Struct Mol Biol* **2019**, *26*, 78-83.
[19] M. Wu, J. Gu, R. Guo, Y. Huang, M. Yang, *Cell* **2016**, *167*, 1598-1609 e1510.

Author Contributions

F. S., H. M., Y. Z. and G. P. started and supervised the project. G. Z., H. Z., S. Z., J. J., L. T., D. Z., X. P., Y. Z., and G. P. performed all the experiments. G. Z. and S. Z. performed image processing and solved the cryo-EM structure. S.M.L. performed mass spectrometry analysis and identified the lipids of the complex. G. Z., H. Z., Y. Z. and G. P. analyzed the data and wrote the paper with the substantial input from H. M. and F. S.

Full wwPDB EM Map/Model Validation Report (i)

Nov 13, 2020 – 05:37 PM JST

PDB ID	:	7DEG
EMDB ID	:	EMD-30657
Title	:	Cryo-EM structure of a heme-copper terminal oxidase dimer provides insights into its catalytic mechanism
Deposited on	:	2020-11-04
Resolution	:	3.40 Å(reported)

This is a Full wwPDB EM Map/Model Validation Report.

This report is produced by the wwPDB biocuration pipeline after annotation of the structure.

We welcome your comments at *validation@mail.wwpdb.org*
A user guide is available at
https://www.wwpdb.org/validation/2017/EMValidationReportHelp
with specific help available everywhere you see the (i) symbol.

CONFIDENTIAL VALIDATION REPORT

The following versions of software and data (see references (i)) were used in the production of this report:

EMDB validation analysis	:	0.0.0.dev33
Mogul	:	1.8.5 (274361), CSD as541be (2020)
MolProbity	:	4.02b-467
buster-report	:	1.1.7 (2018)
Percentile statistics	:	20191225.v01 (using entries in the PDB archive December 25th 2019)
Ideal geometry (proteins)	:	Engh & Huber (2001)
Ideal geometry (DNA, RNA)	:	Parkinson et al. (1996)
Validation Pipeline (wwPDB-VP)	:	2.14.6

1 Overall quality at a glance (i)

The following experimental techniques were used to determine the structure:
ELECTRON MICROSCOPY

The reported resolution of this entry is 3.40 Å.

Percentile scores (ranging between 0-100) for global validation metrics of the entry are shown in the following graphic. The table shows the number of entries on which the scores are based.

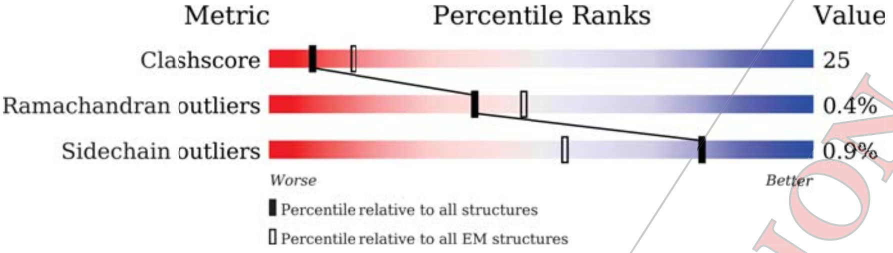

Metric	Whole archive (#Entries)	EM structures (#Entries)
Clashscore	158937	4297
Ramachandran outliers	154571	4023
Sidechain outliers	154315	3826

The table below summarises the geometric issues observed across the polymeric chains and their fit to the map. The red, orange, yellow and green segments on the bar indicate the fraction of residues that contain outliers for >=3, 2, 1 and 0 types of geometric quality criteria respectively. A grey segment represents the fraction of residues that are not modelled. The numeric value for each fraction is indicated below the corresponding segment, with a dot representing fractions <=5% The upper red bar (where present) indicates the fraction of residues that have poor fit to the EM map (all atom inclusion < 40%). The numeric value is given above the bar.

Mol	Chain	Length	Quality of chain
1	A	587	13% / 55% 43% ••
1	D	587	13% / 55% 42% ••
2	C	32	9% / 50% 47% •
2	F	32	9% / 50% 47% •
3	B	147	35% / 61% 39% •
3	E	147	35% / 61% 39% •

The following table lists non-polymeric compounds, carbohydrate monomers and non-standard residues in protein, DNA, RNA chains that are outliers for geometric or electron-density-fit criteria:

CONFIDENTIAL VALIDATION REPORT

Mol	Type	Chain	Res	Chirality	Geometry	Clashes	Electron density
8	PGV	A	607	-	-	X	-
8	PGV	D	601	-	-	X	-
9	3PE	A	606	-	-	X	-
9	3PE	D	607	-	-	X	-

CONFIDENTIAL VALIDATION REPORT

2 Entry composition (i)

There are 10 unique types of molecules in this entry. The entry contains 12852 atoms, of which 0 are hydrogens and 0 are deuteriums.

In the tables below, the AltConf column contains the number of residues with at least one atom in alternate conformation and the Trace column contains the number of residues modelled with at most 2 atoms.

- Molecule 1 is a protein called Cytochrome c oxidase subunit I.

Mol	Chain	Residues	Atoms					AltConf	Trace
1	A	581	Total 4624	C 3131	N 715	O 762	S 16	0	0
1	D	581	Total 4624	C 3131	N 715	O 762	S 16	0	0

- Molecule 2 is a protein called Cytochrome oxidase subunit IIa.

Mol	Chain	Residues	Atoms					AltConf	Trace
2	C	32	Total 281	C 202	N 37	O 39	S 3	0	0
2	F	32	Total 281	C 202	N 37	O 39	S 3	0	0

- Molecule 3 is a protein called Cytochrome oxidase subunit II.

Mol	Chain	Residues	Atoms					AltConf	Trace
3	B	147	Total 1159	C 758	N 195	O 199	S 7	0	0
3	E	147	Total 1159	C 758	N 195	O 199	S 7	0	0

- Molecule 4 is HEME-AS (three-letter code: HAS) (formula: $C_{54}H_{64}FeN_4O_6$).

CONFIDENTIAL VALIDATION REPORT

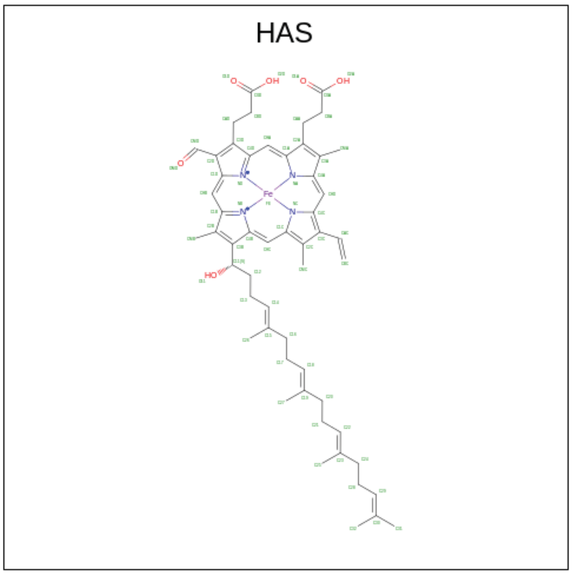

Mol	Chain	Residues	Atoms	AltConf
4	A	1	Total C Fe N O<br>65 54 1 4 6	0
4	D	1	Total C Fe N O<br>65 54 1 4 6	0

- Molecule 5 is PROTOPORPHYRIN IX CONTAINING FE (three-letter code: HEM) (formula: $C_{34}H_{32}FeN_4O_4$).

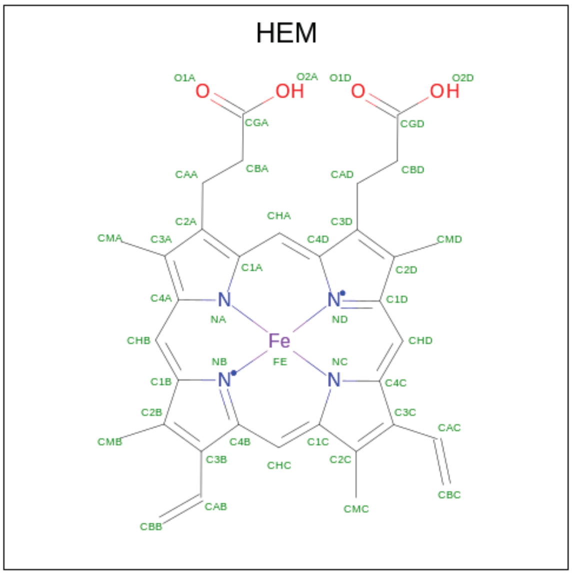

Mol	Chain	Residues	Atoms	AltConf
5	A	1	Total C Fe N O<br>43 34 1 4 4	0

Continued on next page...

VALIDATION REPORT

Continued from previous page...

Mol	Chain	Residues	Atoms	AltConf
5	D	1	Total C Fe N O<br>43 34 1 4 4	0

- Molecule 6 is COPPER (II) ION (three-letter code: CU) (formula: Cu).

Mol	Chain	Residues	Atoms	AltConf
6	A	1	Total Cu<br>1 1	0
6	D	1	Total Cu<br>1 1	0

- Molecule 7 is 2-[(2 {E},6 {E},10 {Z},14 {Z},18 {Z},23 {R})-3,7,11,15,19,23,27-heptamethyloctacosa-2,6,10,14,18-pentaenyl]naphthalene-1,4-dione (three-letter code: DLX) (formula: $C_{45}H_{66}O_2$).

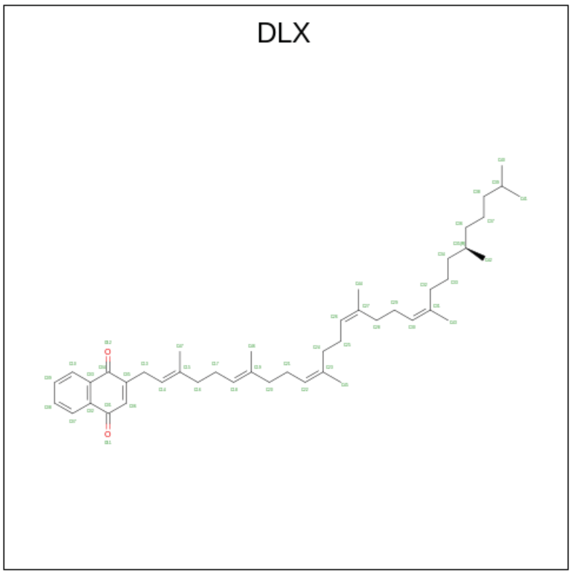

Mol	Chain	Residues	Atoms	AltConf
7	A	1	Total C O<br>47 45 2	0
7	D	1	Total C O<br>47 45 2	0

- Molecule 8 is (1R)-2-{[{[(2S)-2,3-DIHYDROXYPROPYL]OXY}(HYDROXY)PHOSPHORYL]OXY}-1-[(PALMITOYLOXY)METHYL]ETHYL (11E)-OCTADEC-11-ENOATE (three-letter code: PGV) (formula: $C_{40}H_{77}O_{10}P$).

CONFIDENTIAL VALIDATION REPORT

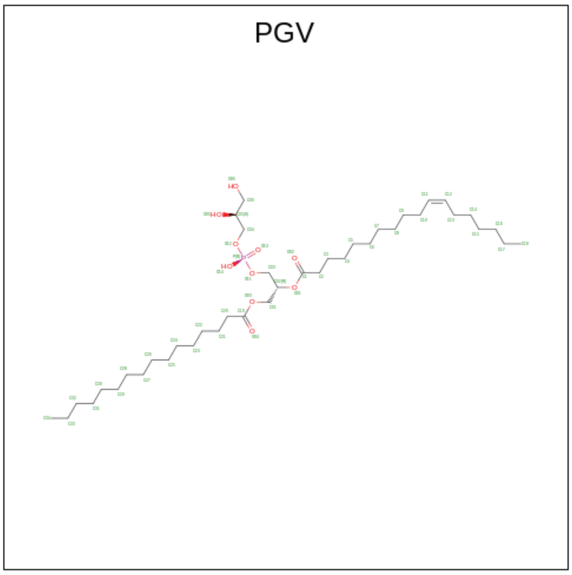

Mol	Chain	Residues	Atoms	AltConf
8	A	1	Total C O P<br>102 80 20 2	0
8	A	1	Total C O P<br>102 80 20 2	0
8	C	1	Total C O P<br>51 40 10 1	0
8	D	1	Total C O P<br>102 80 20 2	0
8	D	1	Total C O P<br>102 80 20 2	0
8	F	1	Total C O P<br>51 40 10 1	0

- Molecule 9 is 1,2-DIACYL-SN-GLYCERO-3-PHOSPHOETHANOLAMINE (three-letter code: 3PE) (formula: $C_{41}H_{82}NO_8P$).

CONFIDENTIAL VALIDATION REPORT

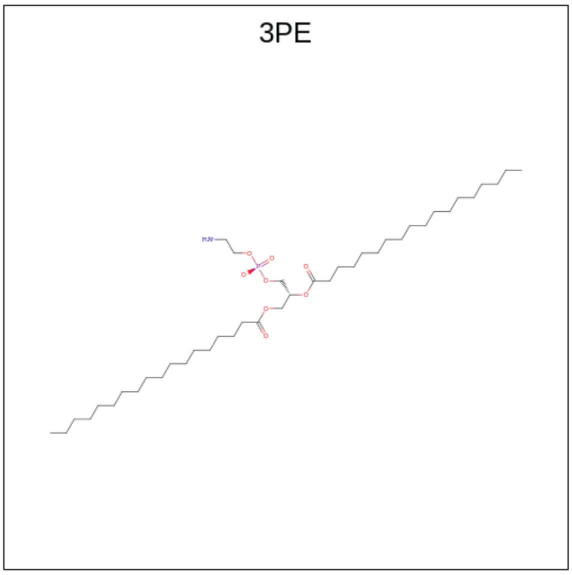

Mol	Chain	Residues	Atoms	AltConf
9	A	1	Total C N O P<br>51 41 1 8 1	0
9	D	1	Total C N O P<br>51 41 1 8 1	0

- Molecule 10 is DINUCLEAR COPPER ION (three-letter code: CUA) (formula: Cu_2).

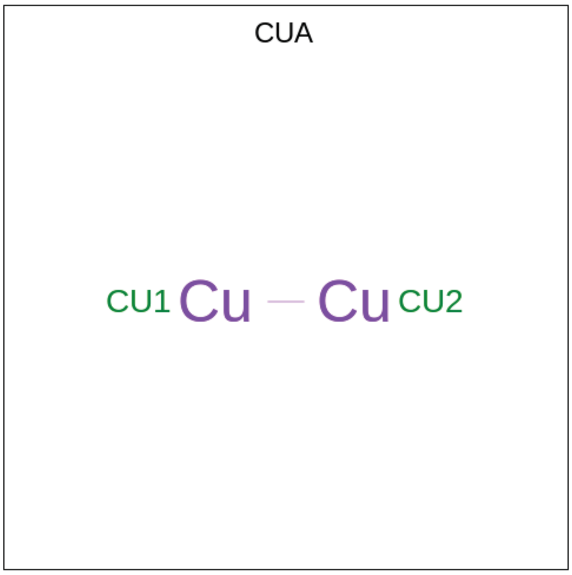

Mol	Chain	Residues	Atoms	AltConf
10	B	1	Total Cu<br>2 2	0
10	E	1	Total Cu<br>2 2	0

CONFIDENTIAL VALIDATION REPORT

3 Residue-property plots

These plots are drawn for all protein, RNA, DNA and oligosaccharide chains in the entry. The first graphic for a chain summarises the proportions of the various outlier classes displayed in the second graphic. The second graphic shows the sequence view annotated by issues in geometry and atom inclusion in map density. Residues are color-coded according to the number of geometric quality criteria for which they contain at least one outlier: green = 0, yellow = 1, orange = 2 and red = 3 or more. A red diamond above a residue indicates a poor fit to the EM map for this residue (all atom inclusion < 40%). Stretches of 2 or more consecutive residues without any outlier are shown as a green connector. Residues present in the sample, but not in the model, are shown in grey.

- Molecule 1: Cytochrome c oxidase subunit I

Chain A: 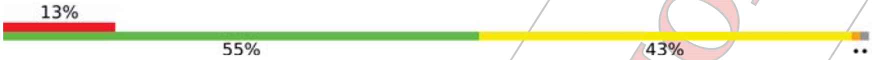

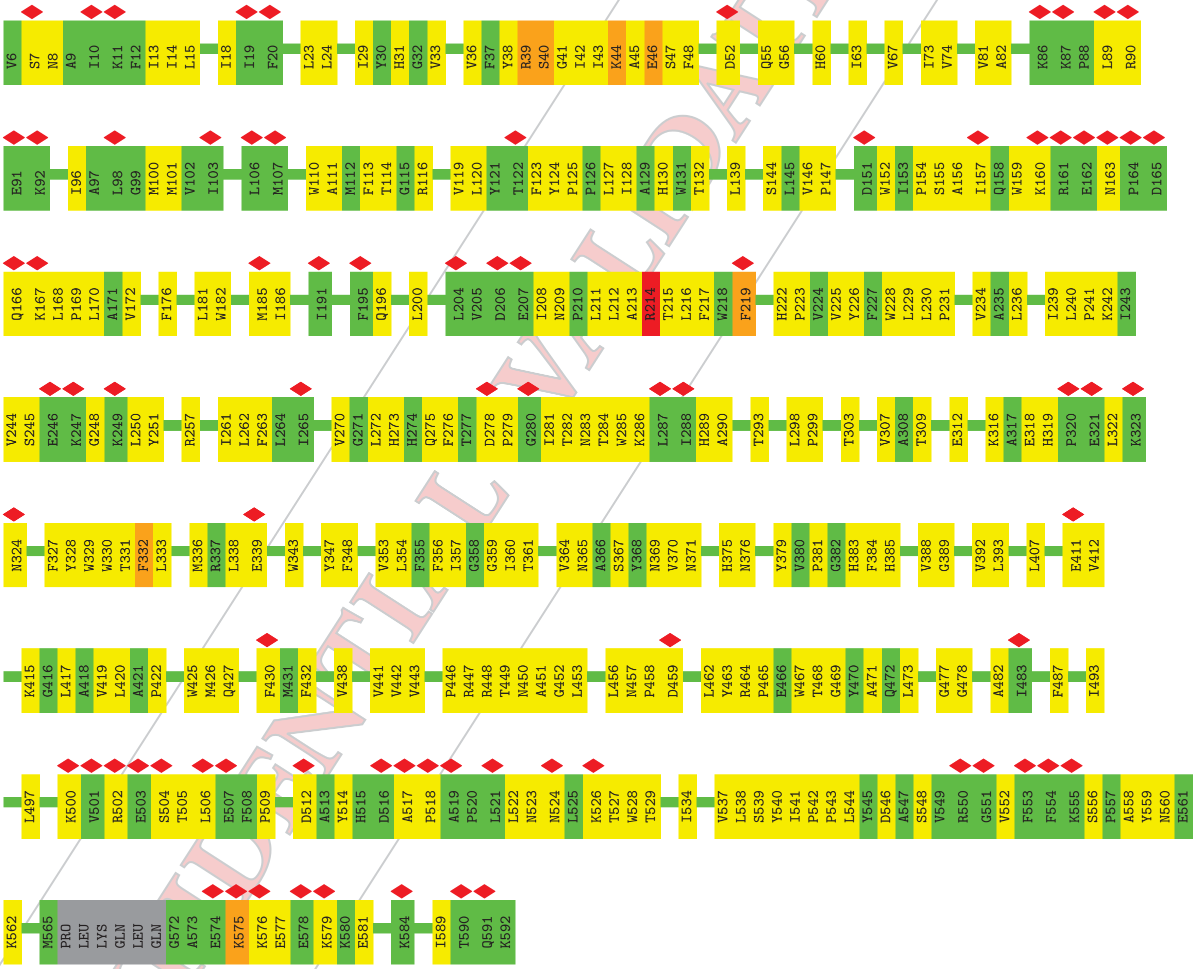

- Molecule 1: Cytochrome c oxidase subunit I

Chain D: 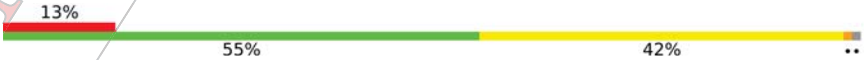

CONFIDENTIAL VALIDATION REPORT

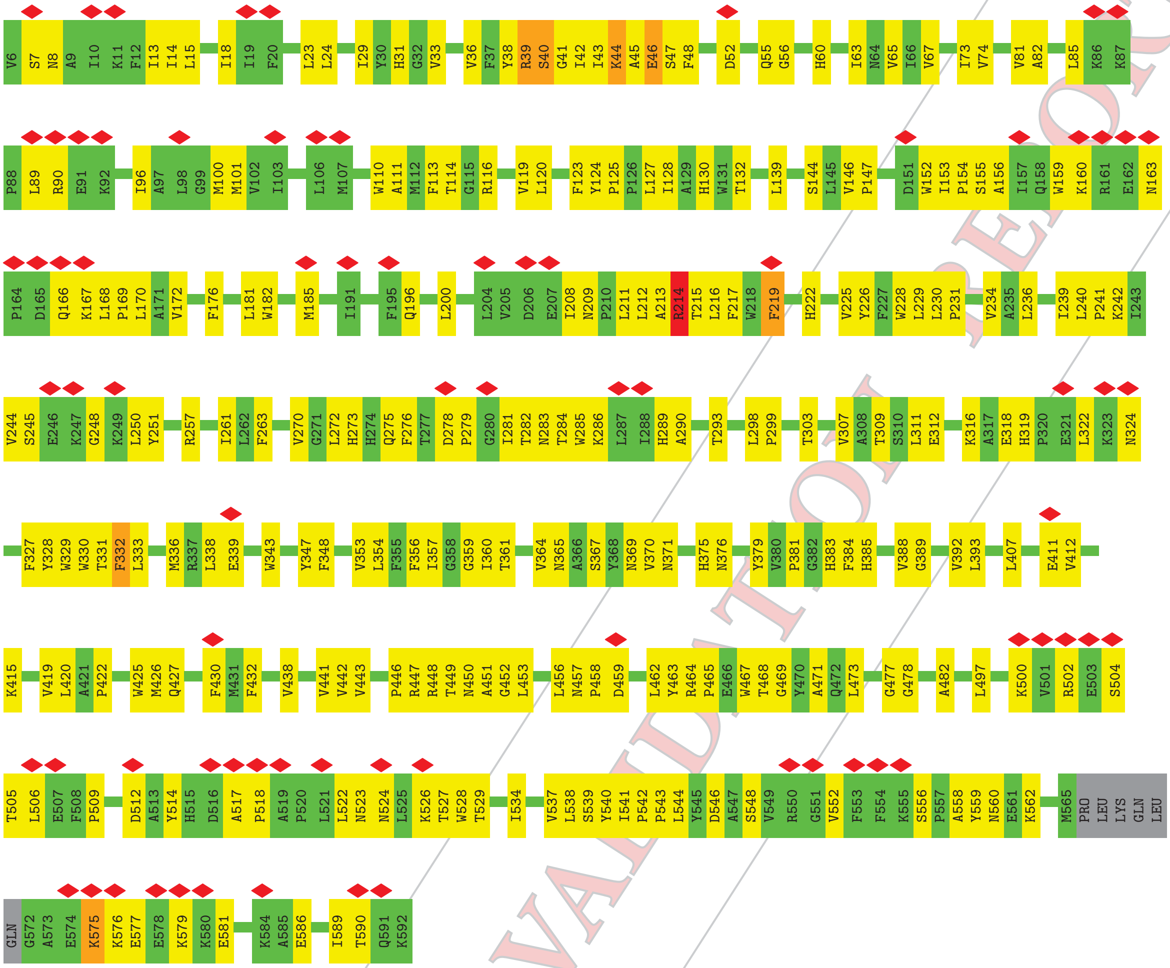

• Molecule 2: Cytochrome oxidase subunit IIa

Chain C:

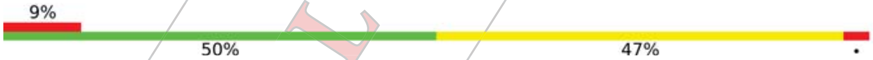

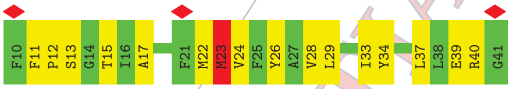

• Molecule 2: Cytochrome oxidase subunit IIa

Chain F:

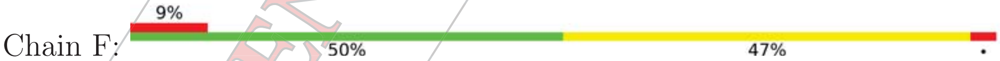

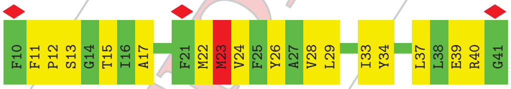

• Molecule 3: Cytochrome oxidase subunit II

Chain B:

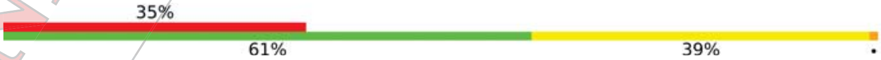

CONFIDENTIAL VALIDATION REPORT

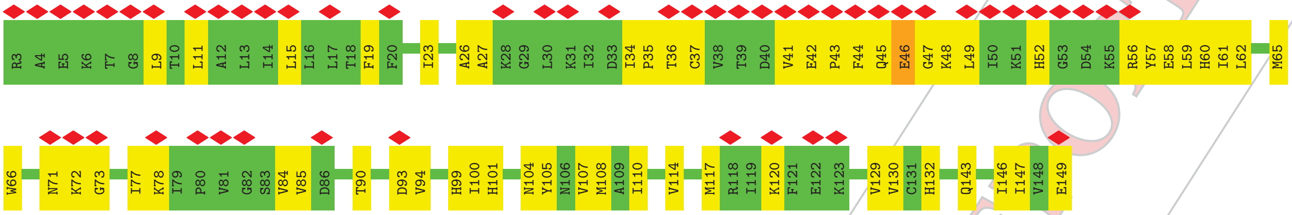

• Molecule 3: Cytochrome oxidase subunit II

Chain E:

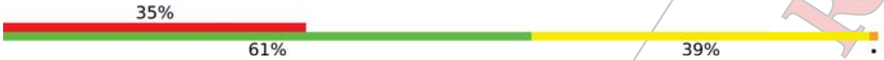

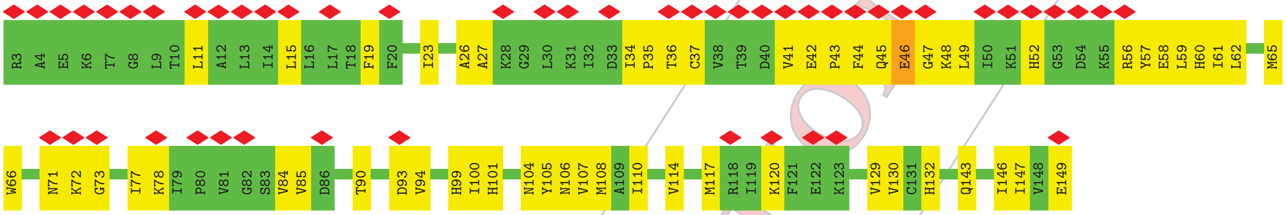

CONFIDENTIAL VALIDATION REPORT

4 Experimental information (i)

Property	Value	Source
EM reconstruction method	SINGLE PARTICLE	Depositor
Imposed symmetry	POINT, Not provided	Depositor
Number of particles used	32982	Depositor
Resolution determination method	FSC 0.143 CUT-OFF	Depositor
CTF correction method	NONE	Depositor
Microscope	FEI TITAN KRIOS	Depositor
Voltage (kV)	300	Depositor
Electron dose ($e^-/\text{Å}^2$)	60	Depositor
Minimum defocus (nm)	Not provided	Depositor
Maximum defocus (nm)	Not provided	Depositor
Magnification	Not provided	Depositor
Image detector	GATAN K2 SUMMIT (4k x 4k)	Depositor
Maximum map value	0.077	Depositor
Minimum map value	-0.051	Depositor
Average map value	0.000	Depositor
Map value standard deviation	0.002	Depositor
Recommended contour level	0.02	Depositor
Map size (Å)	266.24, 266.24, 266.24	Depositor
Map dimensions	256, 256, 256	Depositor
Map angles (°)	90.0, 90.0, 90.0	Depositor
Pixel spacing (Å)	1.04, 1.04, 1.04	Depositor

CONFIDENTIAL VALIDATION REPORT

5 Model quality (i)

5.1 Standard geometry (i)

Bond lengths and bond angles in the following residue types are not validated in this section: DLX, PGV, CUA, HEM, HAS, 3PE, CU

The Z score for a bond length (or angle) is the number of standard deviations the observed value is removed from the expected value. A bond length (or angle) with $|Z| > 5$ is considered an outlier worth inspection. RMSZ is the root-mean-square of all Z scores of the bond lengths (or angles).

Mol	Chain	Bond lengths		Bond angles	
		RMSZ	#$|Z|$ >5	RMSZ	#$|Z|$ >5
1	A	0.81	1/4777 (0.0%)	0.65	0/6521
1	D	0.81	1/4777 (0.0%)	0.65	0/6521
2	C	0.74	0/294	0.60	0/397
2	F	0.74	0/294	0.60	0/397
3	B	0.72	0/1188	0.63	0/1607
3	E	0.72	0/1188	0.63	0/1607
All	All	0.79	2/12518 (0.0%)	0.65	0/17050

Chiral center outliers are detected by calculating the chiral volume of a chiral center and verifying if the center is modelled as a planar moiety or with the opposite hand.A planarity outlier is detected by checking planarity of atoms in a peptide group, atoms in a mainchain group or atoms of a sidechain that are expected to be planar.

Mol	Chain	#Chirality outliers	#Planarity outliers
1	A	0	2
1	D	0	2
2	C	0	1
2	F	0	1
3	B	0	1
3	E	0	1
All	All	0	8

All (2) bond length outliers are listed below:

Mol	Chain	Res	Type	Atoms	Z	Observed(Å)	Ideal(Å)
1	D	332	PHE	C-N	-5.09	1.22	1.34
1	A	332	PHE	C-N	-5.08	1.22	1.34

There are no bond angle outliers.

There are no chirality outliers.

CONFIDENTIAL VALIDATION REPORT

All (8) planarity outliers are listed below:

Mol	Chain	Res	Type	Group
1	A	214	ARG	Peptide
1	A	512	ASP	Peptide
3	B	46	GLU	Peptide
2	C	23	MET	Peptide
1	D	214	ARG	Peptide
1	D	512	ASP	Peptide
3	E	46	GLU	Peptide
2	F	23	MET	Peptide

5.2 Too-close contacts ⓘ

In the following table, the Non-H and H(model) columns list the number of non-hydrogen atoms and hydrogen atoms in the chain respectively. The H(added) column lists the number of hydrogen atoms added and optimized by MolProbity. The Clashes column lists the number of clashes within the asymmetric unit, whereas Symm-Clashes lists symmetry related clashes.

Mol	Chain	Non-H	H(model)	H(added)	Clashes	Symm-Clashes
1	A	4624	0	4725	238	0
1	D	4624	0	4725	238	0
2	C	281	0	274	24	0
2	F	281	0	274	23	0
3	B	1159	0	1184	54	0
3	E	1159	0	1184	53	0
4	A	65	0	62	11	0
4	D	65	0	62	11	0
5	A	43	0	30	8	0
5	D	43	0	30	8	0
6	A	1	0	0	0	0
6	D	1	0	0	0	0
7	A	47	0	0	3	0
7	D	47	0	0	3	0
8	A	102	0	151	30	0
8	C	51	0	76	4	0
8	D	102	0	151	29	0
8	F	51	0	76	4	0
9	A	51	0	80	36	0
9	D	51	0	80	34	0
10	B	2	0	0	0	0
10	E	2	0	0	0	0
All	All	12852	0	13164	653	0

CONFIDENTIAL VALIDATION REPORT

The all-atom clashscore is defined as the number of clashes found per 1000 atoms (including hydrogen atoms). The all-atom clashscore for this structure is 25.

All (653) close contacts within the same asymmetric unit are listed below, sorted by their clash magnitude.

Atom-1	Atom-2	Interatomic distance (Å)	Clash overlap (Å)
1:D:327:PHE:O	9:D:607:3PE:C22	1.68	1.41
1:A:327:PHE:O	9:A:606:3PE:C22	1.68	1.39
1:D:40:SER:HB2	1:D:471:ALA:O	1.31	1.27
1:A:40:SER:HB2	1:A:471:ALA:O	1.31	1.22
3:B:48:LYS:HD2	3:B:77:ILE:HD11	1.48	0.96
3:E:48:LYS:HD2	3:E:77:ILE:HD11	1.48	0.93
1:A:446:PRO:HG2	1:A:449:THR:HG21	1.57	0.87
1:A:40:SER:CB	1:A:471:ALA:O	2.22	0.86
1:D:446:PRO:HG2	1:D:449:THR:HG21	1.57	0.86
1:D:40:SER:CB	1:D:471:ALA:O	2.22	0.85
3:E:52:HIS:HB2	3:E:56:ARG:HB2	1.60	0.83
9:A:606:3PE:H272	1:D:336:MET:CE	2.09	0.82
1:A:336:MET:CE	9:D:607:3PE:H272	2.09	0.82
3:B:52:HIS:HB2	3:B:56:ARG:HB2	1.60	0.82
8:A:607:PGV:H212	8:D:601:PGV:H212	1.62	0.81
3:E:42:GLU:HB3	3:E:114:VAL:HG21	1.63	0.81
3:B:42:GLU:HB3	3:B:114:VAL:HG21	1.63	0.80
1:A:427:GLN:HB2	8:A:607:PGV:H231	1.63	0.80
1:A:119:VAL:HG13	1:A:120:LEU:H	1.47	0.79
1:D:43:ILE:O	1:D:44:LYS:O	2.01	0.79
1:A:312:GLU:OE2	1:A:316:LYS:NZ	2.15	0.79
1:D:119:VAL:HG13	1:D:120:LEU:H	1.47	0.78
1:D:427:GLN:HB2	8:D:601:PGV:H231	1.65	0.78
1:D:312:GLU:OE2	1:D:316:LYS:NZ	2.15	0.78
1:D:168:LEU:O	1:D:257:ARG:NH2	2.17	0.77
1:A:464:ARG:NH2	3:B:143:GLN:OE1	2.18	0.77
8:D:601:PGV:C4	9:D:607:3PE:H2A1	2.15	0.77
1:A:43:ILE:O	1:A:44:LYS:O	2.01	0.76
1:D:464:ARG:NH2	3:E:143:GLN:OE1	2.18	0.76
9:A:606:3PE:H2A1	8:A:607:PGV:C4	2.15	0.76
9:D:607:3PE:H3B1	8:F:101:PGV:H201	1.66	0.76
9:A:606:3PE:H3B1	8:C:101:PGV:H201	1.66	0.76
8:A:607:PGV:H312	9:D:607:3PE:H2B1	1.68	0.75
9:A:606:3PE:H2B1	8:D:601:PGV:H312	1.68	0.75
1:A:365:ASN:O	1:A:371:ASN:ND2	2.20	0.75
3:B:65:MET:HB2	3:B:93:ASP:OD2	1.86	0.75
3:E:65:MET:HB2	3:E:93:ASP:OD2	1.86	0.75

Continued on next page...

CONFIDENTIAL VALIDATION REPORT

Continued from previous page...

Atom-1	Atom-2	Interatomic distance (Å)	Clash overlap (Å)
1:D:529:THR:HG21	1:D:589:ILE:HG21	1.69	0.75
1:D:39:ARG:HG2	1:D:39:ARG:HH11	1.52	0.74
1:A:144:SER:HB2	1:A:182:TRP:CH2	2.22	0.74
1:A:529:THR:HG21	1:A:589:ILE:HG21	1.69	0.74
1:D:144:SER:HB2	1:D:182:TRP:CH2	2.22	0.74
1:D:36:VAL:HG11	1:D:478:GLY:HA3	1.70	0.74
1:A:39:ARG:HH11	1:A:39:ARG:HG2	1.52	0.74
1:D:73:ILE:HD12	1:D:231:PRO:HG2	1.70	0.73
1:D:411:GLU:HG2	1:D:412:VAL:H	1.54	0.73
1:D:39:ARG:HG2	1:D:39:ARG:NH1	2.03	0.73
1:A:360:ILE:HB	2:C:26:TYR:CD1	2.24	0.73
1:A:524:ASN:ND2	1:A:527:THR:OG1	2.22	0.73
1:D:360:ILE:HB	2:F:26:TYR:CD1	2.24	0.73
1:A:360:ILE:HG21	2:C:29:LEU:HD12	1.70	0.73
1:D:360:ILE:HG21	2:F:29:LEU:HD12	1.70	0.73
1:A:36:VAL:HG11	1:A:478:GLY:HA3	1.70	0.72
9:A:606:3PE:H3A1	8:C:101:PGV:H92	1.71	0.72
1:A:168:LEU:O	1:A:257:ARG:NH2	2.17	0.72
1:A:411:GLU:HG2	1:A:412:VAL:H	1.54	0.72
2:F:23:MET:HA	2:F:26:TYR:HB2	1.71	0.72
2:C:23:MET:HA	2:C:26:TYR:HB2	1.71	0.72
1:A:73:ILE:HD12	1:A:231:PRO:HG2	1.70	0.72
1:D:160:LYS:NZ	1:D:166:GLN:O	2.23	0.72
1:D:365:ASN:O	1:D:371:ASN:ND2	2.20	0.72
1:A:465:PRO:HB2	1:D:465:PRO:HB2	1.71	0.72
9:A:606:3PE:H292	8:D:601:PGV:C31	2.20	0.71
9:D:607:3PE:H3A1	8:F:101:PGV:H92	1.71	0.71
1:A:327:PHE:HB2	9:A:606:3PE:H2	1.73	0.71
1:D:524:ASN:ND2	1:D:527:THR:OG1	2.22	0.71
1:D:81:VAL:HG22	1:D:172:VAL:HG23	1.73	0.71
1:A:160:LYS:NZ	1:A:166:GLN:O	2.23	0.71
1:A:81:VAL:HG22	1:A:172:VAL:HG23	1.73	0.70
1:D:146:VAL:HG13	1:D:147:PRO:HD3	1.72	0.70
1:A:181:LEU:O	1:A:185:MET:HG3	1.91	0.70
8:A:607:PGV:C31	9:D:607:3PE:H292	2.20	0.70
9:A:606:3PE:H272	1:D:336:MET:HE1	1.74	0.70
1:A:544:LEU:HB3	1:A:575:LYS:HG3	1.74	0.70
8:A:607:PGV:H312	9:D:607:3PE:C2A	2.22	0.70
1:A:146:VAL:HG13	1:A:147:PRO:HD3	1.72	0.69
1:D:544:LEU:HB3	1:D:575:LYS:HG3	1.74	0.69
1:A:367:SER:O	1:A:370:VAL:N	2.25	0.69

Continued on next page...

CONFIDENTIAL VALIDATION REPORT

Continued from previous page...

Atom-1	Atom-2	Interatomic distance (Å)	Clash overlap (Å)
3:E:100:ILE:HG12	3:E:129:VAL:HG22	1.75	0.69
1:D:181:LEU:O	1:D:185:MET:HG3	1.91	0.69
1:A:39:ARG:NH1	1:A:39:ARG:HG2	2.03	0.69
1:D:327:PHE:HB2	9:D:607:3PE:H2	1.73	0.69
9:A:606:3PE:C2A	8:D:601:PGV:H312	2.22	0.69
8:A:607:PGV:H302	9:D:607:3PE:H292	1.74	0.69
1:A:360:ILE:HB	2:C:26:TYR:CE1	2.28	0.69
3:B:100:ILE:HG12	3:B:129:VAL:HG22	1.75	0.69
1:D:367:SER:O	1:D:370:VAL:N	2.25	0.69
1:D:222:HIS:CD2	1:D:226:TYR:HE1	2.11	0.69
1:D:442:VAL:HG23	1:D:443:VAL:HG13	1.74	0.69
9:A:606:3PE:H292	8:D:601:PGV:H302	1.74	0.68
1:A:385:HIS:NE2	5:A:602:HEM:ND	2.42	0.68
1:A:222:HIS:CD2	1:A:226:TYR:HE1	2.11	0.68
1:A:442:VAL:HG23	1:A:443:VAL:HG13	1.74	0.68
3:E:45:GLN:HA	3:E:62:LEU:HD13	1.75	0.68
8:A:607:PGV:H312	9:D:607:3PE:C2B	2.24	0.68
1:D:462:LEU:HD22	3:E:143:GLN:HG2	1.76	0.68
1:D:90:ARG:HH12	1:D:155:SER:HB3	1.59	0.68
1:D:360:ILE:HB	2:F:26:TYR:CE1	2.28	0.67
1:D:385:HIS:NE2	5:D:603:HEM:ND	2.42	0.67
3:B:45:GLN:HA	3:B:62:LEU:HD13	1.75	0.67
1:D:231:PRO:HA	1:D:234:VAL:HG12	1.76	0.67
2:F:23:MET:HB2	3:E:15:LEU:HD22	1.77	0.67
1:A:128:ILE:HG12	1:A:558:ALA:HB1	1.77	0.67
1:A:336:MET:HE1	9:D:607:3PE:H272	1.77	0.67
1:A:276:PHE:HB3	3:B:34:ILE:HD11	1.77	0.67
1:A:125:PRO:HA	1:A:127:LEU:H	1.60	0.67
1:A:239:ILE:HG23	1:A:509:PRO:HG2	1.77	0.67
2:C:23:MET:HB2	3:B:15:LEU:HD22	1.77	0.67
1:D:128:ILE:HG12	1:D:558:ALA:HB1	1.77	0.67
1:A:449:THR:HG23	1:A:451:ALA:HB2	1.78	0.66
1:A:462:LEU:HD22	3:B:143:GLN:HG2	1.76	0.66
1:D:276:PHE:HB3	3:E:34:ILE:HD11	1.77	0.66
9:A:606:3PE:C2B	8:D:601:PGV:H312	2.24	0.66
1:A:231:PRO:HA	1:A:234:VAL:HG12	1.76	0.66
1:A:367:SER:HB3	3:B:23:ILE:HG21	1.77	0.66
1:D:367:SER:HB3	3:E:23:ILE:HG21	1.77	0.66
1:D:239:ILE:HG23	1:D:509:PRO:HG2	1.77	0.65
4:D:602:HAS:HBC1	4:D:602:HAS:HMC1	1.78	0.65
3:E:41:VAL:HG22	3:E:43:PRO:HD3	1.78	0.65

Continued on next page...

CONFIDENTIAL VALIDATION REPORT

Continued from previous page...

Atom-1	Atom-2	Interatomic distance (Å)	Clash overlap (Å)
1:D:449:THR:HG23	1:D:451:ALA:HB2	1.77	0.65
1:D:125:PRO:HA	1:D:127:LEU:H	1.60	0.65
3:E:47:GLY:HA3	3:E:60:HIS:HB2	1.78	0.65
1:A:90:ARG:HH12	1:A:155:SER:HB3	1.59	0.65
8:A:607:PGV:H02	8:D:601:PGV:H02	1.79	0.65
1:D:364:VAL:HG11	2:F:33:ILE:HD13	1.79	0.65
4:A:601:HAS:HBC1	4:A:601:HAS:HMC1	1.78	0.64
1:A:63:ILE:HA	1:A:67:VAL:HG22	1.80	0.64
1:D:55:GLN:NE2	1:D:113:PHE:O	2.30	0.64
3:B:47:GLY:HA3	3:B:60:HIS:HB2	1.78	0.64
1:D:343:TRP:HB2	1:D:419:VAL:HG22	1.78	0.64
3:B:41:VAL:HG22	3:B:43:PRO:HD3	1.78	0.64
1:D:338:LEU:O	1:D:339:GLU:HG2	1.97	0.64
1:A:343:TRP:HB2	1:A:419:VAL:HG22	1.78	0.64
1:A:55:GLN:NE2	1:A:113:PHE:O	2.30	0.64
3:B:71:ASN:O	3:B:73:GLY:N	2.31	0.64
1:A:364:VAL:HG11	2:C:33:ILE:HD13	1.79	0.64
3:E:56:ARG:HD2	3:E:84:VAL:HG13	1.80	0.63
1:A:338:LEU:O	1:A:339:GLU:HG2	1.97	0.63
1:D:63:ILE:HA	1:D:67:VAL:HG22	1.80	0.63
3:E:71:ASN:O	3:E:73:GLY:N	2.31	0.63
3:B:56:ARG:HD2	3:B:84:VAL:HG13	1.80	0.63
1:A:40:SER:HA	1:A:471:ALA:HB1	1.81	0.62
9:A:606:3PE:H2A1	8:A:607:PGV:H42	1.81	0.62
1:D:45:ALA:O	1:D:453:LEU:HD22	2.00	0.62
1:D:524:ASN:ND2	1:D:526:LYS:O	2.33	0.62
1:A:524:ASN:ND2	1:A:526:LYS:O	2.33	0.62
1:A:330:TRP:HA	1:A:333:LEU:HD12	1.82	0.62
1:A:45:ALA:O	1:A:453:LEU:HD22	2.00	0.62
1:D:33:VAL:HG21	1:D:482:ALA:HB2	1.81	0.62
9:A:606:3PE:C3A	8:C:101:PGV:H92	2.30	0.62
1:D:330:TRP:HA	1:D:333:LEU:HD12	1.82	0.62
1:D:40:SER:HA	1:D:471:ALA:HB1	1.81	0.62
9:A:606:3PE:H292	8:D:601:PGV:C30	2.29	0.62
2:F:22:MET:O	2:F:24:VAL:N	2.33	0.62
8:D:601:PGV:H41	9:D:607:3PE:H291	1.82	0.61
1:A:361:THR:HG23	1:A:379:TYR:CE2	2.35	0.61
9:A:606:3PE:H2B1	8:D:601:PGV:H331	1.82	0.61
1:D:46:GLU:HG2	1:D:46:GLU:O	2.00	0.61
8:A:607:PGV:C30	9:D:607:3PE:H292	2.29	0.61
1:A:46:GLU:O	1:A:46:GLU:HG2	2.00	0.61

Continued on next page...

CONFIDENTIAL VALIDATION REPORT

Continued from previous page...

Atom-1	Atom-2	Interatomic distance (Å)	Clash overlap (Å)
1:D:214:ARG:NH2	1:D:278:ASP:OD1	2.28	0.61
2:C:22:MET:O	2:C:24:VAL:N	2.33	0.61
1:A:214:ARG:NH2	1:A:278:ASP:OD1	2.28	0.61
1:A:407:LEU:O	1:A:504:SER:OG	2.18	0.61
1:A:336:MET:SD	9:D:607:3PE:H272	2.41	0.61
1:D:361:THR:HG23	1:D:379:TYR:CE2	2.35	0.61
1:D:411:GLU:OE1	1:D:411:GLU:N	2.19	0.61
1:A:33:VAL:HG21	1:A:482:ALA:HB2	1.81	0.61
8:D:601:PGV:H42	9:D:607:3PE:H2A1	1.81	0.61
9:D:607:3PE:C3A	8:F:101:PGV:H92	2.31	0.60
1:A:125:PRO:HG3	1:A:213:ALA:HB3	1.83	0.60
8:A:607:PGV:H331	9:D:607:3PE:H2B1	1.82	0.60
1:A:411:GLU:N	1:A:411:GLU:OE1	2.19	0.60
5:A:602:HEM:HHC	5:A:602:HEM:HBB2	1.82	0.60
1:A:7:SER:OG	1:A:8:ASN:N	2.34	0.60
5:D:603:HEM:HBB2	5:D:603:HEM:HHC	1.82	0.60
1:D:500:LYS:NZ	1:D:502:ARG:O	2.34	0.60
1:D:7:SER:OG	1:D:8:ASN:N	2.34	0.60
3:B:93:ASP:OD1	3:B:94:VAL:N	2.35	0.60
1:A:120:LEU:HD23	1:A:448:ARG:CZ	2.32	0.60
9:A:606:3PE:H272	1:D:336:MET:SD	2.41	0.60
1:D:39:ARG:CG	1:D:39:ARG:HH11	2.14	0.60
1:A:415:LYS:O	1:A:419:VAL:HG23	2.02	0.60
1:D:415:LYS:O	1:D:419:VAL:HG23	2.02	0.60
1:A:500:LYS:NZ	1:A:502:ARG:O	2.34	0.59
9:A:606:3PE:H291	8:A:607:PGV:H41	1.83	0.59
1:A:279:PRO:HD2	3:B:110:ILE:HD12	1.83	0.59
1:D:120:LEU:HD23	1:D:448:ARG:CZ	2.32	0.59
1:D:125:PRO:HG3	1:D:213:ALA:HB3	1.83	0.59
1:D:329:TRP:O	1:D:330:TRP:HB2	2.02	0.59
1:D:560:ASN:HD22	1:D:562:LYS:H	1.50	0.59
8:A:605:PGV:H71	8:A:605:PGV:H21	1.85	0.59
8:A:607:PGV:H312	9:D:607:3PE:H292	1.84	0.59
1:D:124:TYR:OH	1:D:214:ARG:NH1	2.36	0.59
3:E:77:ILE:HG22	3:E:146:ILE:HA	1.84	0.59
1:A:329:TRP:O	1:A:330:TRP:HB2	2.02	0.58
1:A:124:TYR:OH	1:A:214:ARG:NH1	2.36	0.58
1:A:196:GLN:HE22	1:A:212:LEU:HD23	1.69	0.58
1:D:279:PRO:HD2	3:E:110:ILE:HD12	1.83	0.58
9:A:606:3PE:H292	8:D:601:PGV:H312	1.84	0.58
8:D:606:PGV:H71	8:D:606:PGV:H21	1.85	0.58

Continued on next page...

CONFIDENTIAL VALIDATION REPORT

Continued from previous page...

Atom-1	Atom-2	Interatomic distance (Å)	Clash overlap (Å)
1:D:407:LEU:O	1:D:504:SER:OG	2.18	0.58
1:D:196:GLN:HE22	1:D:212:LEU:HD23	1.69	0.58
3:E:93:ASP:OD1	3:E:94:VAL:N	2.35	0.58
3:B:56:ARG:HD2	3:B:84:VAL:CG1	2.34	0.58
1:A:473:LEU:HD12	1:D:473:LEU:HD12	1.85	0.58
3:E:41:VAL:HG13	3:E:42:GLU:H	1.69	0.57
1:A:39:ARG:CG	1:A:39:ARG:HH11	2.14	0.57
9:A:606:3PE:H2B2	9:A:606:3PE:C26	2.35	0.57
3:B:77:ILE:HG22	3:B:146:ILE:HA	1.84	0.57
1:D:283:ASN:OD1	1:D:284:THR:N	2.36	0.57
9:A:606:3PE:C29	8:D:601:PGV:H312	2.34	0.57
1:A:14:ILE:O	1:A:18:ILE:HG12	2.04	0.57
1:A:560:ASN:HD22	1:A:562:LYS:H	1.50	0.57
9:D:607:3PE:H2B2	9:D:607:3PE:C26	2.34	0.57
1:A:152:TRP:HD1	1:A:176:PHE:HE1	1.53	0.57
1:D:14:ILE:O	1:D:18:ILE:HG12	2.04	0.57
8:A:607:PGV:H312	9:D:607:3PE:C29	2.34	0.57
1:D:152:TRP:HD1	1:D:176:PHE:HE1	1.53	0.57
3:E:58:GLU:OE2	3:E:60:HIS:NE2	2.37	0.57
1:D:353:VAL:HA	4:D:602:HAS:H323	1.86	0.57
3:B:58:GLU:OE2	3:B:60:HIS:NE2	2.37	0.57
1:D:389:GLY:O	1:D:425:TRP:NE1	2.38	0.57
1:A:353:VAL:HA	4:A:601:HAS:H323	1.86	0.56
1:D:38:TYR:OH	1:D:48:PHE:HB2	2.05	0.56
3:E:56:ARG:HD2	3:E:84:VAL:CG1	2.34	0.56
3:B:41:VAL:HG13	3:B:42:GLU:H	1.69	0.56
1:D:456:LEU:HD22	1:D:468:THR:HG22	1.88	0.56
3:E:46:GLU:OE1	3:E:72:LYS:HD3	2.06	0.56
1:D:537:VAL:HG13	1:D:579:LYS:HD2	1.87	0.56
1:A:241:PRO:O	1:A:248:GLY:HA3	2.06	0.56
1:A:328:TYR:HB2	9:A:606:3PE:O12	2.06	0.56
1:A:251:TYR:HA	1:A:514:TYR:HB3	1.87	0.56
1:A:283:ASN:OD1	1:A:284:THR:N	2.36	0.55
1:D:241:PRO:O	1:D:248:GLY:HA3	2.06	0.55
1:D:328:TYR:HB2	9:D:607:3PE:O12	2.06	0.55
1:D:47:SER:HA	1:D:52:ASP:HA	1.88	0.55
1:A:244:VAL:HG12	1:A:245:SER:H	1.71	0.55
1:A:38:TYR:OH	1:A:48:PHE:HB2	2.05	0.55
1:D:15:LEU:HG	1:D:407:LEU:HD11	1.89	0.55
8:D:601:PGV:H52	9:D:607:3PE:H2A2	1.89	0.55
1:A:215:THR:C	1:A:217:PHE:H	2.10	0.55

Continued on next page...

CONFIDENTIAL VALIDATION REPORT

Continued from previous page...

Atom-1	Atom-2	Interatomic distance (Å)	Clash overlap (Å)
3:B:46:GLU:OE1	3:B:72:LYS:HD3	2.06	0.55
1:D:359:GLY:HA3	4:D:602:HAS:H161	1.89	0.55
1:A:359:GLY:HA3	4:A:601:HAS:H161	1.89	0.55
3:B:36:THR:OG1	3:B:37:CYS:N	2.40	0.55
1:D:244:VAL:HG12	1:D:245:SER:H	1.71	0.55
1:D:251:TYR:HA	1:D:514:TYR:HB3	1.87	0.55
1:A:456:LEU:HD22	1:A:468:THR:HG22	1.88	0.55
1:A:15:LEU:HG	1:A:407:LEU:HD11	1.89	0.55
1:D:450:ASN:O	1:D:452:GLY:N	2.40	0.55
1:A:43:ILE:O	1:A:44:LYS:C	2.45	0.55
1:A:450:ASN:O	1:A:452:GLY:N	2.40	0.55
3:E:36:THR:OG1	3:E:37:CYS:N	2.40	0.55
1:D:272:LEU:HD21	1:D:281:ILE:HG21	1.88	0.54
1:D:215:THR:C	1:D:217:PHE:H	2.10	0.54
1:A:537:VAL:HG13	1:A:579:LYS:HD2	1.87	0.54
1:D:119:VAL:HG22	1:D:123:PHE:HA	1.89	0.54
1:D:116:ARG:HB3	1:D:130:HIS:HB2	1.90	0.54
1:A:119:VAL:HG22	1:A:123:PHE:HA	1.90	0.54
1:D:214:ARG:NE	1:D:278:ASP:OD2	2.41	0.54
1:A:116:ARG:HB3	1:A:130:HIS:HB2	1.90	0.54
1:A:272:LEU:HD12	1:A:275:GLN:HE21	1.73	0.54
1:A:272:LEU:HD21	1:A:281:ILE:HG21	1.88	0.54
1:D:43:ILE:O	1:D:44:LYS:C	2.45	0.54
1:A:47:SER:HA	1:A:52:ASP:HA	1.88	0.54
9:A:606:3PE:H2A2	8:A:607:PGV:H52	1.90	0.54
1:D:356:PHE:CE2	2:F:22:MET:HG3	2.43	0.54
1:A:74:VAL:HG11	1:A:101:MET:SD	2.48	0.54
1:D:303:THR:O	1:D:307:VAL:HG12	2.07	0.54
2:F:22:MET:C	2:F:24:VAL:H	2.11	0.54
1:A:144:SER:HB2	1:A:182:TRP:HH2	1.73	0.53
1:A:168:LEU:HG	1:A:172:VAL:HG13	1.90	0.53
1:A:356:PHE:CE2	2:C:22:MET:HG3	2.43	0.53
1:D:539:SER:O	1:D:539:SER:OG	2.26	0.53
1:D:74:VAL:HG11	1:D:101:MET:SD	2.48	0.53
2:C:22:MET:C	2:C:24:VAL:H	2.11	0.53
1:A:214:ARG:NE	1:A:278:ASP:OD2	2.41	0.53
1:A:303:THR:O	1:A:307:VAL:HG12	2.07	0.53
3:E:52:HIS:ND1	3:E:56:ARG:HG3	2.24	0.53
1:D:411:GLU:O	1:D:412:VAL:HG23	2.09	0.53
1:A:240:LEU:HD13	1:A:348:PHE:HD2	1.74	0.53
1:A:393:LEU:HD22	5:A:602:HEM:HMC1	1.91	0.53

Continued on next page...

CONFIDENTIAL VALIDATION REPORT

Continued from previous page...

Atom-1	Atom-2	Interatomic distance (Å)	Clash overlap (Å)
1:A:544:LEU:O	1:A:548:SER:OG	2.18	0.53
1:D:119:VAL:O	1:D:120:LEU:HB2	2.09	0.53
1:A:270:VAL:O	1:A:273:HIS:ND1	2.40	0.52
1:D:168:LEU:HG	1:D:172:VAL:HG13	1.90	0.52
1:D:240:LEU:HD13	1:D:348:PHE:HD2	1.74	0.52
1:A:411:GLU:O	1:A:412:VAL:HG23	2.09	0.52
3:B:65:MET:HG2	3:B:66:TRP:CE2	2.44	0.52
1:A:537:VAL:O	1:A:541:ILE:HG12	2.10	0.52
1:D:272:LEU:HD12	1:D:275:GLN:HE21	1.73	0.52
1:A:539:SER:O	1:A:539:SER:OG	2.26	0.52
1:A:389:GLY:O	1:A:425:TRP:NE1	2.38	0.52
1:D:144:SER:HB2	1:D:182:TRP:HH2	1.72	0.52
1:D:537:VAL:O	1:D:541:ILE:HG12	2.10	0.52
1:A:119:VAL:O	1:A:120:LEU:HB2	2.09	0.52
1:A:524:ASN:HD22	1:A:527:THR:HG1	1.57	0.52
1:A:96:ILE:HD11	1:A:146:VAL:HG21	1.92	0.52
3:E:65:MET:HG2	3:E:66:TRP:CE2	2.44	0.52
1:A:219:PHE:CD1	1:A:540:TYR:HE2	2.28	0.51
8:A:605:PGV:O02	8:A:605:PGV:H042	2.10	0.51
3:E:99:HIS:HB2	3:E:132:HIS:CD2	2.45	0.51
3:B:99:HIS:HB2	3:B:132:HIS:CD2	2.45	0.51
1:D:219:PHE:CD1	1:D:540:TYR:HE2	2.28	0.51
3:B:52:HIS:ND1	3:B:56:ARG:HG3	2.24	0.51
1:D:270:VAL:O	1:D:273:HIS:ND1	2.40	0.51
9:D:607:3PE:C2B	9:D:607:3PE:H261	2.40	0.51
1:A:163:ASN:HD22	1:A:166:GLN:HB3	1.75	0.51
3:B:59:LEU:HG	3:B:61:ILE:HG23	1.92	0.51
1:D:289:HIS:O	1:D:293:THR:OG1	2.24	0.51
3:E:46:GLU:HA	3:E:72:LYS:NZ	2.26	0.51
1:D:309:THR:HG23	2:F:12:PRO:HB3	1.92	0.51
1:A:167:LYS:HD3	1:A:518:PRO:HA	1.92	0.51
3:B:46:GLU:HA	3:B:72:LYS:NZ	2.26	0.51
1:D:167:LYS:HD3	1:D:518:PRO:HA	1.92	0.51
8:D:601:PGV:C4	9:D:607:3PE:C2A	2.87	0.51
9:A:606:3PE:H2B1	8:D:601:PGV:C33	2.40	0.51
1:A:322:LEU:HD11	1:A:332:PHE:CD2	2.46	0.51
8:A:607:PGV:C33	9:D:607:3PE:H2B1	2.40	0.51
3:E:46:GLU:HA	3:E:72:LYS:HZ3	1.76	0.51
1:A:276:PHE:CZ	1:A:290:ALA:HB2	2.46	0.51
8:A:607:PGV:H201	8:A:607:PGV:H32	1.93	0.51
8:D:601:PGV:H32	8:D:601:PGV:H201	1.93	0.51

Continued on next page...

CONFIDENTIAL VALIDATION REPORT

Continued from previous page...

Atom-1	Atom-2	Interatomic distance (Å)	Clash overlap (Å)
8:D:606:PGV:O02	8:D:606:PGV:H042	2.10	0.51
1:A:154:PRO:C	1:A:156:ALA:H	2.14	0.50
9:A:606:3PE:C2B	9:A:606:3PE:H261	2.40	0.50
1:D:163:ASN:HD22	1:D:166:GLN:HB3	1.75	0.50
1:D:577:GLU:O	1:D:581:GLU:HB2	2.11	0.50
1:A:110:TRP:CE3	1:A:111:ALA:HB2	2.47	0.50
1:A:577:GLU:O	1:A:581:GLU:HB2	2.11	0.50
1:D:393:LEU:HD22	5:D:603:HEM:HMC1	1.91	0.50
1:D:46:GLU:O	1:D:47:SER:HB3	2.11	0.50
1:D:322:LEU:HD11	1:D:332:PHE:CD2	2.46	0.50
1:D:546:ASP:C	1:D:548:SER:H	2.14	0.50
1:D:41:GLY:O	1:D:44:LYS:HE3	2.12	0.50
1:A:119:VAL:HG13	1:A:120:LEU:N	2.23	0.50
1:A:222:HIS:CD2	1:A:273:HIS:HE1	2.30	0.50
1:A:41:GLY:O	1:A:44:LYS:HE3	2.12	0.50
1:D:182:TRP:HA	1:D:185:MET:SD	2.52	0.50
1:D:371:ASN:HD21	4:D:602:HAS:HBD2	1.77	0.50
1:D:96:ILE:HD11	1:D:146:VAL:HG21	1.92	0.50
3:E:59:LEU:HG	3:E:61:ILE:HG23	1.92	0.50
9:A:606:3PE:C2A	8:A:607:PGV:C4	2.87	0.50
1:A:309:THR:HG23	2:C:12:PRO:HB3	1.92	0.50
3:E:44:PHE:CD1	3:E:90:THR:HB	2.47	0.50
1:A:347:TYR:CD2	1:A:422:PRO:HG3	2.47	0.50
1:D:110:TRP:CE3	1:D:111:ALA:HB2	2.46	0.50
1:A:159:TRP:CH2	1:A:169:PRO:HD3	2.47	0.50
1:A:46:GLU:O	1:A:47:SER:HB3	2.11	0.50
3:B:44:PHE:CD1	3:B:90:THR:HB	2.47	0.50
1:D:124:TYR:CZ	1:D:214:ARG:NH1	2.80	0.50
1:A:546:ASP:C	1:A:548:SER:H	2.14	0.50
1:D:159:TRP:CH2	1:D:169:PRO:HD3	2.47	0.49
1:D:276:PHE:CZ	1:D:290:ALA:HB2	2.46	0.49
1:A:124:TYR:CZ	1:A:214:ARG:NH1	2.80	0.49
2:C:11:PHE:O	2:C:13:SER:N	2.46	0.49
1:D:347:TYR:CD2	1:D:422:PRO:HG3	2.47	0.49
1:D:441:VAL:O	2:F:40:ARG:NH1	2.46	0.49
1:D:154:PRO:C	1:D:156:ALA:H	2.14	0.49
1:A:542:PRO:HB2	1:A:543:PRO:HD3	1.95	0.49
1:D:230:LEU:HD21	1:D:263:PHE:CD2	2.48	0.49
1:D:371:ASN:O	1:D:375:HIS:HB2	2.13	0.49
1:D:309:THR:OG1	2:F:15:THR:HG21	2.13	0.49
1:A:182:TRP:HA	1:A:185:MET:SD	2.52	0.49

Continued on next page...

CONFIDENTIAL VALIDATION REPORT

Continued from previous page...

Atom-1	Atom-2	Interatomic distance (Å)	Clash overlap (Å)
1:A:371:ASN:O	1:A:375:HIS:HB2	2.13	0.49
1:A:167:LYS:HD2	1:A:523:ASN:HD21	1.78	0.49
1:D:240:LEU:HB3	1:D:241:PRO:HD3	1.95	0.49
1:D:446:PRO:HD3	1:D:467:TRP:CH2	2.48	0.49
1:A:230:LEU:HD21	1:A:263:PHE:CD2	2.48	0.49
1:D:170:LEU:HD23	1:D:250:LEU:HD21	1.95	0.48
1:D:542:PRO:HB2	1:D:543:PRO:HD3	1.95	0.48
1:A:170:LEU:HD23	1:A:250:LEU:HD21	1.95	0.48
1:A:371:ASN:HD21	4:A:601:HAS:HBD2	1.77	0.48
1:D:167:LYS:HD2	1:D:523:ASN:HD21	1.78	0.48
1:D:524:ASN:HD22	1:D:527:THR:HG1	1.59	0.48
3:E:19:PHE:O	3:E:23:ILE:HG23	2.13	0.48
1:A:240:LEU:HB3	1:A:241:PRO:HD3	1.95	0.48
1:A:289:HIS:O	1:A:293:THR:OG1	2.24	0.48
1:A:446:PRO:HD3	1:A:467:TRP:CH2	2.48	0.48
1:A:559:TYR:CZ	3:B:93:ASP:HB2	2.49	0.48
1:D:222:HIS:CD2	1:D:273:HIS:HE1	2.30	0.48
1:A:324:ASN:O	1:A:324:ASN:ND2	2.47	0.48
1:A:441:VAL:O	2:C:40:ARG:NH1	2.46	0.48
3:B:19:PHE:O	3:B:23:ILE:HG23	2.13	0.48
1:A:276:PHE:HZ	1:A:290:ALA:HB2	1.78	0.48
1:D:388:VAL:CG2	4:D:602:HAS:HAC	2.44	0.48
2:F:11:PHE:O	2:F:13:SER:N	2.46	0.48
1:A:309:THR:OG1	2:C:15:THR:HG21	2.13	0.48
1:A:31:HIS:CE1	1:A:56:GLY:HA3	2.49	0.48
1:D:324:ASN:ND2	1:D:324:ASN:O	2.47	0.48
1:D:276:PHE:HZ	1:D:290:ALA:HB2	1.78	0.48
1:A:388:VAL:CG2	4:A:601:HAS:HAC	2.44	0.48
1:A:130:HIS:CE1	1:A:132:THR:HG23	2.49	0.47
1:A:354:LEU:HD11	1:A:426:MET:HA	1.96	0.47
1:A:376:ASN:HD22	3:B:108:MET:HE1	1.79	0.47
1:D:31:HIS:CE1	1:D:56:GLY:HA3	2.49	0.47
1:A:327:PHE:CB	9:A:606:3PE:H2	2.43	0.47
1:A:152:TRP:CD1	1:A:176:PHE:HE1	2.32	0.47
1:D:60:HIS:NE2	5:D:603:HEM:C4D	2.77	0.47
1:D:354:LEU:HD11	1:D:426:MET:HA	1.96	0.47
1:D:559:TYR:CZ	3:E:93:ASP:HB2	2.48	0.47
1:D:130:HIS:CE1	1:D:132:THR:HG23	2.49	0.47
1:A:384:PHE:HE1	5:A:602:HEM:C3D	2.33	0.46
1:A:464:ARG:HG2	1:A:467:TRP:CE2	2.50	0.46
3:E:72:LYS:HD2	3:E:72:LYS:HA	1.63	0.46

Continued on next page...

CONFIDENTIAL VALIDATION REPORT

Continued from previous page...

Atom-1	Atom-2	Interatomic distance (Å)	Clash overlap (Å)
1:A:282:THR:HG22	1:A:283:ASN:O	2.15	0.46
1:A:60:HIS:NE2	5:A:602:HEM:C4D	2.77	0.46
1:D:152:TRP:CD1	1:D:176:PHE:HE1	2.32	0.46
1:D:384:PHE:HE1	5:D:603:HEM:C3D	2.33	0.46
1:A:432:PHE:HB2	1:A:477:GLY:HA3	1.98	0.46
1:D:430:PHE:CE2	8:D:601:PGV:H91	2.50	0.46
1:D:327:PHE:CB	9:D:607:3PE:H2	2.44	0.46
1:A:534:ILE:O	1:A:538:LEU:HG	2.16	0.46
2:C:37:LEU:HD12	3:B:104:ASN:HD22	1.80	0.46
1:D:43:ILE:HG13	1:D:43:ILE:O	2.16	0.46
1:D:464:ARG:HG2	1:D:467:TRP:CE2	2.50	0.46
2:F:37:LEU:HD12	3:E:104:ASN:HD22	1.81	0.46
8:A:605:PGV:H172	8:A:605:PGV:H141	1.79	0.46
1:D:534:ILE:HD13	1:D:534:ILE:HA	1.78	0.46
1:A:430:PHE:CE2	8:A:607:PGV:H91	2.50	0.46
1:D:281:ILE:HB	1:D:286:LYS:HE3	1.98	0.46
8:D:601:PGV:H41	9:D:607:3PE:H2A1	1.96	0.46
1:A:230:LEU:HD23	1:A:230:LEU:HA	1.60	0.46
1:D:230:LEU:HA	1:D:230:LEU:HD23	1.60	0.46
1:D:376:ASN:HD22	3:E:108:MET:HE1	1.80	0.46
2:F:39:GLU:O	2:F:39:GLU:HG2	2.16	0.46
9:A:606:3PE:H2B2	9:A:606:3PE:H261	1.98	0.46
1:D:125:PRO:HG3	1:D:213:ALA:CB	2.46	0.46
1:D:318:GLU:HG3	1:D:319:HIS:CD2	2.51	0.46
1:A:43:ILE:O	1:A:43:ILE:HG13	2.16	0.45
1:D:534:ILE:O	1:D:538:LEU:HG	2.16	0.45
1:D:544:LEU:HD12	1:D:579:LYS:NZ	2.31	0.45
1:A:276:PHE:CD1	1:A:286:LYS:HB3	2.51	0.45
9:A:606:3PE:C2A	8:A:607:PGV:H52	2.46	0.45
1:D:432:PHE:HB2	1:D:477:GLY:HA3	1.98	0.45
1:D:576:LYS:HA	1:D:576:LYS:HD2	1.59	0.45
8:D:601:PGV:H52	9:D:607:3PE:C2A	2.45	0.45
3:E:35:PRO:HG2	3:E:107:VAL:HG22	1.98	0.45
3:E:45:GLN:O	3:E:46:GLU:HG2	2.16	0.45
1:A:196:GLN:OE1	1:A:213:ALA:HB2	2.17	0.45
1:A:576:LYS:HA	1:A:576:LYS:HD2	1.59	0.45
1:D:282:THR:HG22	1:D:283:ASN:O	2.15	0.45
1:A:420:LEU:HD12	9:D:607:3PE:H331	1.97	0.45
3:B:45:GLN:O	3:B:46:GLU:HG2	2.16	0.45
1:D:293:THR:HG22	4:D:602:HAS:HMB2	1.98	0.45
2:F:26:TYR:C	2:F:28:VAL:H	2.20	0.45

Continued on next page...

CONFIDENTIAL VALIDATION REPORT

Continued from previous page...

Atom-1	Atom-2	Interatomic distance (Å)	Clash overlap (Å)
1:A:125:PRO:HG3	1:A:213:ALA:CB	2.46	0.45
1:A:465:PRO:HG2	1:D:465:PRO:HG2	1.98	0.45
1:A:505:THR:OG1	1:A:506:LEU:N	2.50	0.45
1:D:457:ASN:C	1:D:459:ASP:H	2.20	0.45
1:A:457:ASN:C	1:A:459:ASP:H	2.20	0.45
1:D:276:PHE:CD1	1:D:286:LYS:HB3	2.51	0.45
3:E:42:GLU:CB	3:E:114:VAL:HG21	2.42	0.45
1:A:469:GLY:HA3	1:D:469:GLY:HA3	1.97	0.45
3:E:48:LYS:O	3:E:49:LEU:HD23	2.17	0.45
1:A:360:ILE:HD13	2:C:26:TYR:CG	2.52	0.45
9:A:606:3PE:H331	1:D:420:LEU:HD12	1.98	0.45
1:A:228:TRP:HB3	1:A:392:VAL:HG11	1.99	0.45
1:D:196:GLN:OE1	1:D:213:ALA:HB2	2.17	0.45
1:D:388:VAL:HG23	4:D:602:HAS:HAC	1.99	0.45
1:A:336:MET:CE	9:D:607:3PE:C27	2.88	0.44
9:A:606:3PE:H32	9:A:606:3PE:H322	1.87	0.44
1:D:24:LEU:HA	1:D:24:LEU:HD12	1.69	0.44
1:D:327:PHE:CZ	2:F:17:ALA:HB1	2.52	0.44
1:D:360:ILE:HD13	2:F:26:TYR:CG	2.52	0.44
1:A:186:ILE:HA	1:A:186:ILE:HD13	1.64	0.44
1:A:281:ILE:HB	1:A:286:LYS:HE3	1.98	0.44
1:A:388:VAL:HG23	4:A:601:HAS:HAC	1.99	0.44
3:B:35:PRO:HG2	3:B:107:VAL:HG22	1.98	0.44
2:C:39:GLU:HG2	2:C:39:GLU:O	2.16	0.44
1:D:236:LEU:HB3	1:D:307:VAL:HG21	2.00	0.44
2:F:23:MET:SD	3:E:11:LEU:HB3	2.58	0.44
1:A:327:PHE:CZ	2:C:17:ALA:HB1	2.52	0.44
1:A:33:VAL:CG2	1:A:482:ALA:HB2	2.48	0.44
1:A:540:TYR:HA	1:A:543:PRO:HD2	1.99	0.44
1:A:544:LEU:HD12	1:A:579:LYS:NZ	2.31	0.44
4:A:601:HAS:H251	4:A:601:HAS:H282	1.76	0.44
9:A:606:3PE:C2A	8:A:607:PGV:H41	2.48	0.44
8:D:601:PGV:H282	8:D:601:PGV:H252	1.56	0.44
8:A:607:PGV:C31	9:D:607:3PE:H261	2.48	0.44
1:A:293:THR:HG22	4:A:601:HAS:HMB2	1.98	0.44
8:D:601:PGV:H41	9:D:607:3PE:C2A	2.48	0.44
1:A:497:LEU:HA	1:A:497:LEU:HD23	1.69	0.44
9:A:606:3PE:H261	8:D:601:PGV:C31	2.48	0.44
3:B:78:LYS:HA	3:B:147:ILE:O	2.18	0.44
1:A:29:ILE:HG23	5:A:602:HEM:CBB	2.48	0.44
1:A:318:GLU:HG3	1:A:319:HIS:CD2	2.51	0.44

Continued on next page...

CONFIDENTIAL VALIDATION REPORT

Continued from previous page...

Atom-1	Atom-2	Interatomic distance (Å)	Clash overlap (Å)
1:A:367:SER:HB2	1:A:370:VAL:HB	2.00	0.44
1:D:211:LEU:HD13	3:E:110:ILE:HD13	2.00	0.44
1:D:228:TRP:HB3	1:D:392:VAL:HG11	1.99	0.44
1:D:505:THR:OG1	1:D:506:LEU:N	2.50	0.44
3:B:42:GLU:CB	3:B:114:VAL:HG21	2.42	0.43
1:D:119:VAL:HG13	1:D:120:LEU:N	2.23	0.43
1:D:100:MET:HG2	1:D:139:LEU:HD21	2.00	0.43
1:D:261:ILE:HA	1:D:261:ILE:HD13	1.85	0.43
1:A:215:THR:HG22	1:A:216:LEU:H	1.83	0.43
1:A:236:LEU:HB3	1:A:307:VAL:HG21	2.00	0.43
2:C:23:MET:SD	3:B:11:LEU:HB3	2.58	0.43
3:B:57:TYR:HB2	3:B:85:VAL:HG12	2.00	0.43
1:D:33:VAL:CG2	1:D:482:ALA:HB2	2.48	0.43
3:E:61:ILE:HD13	3:E:61:ILE:HG21	1.72	0.43
1:A:100:MET:HG2	1:A:139:LEU:HD21	2.00	0.43
1:A:211:LEU:HD13	3:B:110:ILE:HD13	2.00	0.43
1:D:215:THR:HG22	1:D:216:LEU:H	1.83	0.43
1:A:354:LEU:HA	1:A:354:LEU:HD23	1.77	0.43
1:A:544:LEU:HA	1:A:544:LEU:HD23	1.74	0.43
2:C:26:TYR:C	2:C:28:VAL:H	2.20	0.43
1:D:29:ILE:HG23	5:D:603:HEM:CBB	2.48	0.43
3:E:78:LYS:HA	3:E:147:ILE:O	2.18	0.43
1:A:24:LEU:HA	1:A:24:LEU:HD12	1.69	0.43
1:D:540:TYR:HA	1:D:543:PRO:HD2	1.99	0.43
1:D:438:VAL:O	1:D:442:VAL:HG22	2.19	0.43
1:A:329:TRP:C	1:A:331:THR:H	2.22	0.43
1:D:497:LEU:HA	1:D:497:LEU:HD23	1.69	0.43
1:A:285:TRP:CE2	1:A:542:PRO:HB3	2.54	0.43
1:D:586:GLU:O	1:D:590:THR:OG1	2.27	0.43
8:F:101:PGV:H322	8:F:101:PGV:H291	1.81	0.43
1:A:369:ASN:HD21	3:B:27:ALA:N	2.16	0.43
2:C:37:LEU:CD1	3:B:104:ASN:HD22	2.32	0.43
3:B:72:LYS:HA	3:B:72:LYS:HD2	1.63	0.43
1:D:114:THR:OG1	1:D:116:ARG:HD3	2.19	0.43
1:D:329:TRP:C	1:D:331:THR:H	2.22	0.43
1:A:15:LEU:HD23	1:A:15:LEU:HA	1.69	0.43
3:B:48:LYS:O	3:B:49:LEU:HD23	2.17	0.43
3:B:78:LYS:HE2	3:B:149:GLU:HB2	2.01	0.43
1:D:15:LEU:HA	1:D:15:LEU:HD23	1.69	0.43
1:D:369:ASN:HD21	3:E:27:ALA:N	2.16	0.43
1:D:393:LEU:HD13	5:D:603:HEM:HBC2	2.01	0.43

Continued on next page...

CONFIDENTIAL VALIDATION REPORT

Continued from previous page...

Atom-1	Atom-2	Interatomic distance (Å)	Clash overlap (Å)
1:D:465:PRO:O	1:D:468:THR:HG23	2.19	0.43
1:D:285:TRP:CE2	1:D:542:PRO:HB3	2.54	0.43
2:F:37:LEU:CD1	3:E:104:ASN:HD22	2.32	0.43
3:E:78:LYS:HE2	3:E:149:GLU:HB2	2.01	0.43
1:A:229:LEU:HD22	4:A:601:HAS:HMC2	2.00	0.42
9:A:606:3PE:C27	1:D:336:MET:CE	2.89	0.42
1:D:357:ILE:HG21	7:D:605:DLX:C44	2.49	0.42
1:D:360:ILE:HD13	2:F:26:TYR:CB	2.49	0.42
1:A:114:THR:OG1	1:A:116:ARG:HD3	2.19	0.42
9:A:606:3PE:H2B2	9:A:606:3PE:H281	1.87	0.42
1:A:360:ILE:HD13	2:C:26:TYR:CB	2.49	0.42
1:D:229:LEU:HD22	4:D:602:HAS:HMC2	2.00	0.42
1:D:522:LEU:O	1:D:523:ASN:HB2	2.19	0.42
1:A:357:ILE:HG21	7:A:604:DLX:C44	2.49	0.42
1:A:458:PRO:HA	1:A:463:TYR:CE1	2.54	0.42
1:A:465:PRO:O	1:A:468:THR:HG23	2.19	0.42
1:D:270:VAL:HG22	1:D:273:HIS:CE1	2.55	0.42
1:D:458:PRO:HA	1:D:463:TYR:CE1	2.54	0.42
1:D:524:ASN:O	1:D:528:TRP:HD1	2.02	0.42
4:D:602:HAS:H271	4:D:602:HAS:H212	1.70	0.42
1:A:393:LEU:HD13	5:A:602:HEM:HBC2	2.01	0.42
2:C:34:TYR:OH	3:B:26:ALA:HB2	2.20	0.42
1:D:214:ARG:HG2	1:D:275:GLN:OE1	2.19	0.42
1:D:367:SER:HB2	1:D:370:VAL:HB	2.00	0.42
9:D:607:3PE:H2B2	9:D:607:3PE:H281	1.87	0.42
1:D:82:ALA:HB2	1:D:89:LEU:HG	2.01	0.42
1:A:214:ARG:HG2	1:A:275:GLN:OE1	2.19	0.42
1:A:438:VAL:O	1:A:442:VAL:HG22	2.18	0.42
1:A:427:GLN:CB	8:A:607:PGV:H251	2.49	0.42
3:B:120:LYS:HE2	3:B:120:LYS:HB3	1.74	0.42
1:D:311:LEU:HD23	1:D:311:LEU:HA	1.74	0.42
1:D:506:LEU:HA	1:D:506:LEU:HD12	1.88	0.42
1:A:167:LYS:HE3	1:A:167:LYS:HB2	1.70	0.42
1:A:208:ILE:HG22	1:A:556:SER:O	2.20	0.42
1:A:522:LEU:O	1:A:523:ASN:HB2	2.19	0.42
9:A:606:3PE:H2A1	8:A:607:PGV:H41	1.96	0.42
3:B:101:HIS:CE1	3:B:130:VAL:HG11	2.55	0.42
1:D:200:LEU:HD22	1:D:208:ILE:HB	2.02	0.42
1:D:242:LYS:HB3	1:D:509:PRO:HB2	2.01	0.42
3:E:120:LYS:HB3	3:E:120:LYS:HE2	1.74	0.42
2:F:22:MET:C	2:F:24:VAL:N	2.73	0.42

Continued on next page...

CONFIDENTIAL VALIDATION REPORT

Continued from previous page...

Atom-1	Atom-2	Interatomic distance (Å)	Clash overlap (Å)
1:A:181:LEU:HD12	1:A:181:LEU:HA	1.77	0.42
1:D:209:ASN:OD1	1:D:552:VAL:HG11	2.20	0.42
3:E:101:HIS:CE1	3:E:130:VAL:HG11	2.55	0.42
3:E:78:LYS:HE3	3:E:149:GLU:OE1	2.20	0.42
3:E:57:TYR:HB2	3:E:85:VAL:HG12	2.00	0.42
1:A:517:ALA:HA	1:A:518:PRO:HD3	1.90	0.42
3:B:105:TYR:CE1	3:B:117:MET:HB3	2.55	0.42
1:D:23:LEU:HD23	1:D:23:LEU:HA	1.74	0.42
4:D:602:HAS:H251	4:D:602:HAS:H282	1.76	0.42
1:A:144:SER:O	1:A:147:PRO:HD2	2.20	0.41
1:A:200:LEU:HD22	1:A:208:ILE:HB	2.02	0.41
1:A:270:VAL:HG22	1:A:273:HIS:CE1	2.55	0.41
4:A:601:HAS:H262	4:A:601:HAS:H132	1.80	0.41
1:D:541:ILE:HB	1:D:542:PRO:HD3	2.02	0.41
1:D:544:LEU:O	1:D:548:SER:OG	2.18	0.41
1:A:261:ILE:HD13	1:A:261:ILE:HA	1.85	0.41
1:A:298:LEU:N	1:A:299:PRO:HD2	2.36	0.41
1:A:524:ASN:O	1:A:528:TRP:HD1	2.02	0.41
8:C:101:PGV:H291	8:C:101:PGV:H322	1.81	0.41
1:A:215:THR:C	1:A:217:PHE:N	2.73	0.41
1:D:110:TRP:CZ3	1:D:111:ALA:HB2	2.56	0.41
1:D:278:ASP:O	1:D:286:LYS:NZ	2.42	0.41
4:D:602:HAS:H262	4:D:602:HAS:H132	1.80	0.41
1:A:361:THR:HG22	1:A:383:HIS:HB2	2.03	0.41
4:A:601:HAS:H271	4:A:601:HAS:H212	1.70	0.41
1:D:361:THR:HG22	1:D:383:HIS:HB2	2.03	0.41
1:D:473:LEU:HD23	1:D:473:LEU:HA	1.81	0.41
1:D:60:HIS:CD2	5:D:603:HEM:C4D	3.09	0.41
2:F:34:TYR:OH	3:E:26:ALA:HB2	2.20	0.41
1:A:242:LYS:HB3	1:A:509:PRO:HB2	2.01	0.41
1:A:381:PRO:HD3	1:A:447:ARG:HD2	2.02	0.41
8:A:607:PGV:H31	8:A:607:PGV:H62	1.87	0.41
1:A:82:ALA:HB2	1:A:89:LEU:HG	2.01	0.41
3:B:78:LYS:HE3	3:B:149:GLU:OE1	2.20	0.41
1:D:215:THR:C	1:D:217:PHE:N	2.73	0.41
1:D:381:PRO:HD3	1:D:447:ARG:HD2	2.02	0.41
1:D:208:ILE:HG22	1:D:556:SER:O	2.20	0.41
3:E:105:TYR:CE1	3:E:117:MET:HB3	2.55	0.41
1:A:244:VAL:HG12	1:A:245:SER:N	2.34	0.41
1:A:60:HIS:CD2	5:A:602:HEM:C4D	3.09	0.41
1:D:153:ILE:HA	1:D:153:ILE:HD13	1.85	0.41

Continued on next page...

CONFIDENTIAL VALIDATION REPORT

Continued from previous page...

Atom-1	Atom-2	Interatomic distance (Å)	Clash overlap (Å)
1:D:427:GLN:CB	8:D:601:PGV:H251	2.51	0.41
1:A:357:ILE:HG13	7:A:604:DLX:C28	2.51	0.41
1:A:209:ASN:OD1	1:A:552:VAL:HG11	2.20	0.41
3:B:9:LEU:HD12	3:B:9:LEU:HA	1.76	0.41
1:D:298:LEU:N	1:D:299:PRO:HD2	2.36	0.41
1:D:420:LEU:HD23	1:D:420:LEU:HA	1.84	0.41
1:A:23:LEU:HD23	1:A:23:LEU:HA	1.74	0.41
1:D:517:ALA:HA	1:D:518:PRO:HD3	1.90	0.41
1:A:13:ILE:HA	1:A:13:ILE:HD13	1.83	0.41
1:D:219:PHE:CD1	1:D:540:TYR:CE2	3.09	0.41
1:A:282:THR:HB	1:A:285:TRP:CD1	2.56	0.41
2:C:22:MET:C	2:C:24:VAL:N	2.73	0.41
1:D:144:SER:O	1:D:147:PRO:HD2	2.20	0.41
1:D:167:LYS:HB2	1:D:167:LYS:HE3	1.70	0.41
1:A:110:TRP:CZ3	1:A:111:ALA:HB2	2.56	0.40
1:A:185:MET:HG2	1:A:223:PRO:HB2	2.02	0.40
1:A:457:ASN:O	1:A:459:ASP:N	2.54	0.40
7:A:604:DLX:C29	8:A:607:PGV:H131	2.52	0.40
2:C:15:THR:HG23	2:C:15:THR:H	1.60	0.40
1:D:13:ILE:HA	1:D:13:ILE:HD13	1.83	0.40
1:D:282:THR:HB	1:D:285:TRP:CD1	2.56	0.40
1:D:457:ASN:O	1:D:459:ASP:N	2.54	0.40
1:D:357:ILE:HG13	7:D:605:DLX:C28	2.51	0.40
1:A:473:LEU:HA	1:A:473:LEU:HD23	1.81	0.40
3:B:84:VAL:HB	3:B:120:LYS:HE2	2.03	0.40
1:A:541:ILE:HB	1:A:542:PRO:HD3	2.02	0.40
3:B:41:VAL:HG13	3:B:42:GLU:N	2.35	0.40
1:D:244:VAL:HG12	1:D:245:SER:N	2.34	0.40
8:D:601:PGV:H131	7:D:605:DLX:C29	2.50	0.40
3:E:106:ASN:OD1	3:E:132:HIS:NE2	2.52	0.40
1:A:120:LEU:HA	1:A:120:LEU:HD12	1.71	0.40
1:A:219:PHE:CD1	1:A:540:TYR:CE2	3.09	0.40
1:A:493:ILE:HA	1:A:493:ILE:HD13	1.85	0.40
1:D:225:VAL:HA	1:D:228:TRP:CE3	2.56	0.40
1:D:65:VAL:HB	1:D:228:TRP:CH2	2.56	0.40
1:A:157:ILE:HA	1:A:157:ILE:HD13	1.89	0.40
1:A:225:VAL:HA	1:A:228:TRP:CE3	2.56	0.40
1:A:262:LEU:HD23	1:A:299:PRO:HD3	2.03	0.40
1:A:417:LEU:HD12	1:A:487:PHE:CD1	2.57	0.40
3:B:61:ILE:HG21	3:B:61:ILE:HD13	1.72	0.40
1:D:85:LEU:HD13	1:D:155:SER:O	2.21	0.40

Continued on next page...

CONFIDENTIAL VALIDATION REPORT

Continued from previous page...

Atom-1	Atom-2	Interatomic distance (Å)	Clash overlap (Å)
1:D:200:LEU:HD21	1:D:558:ALA:HB2	2.02	0.40

There are no symmetry-related clashes.

5.3 Torsion angles (i)

5.3.1 Protein backbone (i)

In the following table, the Percentiles column shows the percent Ramachandran outliers of the chain as a percentile score with respect to all PDB entries followed by that with respect to all EM entries.

The Analysed column shows the number of residues for which the backbone conformation was analysed, and the total number of residues.

Mol	Chain	Analysed	Favoured	Allowed	Outliers	Percentiles	
1	A	577/587 (98%)	482 (84%)	93 (16%)	2 (0%)	41	72
1	D	577/587 (98%)	481 (83%)	94 (16%)	2 (0%)	41	72
2	C	30/32 (94%)	25 (83%)	4 (13%)	1 (3%)	4	22
2	F	30/32 (94%)	25 (83%)	4 (13%)	1 (3%)	4	22
3	B	145/147 (99%)	118 (81%)	27 (19%)	0	100	100
3	E	145/147 (99%)	118 (81%)	27 (19%)	0	100	100
All	All	1504/1532 (98%)	1249 (83%)	249 (17%)	6 (0%)	38	67

All (6) Ramachandran outliers are listed below:

Mol	Chain	Res	Type
1	A	44	LYS
1	D	44	LYS
1	A	46	GLU
1	D	46	GLU
2	C	23	MET
2	F	23	MET

5.3.2 Protein sidechains (i)

In the following table, the Percentiles column shows the percent sidechain outliers of the chain as a percentile score with respect to all PDB entries followed by that with respect to all EM

CONFIDENTIAL VALIDATION REPORT

entries.

The Analysed column shows the number of residues for which the sidechain conformation was analysed, and the total number of residues.

Mol	Chain	Analysed	Rotameric	Outliers	Percentiles	
1	A	486/492 (99%)	480 (99%)	6 (1%)	71	85
1	D	486/492 (99%)	480 (99%)	6 (1%)	71	85
2	C	28/28 (100%)	28 (100%)	0	100	100
2	F	28/28 (100%)	28 (100%)	0	100	100
3	B	125/125 (100%)	125 (100%)	0	100	100
3	E	125/125 (100%)	125 (100%)	0	100	100
All	All	1278/1290 (99%)	1266 (99%)	12 (1%)	79	90

All (12) residues with a non-rotameric sidechain are listed below:

Mol	Chain	Res	Type
1	A	39	ARG
1	A	40	SER
1	A	42	ILE
1	A	214	ARG
1	A	219	PHE
1	A	575	LYS
1	D	39	ARG
1	D	40	SER
1	D	42	ILE
1	D	214	ARG
1	D	219	PHE
1	D	575	LYS

Some sidechains can be flipped to improve hydrogen bonding and reduce clashes. All (19) such sidechains are listed below:

Mol	Chain	Res	Type
1	A	94	GLN
1	A	275	GLN
1	A	341	ASN
1	A	369	ASN
1	A	371	ASN
1	A	375	HIS
1	A	376	ASN
1	A	472	GLN
1	A	524	ASN

Continued on next page...

CONFIDENTIAL VALIDATION REPORT

Continued from previous page...

Mol	Chain	Res	Type
1	A	560	ASN
1	D	94	GLN
1	D	341	ASN
1	D	369	ASN
1	D	371	ASN
1	D	375	HIS
1	D	376	ASN
1	D	472	GLN
1	D	524	ASN
1	D	560	ASN

5.3.3 RNA (i)

There are no RNA molecules in this entry.

5.4 Non-standard residues in protein, DNA, RNA chains (i)

There are no non-standard protein/DNA/RNA residues in this entry.

5.5 Carbohydrates (i)

There are no monosaccharides in this entry.

5.6 Ligand geometry (i)

Of 18 ligands modelled in this entry, 2 are monoatomic - leaving 16 for Mogul analysis.

In the following table, the Counts columns list the number of bonds (or angles) for which Mogul statistics could be retrieved, the number of bonds (or angles) that are observed in the model and the number of bonds (or angles) that are defined in the Chemical Component Dictionary. The Link column lists molecule types, if any, to which the group is linked. The Z score for a bond length (or angle) is the number of standard deviations the observed value is removed from the expected value. A bond length (or angle) with $|Z| > 2$ is considered an outlier worth inspection. RMSZ is the root-mean-square of all Z scores of the bond lengths (or angles).

Mol	Type	Chain	Res	Link	Bond lengths			Bond angles		
					Counts	RMSZ	$\#\|Z\| > 2$	Counts	RMSZ	$\#\|Z\| > 2$
8	PGV	D	606	-	50,50,50	0.87	2 (4%)	53,56,56	1.10	5 (9%)
5	HEM	A	602	1	27,50,50	2.09	7 (25%)	17,82,82	2.11	5 (29%)
8	PGV	C	101	-	50,50,50	0.91	2 (4%)	53,56,56	1.09	4 (7%)

CONFIDENTIAL VALIDATION REPORT

Mol	Type	Chain	Res	Link	Bond lengths			Bond angles		
					Counts	RMSZ	#|Z| > 2	Counts	RMSZ	#|Z| > 2
8	PGV	A	607	8	50,50,50	0.94	2 (4%)	53,56,56	1.15	3 (5%)
4	HAS	D	602	1,2	56,72,72	4.13	18 (32%)	50,109,109	5.40	32 (64%)
7	DLX	A	604	1,2	48,48,48	3.24	12 (25%)	59,61,61	3.24	22 (37%)
8	PGV	F	101	-	50,50,50	0.91	2 (4%)	53,56,56	1.09	4 (7%)
4	HAS	A	601	1,2	56,72,72	4.13	18 (32%)	50,109,109	5.40	32 (64%)
8	PGV	A	605	-	50,50,50	0.87	2 (4%)	53,56,56	1.10	5 (9%)
10	CUA	E	600	3	0,1,1	0.00	-	-		
5	HEM	D	603	1	27,50,50	2.08	7 (25%)	17,82,82	2.11	5 (29%)
8	PGV	D	601	8	50,50,50	0.94	2 (4%)	53,56,56	1.15	3 (5%)
9	3PE	D	607	-	50,50,50	0.26	0	53,55,55	0.31	0
10	CUA	B	600	3	0,1,1	0.00	-	-		
9	3PE	A	606	-	50,50,50	0.26	0	53,55,55	0.30	0
7	DLX	D	605	1,2	48,48,48	3.23	12 (25%)	59,61,61	3.24	22 (37%)

In the following table, the Chirals column lists the number of chiral outliers, the number of chiral centers analysed, the number of these observed in the model and the number defined in the Chemical Component Dictionary. Similar counts are reported in the Torsion and Rings columns. '-' means no outliers of that kind were identified.

Mol	Type	Chain	Res	Link	Chirals	Torsions	Rings
8	PGV	D	606	-	-	16/55/55/55	-
7	DLX	A	604	1,2	-	28/41/57/57	0/2/2/2
8	PGV	C	101	-	-	21/55/55/55	-
8	PGV	A	607	8	-	28/55/55/55	-
4	HAS	D	602	1,2	-	20/35/122/122	-
8	PGV	F	101	-	-	21/55/55/55	-
4	HAS	A	601	1,2	-	20/35/122/122	-
8	PGV	A	605	-	-	16/55/55/55	-
5	HEM	D	603	1	-	0/6/54/54	-
8	PGV	D	601	8	-	28/55/55/55	-
9	3PE	D	607	-	-	28/54/54/54	-
5	HEM	A	602	1	-	0/6/54/54	-
9	3PE	A	606	-	-	28/54/54/54	-
7	DLX	D	605	1,2	-	28/41/57/57	0/2/2/2

All (86) bond length outliers are listed below:

CONFIDENTIAL VALIDATION REPORT

Mol	Chain	Res	Type	Atoms	Z	Observed(Å)	Ideal(Å)
4	D	602	HAS	C1D-ND	-11.77	1.33	1.49
4	A	601	HAS	C1D-ND	-11.77	1.33	1.49
4	D	602	HAS	C4D-ND	-11.46	1.34	1.49
4	A	601	HAS	C4D-ND	-11.43	1.34	1.49
4	A	601	HAS	C1B-NB	-11.24	1.34	1.49
4	D	602	HAS	C1B-NB	-11.22	1.34	1.49
4	D	602	HAS	C4B-NB	-10.98	1.34	1.49
4	A	601	HAS	C4B-NB	-10.98	1.34	1.49
7	A	604	DLX	C26-C27	8.47	1.53	1.33
7	A	604	DLX	C30-C31	8.45	1.53	1.33
7	D	605	DLX	C30-C31	8.45	1.53	1.33
7	D	605	DLX	C26-C27	8.45	1.53	1.33
4	D	602	HAS	C22-C23	8.01	1.52	1.33
4	A	601	HAS	C22-C23	8.01	1.52	1.33
7	D	605	DLX	C22-C23	7.99	1.52	1.33
7	A	604	DLX	C22-C23	7.98	1.52	1.33
4	D	602	HAS	C18-C19	7.97	1.52	1.33
4	A	601	HAS	C18-C19	7.93	1.52	1.33
7	A	604	DLX	C14-C15	7.86	1.51	1.33
7	D	605	DLX	C14-C15	7.83	1.51	1.33
4	A	601	HAS	C14-C15	7.81	1.51	1.33
4	D	602	HAS	C14-C15	7.79	1.51	1.33
7	A	604	DLX	C18-C19	7.74	1.51	1.33
7	D	605	DLX	C18-C19	7.73	1.51	1.33
4	D	602	HAS	C29-C30	7.34	1.53	1.32
4	A	601	HAS	C29-C30	7.32	1.53	1.32
4	D	602	HAS	C4A-C3A	6.47	1.47	1.38
4	A	601	HAS	C4A-C3A	6.47	1.47	1.38
4	D	602	HAS	C1C-C2C	6.35	1.46	1.38
5	A	602	HEM	C3B-C2B	-6.35	1.31	1.40
4	A	601	HAS	C1C-C2C	6.33	1.46	1.38
5	D	603	HEM	C3B-C2B	-6.32	1.31	1.40
4	D	602	HAS	C1A-C2A	6.04	1.46	1.38
7	D	605	DLX	O12-C04	6.03	1.36	1.23
7	A	604	DLX	O12-C04	6.03	1.36	1.23
4	A	601	HAS	C1A-C2A	6.02	1.46	1.38
5	A	602	HEM	C3C-C2C	-5.92	1.32	1.40
5	D	603	HEM	C3C-C2C	-5.92	1.32	1.40
7	A	604	DLX	O11-C01	5.64	1.36	1.24
7	D	605	DLX	O11-C01	5.64	1.36	1.24
7	A	604	DLX	C05-C04	-5.28	1.37	1.48
7	D	605	DLX	C05-C04	-5.26	1.37	1.48
7	D	605	DLX	C03-C04	-5.19	1.38	1.48

Continued on next page...

CONFIDENTIAL VALIDATION REPORT

Continued from previous page...

Mol	Chain	Res	Type	Atoms	Z	Observed(Å)	Ideal(Å)
7	A	604	DLX	C02-C01	-5.15	1.39	1.48
7	D	605	DLX	C02-C01	-5.15	1.39	1.48
7	A	604	DLX	C03-C04	-5.14	1.38	1.48
8	A	607	PGV	O01-C1	4.37	1.46	1.34
8	D	601	PGV	O01-C1	4.37	1.46	1.34
8	C	101	PGV	O01-C1	4.17	1.46	1.34
8	F	101	PGV	O01-C1	4.17	1.46	1.34
8	C	101	PGV	O03-C19	4.16	1.45	1.33
8	F	101	PGV	O03-C19	4.16	1.45	1.33
8	A	607	PGV	O03-C19	4.04	1.45	1.33
8	D	601	PGV	O03-C19	4.04	1.45	1.33
8	D	606	PGV	O03-C19	3.81	1.44	1.33
8	A	605	PGV	O03-C19	3.81	1.44	1.33
8	D	606	PGV	O01-C1	3.79	1.45	1.34
8	A	605	PGV	O01-C1	3.79	1.45	1.34
4	A	601	HAS	C3C-C2C	-3.39	1.35	1.40
4	D	602	HAS	C3C-C2C	-3.36	1.35	1.40
4	A	601	HAS	C1D-C2D	-3.06	1.46	1.51
4	D	602	HAS	C1D-C2D	-3.04	1.46	1.51
4	D	602	HAS	C11-C3B	-3.04	1.47	1.51
4	A	601	HAS	C11-C3B	-3.04	1.47	1.51
5	D	603	HEM	C3B-CAB	2.90	1.53	1.47
5	A	602	HEM	C3B-CAB	2.90	1.53	1.47
4	D	602	HAS	C2B-C3B	2.90	1.36	1.34
4	A	601	HAS	C2B-C3B	2.83	1.36	1.34
4	D	602	HAS	CHA-C4D	-2.81	1.48	1.53
4	A	601	HAS	CHA-C4D	-2.81	1.48	1.53
7	A	604	DLX	C06-C01	-2.58	1.39	1.44
7	D	605	DLX	C06-C01	-2.58	1.39	1.44
5	A	602	HEM	C2A-C3A	-2.46	1.30	1.37
5	D	603	HEM	C2A-C3A	-2.44	1.30	1.37
5	A	602	HEM	C3C-CAC	2.34	1.52	1.47
5	D	603	HEM	C3C-CAC	2.31	1.52	1.47
5	D	603	HEM	C3D-C2D	-2.17	1.31	1.37
5	A	602	HEM	C3D-C2D	-2.14	1.31	1.37
4	A	601	HAS	CHD-C4A	-2.10	1.48	1.51
4	D	602	HAS	CHD-C4A	-2.09	1.49	1.51
7	D	605	DLX	C28-C27	2.08	1.55	1.51
7	A	604	DLX	C28-C27	2.08	1.55	1.51
4	D	602	HAS	CHC-C4B	-2.06	1.49	1.53
5	D	603	HEM	C1D-CHD	-2.06	1.35	1.41
4	A	601	HAS	CHC-C4B	-2.03	1.49	1.53

Continued on next page...

CONFIDENTIAL VALIDATION REPORT

Continued from previous page...

Mol	Chain	Res	Type	Atoms	Z	Observed(Å)	Ideal(Å)
5	A	602	HEM	C1D-CHD	-2.03	1.35	1.41

All (142) bond angle outliers are listed below:

Mol	Chain	Res	Type	Atoms	Z	Observed(^{o})	Ideal(^{o})
4	D	602	HAS	CHB-C1D-ND	16.38	131.31	110.94
4	A	601	HAS	CHB-C1D-ND	16.35	131.26	110.94
4	A	601	HAS	CHB-C1B-NB	16.21	131.10	110.94
4	D	602	HAS	CHB-C1B-NB	16.21	131.09	110.94
4	A	601	HAS	CHA-C4D-ND	10.69	130.91	110.75
4	D	602	HAS	CHA-C4D-ND	10.67	130.87	110.75
4	A	601	HAS	CAA-C2A-C1A	-10.08	120.22	127.30
4	D	602	HAS	CAA-C2A-C1A	-10.03	120.25	127.30
7	D	605	DLX	C13-C14-C15	-10.03	110.10	126.79
7	A	604	DLX	C13-C14-C15	-10.02	110.10	126.79
4	A	601	HAS	CHC-C4B-NB	9.69	129.03	110.75
4	D	602	HAS	CHC-C4B-NB	9.67	128.99	110.75
7	A	604	DLX	C16-C15-C14	-7.09	106.78	121.12
4	D	602	HAS	C17-C18-C19	-7.08	110.61	127.66
7	D	605	DLX	C16-C15-C14	-7.08	106.80	121.12
4	A	601	HAS	C17-C18-C19	-7.07	110.63	127.66
4	A	601	HAS	C21-C22-C23	-6.92	111.00	127.66
4	D	602	HAS	C21-C22-C23	-6.92	111.01	127.66
4	A	601	HAS	CHD-C4C-C3C	-6.86	120.61	129.61
4	D	602	HAS	CHD-C4C-C3C	-6.83	120.66	129.61
7	D	605	DLX	C20-C19-C18	-6.75	107.46	121.12
7	A	604	DLX	C20-C19-C18	-6.72	107.51	121.12
7	D	605	DLX	C17-C18-C19	-6.72	111.48	127.66
7	A	604	DLX	C17-C18-C19	-6.71	111.50	127.66
4	A	601	HAS	C16-C15-C14	-6.68	107.59	121.12
4	D	602	HAS	C16-C15-C14	-6.67	107.63	121.12
7	A	604	DLX	C25-C26-C27	-6.59	111.79	127.66
7	D	605	DLX	C25-C26-C27	-6.58	111.81	127.66
4	D	602	HAS	C24-C23-C22	-6.49	107.98	121.12
4	A	601	HAS	C24-C23-C22	-6.47	108.02	121.12
7	A	604	DLX	C21-C22-C23	-6.46	112.10	127.66
7	D	605	DLX	C21-C22-C23	-6.45	112.13	127.66
4	D	602	HAS	CHD-C4A-C3A	-6.40	118.83	129.53
4	A	601	HAS	CHD-C4A-C3A	-6.39	118.84	129.53
4	A	601	HAS	C13-C14-C15	-6.22	112.69	127.66
4	D	602	HAS	C13-C14-C15	-6.20	112.72	127.66
4	A	601	HAS	CHC-C1C-C2C	-6.04	118.99	129.45

Continued on next page...

CONFIDENTIAL VALIDATION REPORT

Continued from previous page...

Mol	Chain	Res	Type	Atoms	Z	Observed(°)	Ideal(°)
4	D	602	HAS	CHC-C1C-C2C	-6.04	119.00	129.45
7	A	604	DLX	C46-C19-C18	-5.90	108.54	123.68
7	D	605	DLX	C46-C19-C18	-5.89	108.56	123.68
4	D	602	HAS	C20-C19-C18	-5.86	109.25	121.12
4	A	601	HAS	C20-C19-C18	-5.86	109.27	121.12
7	D	605	DLX	C29-C30-C31	-5.82	113.64	127.66
7	A	604	DLX	C29-C30-C31	-5.81	113.67	127.66
7	D	605	DLX	C24-C23-C22	-5.72	109.55	121.12
7	A	604	DLX	C24-C23-C22	-5.70	109.58	121.12
7	A	604	DLX	C44-C27-C26	-5.34	109.97	123.68
7	D	605	DLX	C44-C27-C26	-5.34	109.99	123.68
4	D	602	HAS	C27-C19-C18	-5.28	110.12	123.68
5	A	602	HEM	CAD-CBD-CGD	-5.28	103.82	112.67
4	A	601	HAS	C27-C19-C18	-5.27	110.15	123.68
5	D	603	HEM	CAD-CBD-CGD	-5.27	103.83	112.67
7	A	604	DLX	C43-C31-C30	-5.05	110.72	123.68
7	D	605	DLX	C43-C31-C30	-5.05	110.72	123.68
8	A	607	PGV	O01-C1-C2	5.00	122.27	111.50
8	D	601	PGV	O01-C1-C2	5.00	122.27	111.50
4	A	601	HAS	C26-C15-C14	-4.79	111.38	123.68
4	D	602	HAS	C26-C15-C14	-4.79	111.39	123.68
7	A	604	DLX	C32-C31-C30	-4.78	111.44	121.12
7	D	605	DLX	C32-C31-C30	-4.77	111.47	121.12
7	D	605	DLX	C45-C23-C22	-4.74	111.52	123.68
7	A	604	DLX	C45-C23-C22	-4.73	111.53	123.68
8	C	101	PGV	O01-C1-C2	4.55	121.32	111.50
8	F	101	PGV	O01-C1-C2	4.55	121.30	111.50
8	D	606	PGV	O01-C1-C2	4.51	121.23	111.50
8	A	605	PGV	O01-C1-C2	4.51	121.23	111.50
7	A	604	DLX	C47-C15-C14	-4.43	112.31	123.68
7	D	605	DLX	C47-C15-C14	-4.41	112.36	123.68
5	D	603	HEM	CAA-CBA-CGA	-4.41	105.27	112.67
5	A	602	HEM	CAA-CBA-CGA	-4.39	105.30	112.67
4	A	601	HAS	OMD-CMD-C2D	-4.26	119.00	124.39
4	D	602	HAS	OMD-CMD-C2D	-4.20	119.07	124.39
4	A	601	HAS	C25-C23-C22	-4.16	113.00	123.68
4	D	602	HAS	C25-C23-C22	-4.15	113.03	123.68
4	D	602	HAS	C32-C30-C29	-4.04	110.98	122.65
4	A	601	HAS	C32-C30-C29	-4.03	111.01	122.65
4	A	601	HAS	C12-C11-C3B	-4.01	108.92	114.11
4	D	602	HAS	C12-C11-C3B	-3.99	108.96	114.11
4	D	602	HAS	C28-C29-C30	-3.69	115.13	127.75

Continued on next page...

CONFIDENTIAL VALIDATION REPORT

Continued from previous page...

Mol	Chain	Res	Type	Atoms	Z	Observed(°)	Ideal(°)
4	A	601	HAS	C28-C29-C30	-3.69	115.13	127.75
4	D	602	HAS	CMC-C2C-C3C	3.64	131.49	124.68
4	A	601	HAS	CMC-C2C-C3C	3.62	131.45	124.68
4	D	602	HAS	CMA-C3A-C2A	3.31	131.18	124.94
7	D	605	DLX	C28-C27-C26	-3.31	114.43	121.12
7	A	604	DLX	C28-C27-C26	-3.30	114.43	121.12
4	A	601	HAS	CMA-C3A-C2A	3.30	131.17	124.94
4	D	602	HAS	CAD-CBD-CGD	-3.19	107.31	112.67
4	A	601	HAS	CAD-CBD-CGD	-3.17	107.36	112.67
5	A	602	HEM	CBA-CAA-C2A	-3.13	106.72	112.49
5	D	603	HEM	CBA-CAA-C2A	-3.13	106.72	112.49
4	A	601	HAS	C26-C15-C16	-3.11	110.05	115.27
4	D	602	HAS	C26-C15-C16	-3.08	110.08	115.27
8	A	605	PGV	O03-C19-C20	3.05	121.49	111.91
8	D	606	PGV	O03-C19-C20	3.05	121.47	111.91
4	D	602	HAS	C31-C30-C29	-2.92	114.20	122.65
4	A	601	HAS	C31-C30-C29	-2.92	114.21	122.65
8	F	101	PGV	O03-C19-C20	2.89	120.96	111.91
8	C	101	PGV	O03-C19-C20	2.88	120.96	111.91
8	A	607	PGV	O03-C19-C20	2.83	120.78	111.91
8	D	601	PGV	O03-C19-C20	2.83	120.78	111.91
4	D	602	HAS	C4C-C3C-C2C	2.82	108.74	104.41
4	A	601	HAS	C4C-C3C-C2C	2.80	108.71	104.41
7	D	605	DLX	C45-C23-C24	-2.79	110.58	115.27
7	A	604	DLX	C45-C23-C24	-2.77	110.61	115.27
4	A	601	HAS	C13-C12-C11	-2.75	110.22	114.35
4	D	602	HAS	C13-C12-C11	-2.72	110.27	114.35
4	A	601	HAS	CAA-C2A-C3A	2.62	134.77	127.25
4	D	602	HAS	CAA-C2A-C3A	2.61	134.76	127.25
7	D	605	DLX	C43-C31-C32	-2.46	111.13	115.27
7	A	604	DLX	C43-C31-C32	-2.46	111.13	115.27
8	C	101	PGV	C02-O01-C1	-2.44	111.78	117.79
8	F	101	PGV	C02-O01-C1	-2.44	111.78	117.79
4	A	601	HAS	C32-C30-C31	-2.43	109.25	114.60
8	D	601	PGV	C21-C20-C19	-2.42	104.82	113.62
8	A	607	PGV	C21-C20-C19	-2.41	104.84	113.62
4	D	602	HAS	C32-C30-C31	-2.40	109.29	114.60
5	D	603	HEM	CMC-C2C-C3C	2.39	129.15	124.68
5	A	602	HEM	CMC-C2C-C3C	2.38	129.13	124.68
7	A	604	DLX	C03-C04-C05	2.37	119.83	116.62
7	D	605	DLX	C03-C04-C05	2.36	119.83	116.62
7	D	605	DLX	O12-C04-C05	-2.35	119.46	121.96

Continued on next page...

CONFIDENTIAL VALIDATION REPORT

Continued from previous page...

Mol	Chain	Res	Type	Atoms	Z	Observed(°)	Ideal(°)
8	A	605	PGV	O03-C19-O04	-2.31	117.75	123.59
7	A	604	DLX	O12-C04-C05	-2.30	119.50	121.96
8	D	606	PGV	O03-C19-O04	-2.30	117.79	123.59
7	A	604	DLX	C13-C05-C04	2.29	118.74	116.88
7	D	605	DLX	C13-C05-C04	2.28	118.73	116.88
8	A	605	PGV	C02-O01-C1	-2.28	112.19	117.79
8	D	606	PGV	C02-O01-C1	-2.26	112.23	117.79
4	D	602	HAS	CBA-CAA-C2A	2.24	116.61	112.49
4	A	601	HAS	CBA-CAA-C2A	2.24	116.61	112.49
8	D	606	PGV	O01-C1-O02	-2.22	118.33	123.70
8	A	605	PGV	O01-C1-O02	-2.20	118.37	123.70
5	A	602	HEM	CBD-CAD-C3D	-2.20	108.42	112.48
5	D	603	HEM	CBD-CAD-C3D	-2.19	108.44	112.48
7	D	605	DLX	C47-C15-C16	-2.16	111.64	115.27
7	A	604	DLX	C47-C15-C16	-2.15	111.65	115.27
4	D	602	HAS	C25-C23-C24	-2.14	111.68	115.27
4	A	601	HAS	C25-C23-C24	-2.14	111.68	115.27
7	A	604	DLX	C13-C05-C06	-2.10	120.53	123.30
7	D	605	DLX	C13-C05-C06	-2.09	120.56	123.30
8	C	101	PGV	O01-C1-O02	-2.05	118.74	123.70
8	F	101	PGV	O01-C1-O02	-2.05	118.75	123.70

There are no chirality outliers.

All (282) torsion outliers are listed below:

Mol	Chain	Res	Type	Atoms
8	D	606	PGV	C03-O11-P-O13
8	D	606	PGV	C03-O11-P-O14
8	D	606	PGV	C04-O12-P-O13
8	D	606	PGV	O02-C1-O01-C02
8	D	606	PGV	C2-C1-O01-C02
7	A	604	DLX	C29-C30-C31-C32
7	A	604	DLX	C25-C26-C27-C28
7	A	604	DLX	C21-C22-C23-C45
7	A	604	DLX	C21-C22-C23-C24
7	A	604	DLX	C17-C18-C19-C46
7	A	604	DLX	C13-C14-C15-C16
8	C	101	PGV	C04-O12-P-O13
4	D	602	HAS	C1A-C2A-CAA-CBA
4	D	602	HAS	C3A-C2A-CAA-CBA
4	D	602	HAS	C1D-C2D-CMD-OMD
4	D	602	HAS	C3D-C2D-CMD-OMD

Continued on next page...

CONFIDENTIAL VALIDATION REPORT

Continued from previous page...

Mol	Chain	Res	Type	Atoms
4	D	602	HAS	C2D-C3D-CAD-CBD
4	D	602	HAS	C13-C14-C15-C26
4	D	602	HAS	C14-C15-C16-C17
4	D	602	HAS	C17-C18-C19-C20
4	D	602	HAS	C18-C19-C20-C21
4	D	602	HAS	C19-C20-C21-C22
4	D	602	HAS	C21-C22-C23-C24
4	D	602	HAS	C28-C29-C30-C32
8	A	607	PGV	C04-O12-P-O11
8	A	607	PGV	C04-O12-P-O13
8	F	101	PGV	C04-O12-P-O13
4	A	601	HAS	C1A-C2A-CAA-CBA
4	A	601	HAS	C3A-C2A-CAA-CBA
4	A	601	HAS	C1D-C2D-CMD-OMD
4	A	601	HAS	C3D-C2D-CMD-OMD
4	A	601	HAS	C2D-C3D-CAD-CBD
4	A	601	HAS	C13-C14-C15-C26
4	A	601	HAS	C14-C15-C16-C17
4	A	601	HAS	C17-C18-C19-C20
4	A	601	HAS	C18-C19-C20-C21
4	A	601	HAS	C19-C20-C21-C22
4	A	601	HAS	C21-C22-C23-C24
4	A	601	HAS	C28-C29-C30-C32
8	A	605	PGV	C03-O11-P-O13
8	A	605	PGV	C03-O11-P-O14
8	A	605	PGV	C04-O12-P-O13
8	A	605	PGV	O02-C1-O01-C02
8	A	605	PGV	C2-C1-O01-C02
8	D	601	PGV	C04-O12-P-O11
8	D	601	PGV	C04-O12-P-O13
9	D	607	3PE	C1-O11-P-O12
9	D	607	3PE	C11-O13-P-O11
9	D	607	3PE	C11-O13-P-O14
9	D	607	3PE	O13-C11-C12-N
9	D	607	3PE	C22-C21-O21-C2
9	A	606	3PE	C1-O11-P-O12
9	A	606	3PE	C11-O13-P-O11
9	A	606	3PE	C11-O13-P-O14
9	A	606	3PE	O13-C11-C12-N
9	A	606	3PE	C22-C21-O21-C2
7	D	605	DLX	C29-C30-C31-C32
7	D	605	DLX	C25-C26-C27-C28

Continued on next page...

CONFIDENTIAL VALIDATION REPORT

Continued from previous page...

Mol	Chain	Res	Type	Atoms
7	D	605	DLX	C21-C22-C23-C45
7	D	605	DLX	C21-C22-C23-C24
7	D	605	DLX	C17-C18-C19-C46
7	D	605	DLX	C13-C14-C15-C16
9	D	607	3PE	O32-C31-O31-C3
9	A	606	3PE	O32-C31-O31-C3
9	D	607	3PE	C32-C31-O31-C3
9	A	606	3PE	C32-C31-O31-C3
8	C	101	PGV	O04-C19-O03-C01
8	F	101	PGV	O04-C19-O03-C01
9	D	607	3PE	O22-C21-O21-C2
9	A	606	3PE	O22-C21-O21-C2
8	C	101	PGV	C20-C19-O03-C01
8	F	101	PGV	C20-C19-O03-C01
4	D	602	HAS	C27-C19-C20-C21
4	A	601	HAS	C27-C19-C20-C21
7	A	604	DLX	C22-C23-C24-C25
7	D	605	DLX	C22-C23-C24-C25
8	A	607	PGV	O04-C19-O03-C01
8	D	601	PGV	O04-C19-O03-C01
7	A	604	DLX	C29-C30-C31-C43
4	D	602	HAS	C21-C22-C23-C25
4	A	601	HAS	C21-C22-C23-C25
7	D	605	DLX	C29-C30-C31-C43
7	A	604	DLX	C17-C18-C19-C20
7	D	605	DLX	C17-C18-C19-C20
8	A	607	PGV	C20-C19-O03-C01
8	D	601	PGV	C20-C19-O03-C01
7	A	604	DLX	C43-C31-C32-C33
7	A	604	DLX	C46-C19-C20-C21
7	D	605	DLX	C43-C31-C32-C33
7	D	605	DLX	C46-C19-C20-C21
4	D	602	HAS	C22-C23-C24-C28
4	A	601	HAS	C22-C23-C24-C28
4	D	602	HAS	C28-C29-C30-C31
4	A	601	HAS	C28-C29-C30-C31
7	A	604	DLX	C42-C35-C36-C37
7	D	605	DLX	C42-C35-C36-C37
8	A	607	PGV	C19-C20-C21-C22
8	D	601	PGV	C19-C20-C21-C22
7	A	604	DLX	C25-C26-C27-C44
7	D	605	DLX	C25-C26-C27-C44

Continued on next page...

CONFIDENTIAL VALIDATION REPORT

Continued from previous page...

Mol	Chain	Res	Type	Atoms
9	D	607	3PE	C21-C22-C23-C24
9	A	606	3PE	C21-C22-C23-C24
9	D	607	3PE	C31-C32-C33-C34
9	A	606	3PE	C31-C32-C33-C34
4	D	602	HAS	C23-C24-C28-C29
4	A	601	HAS	C23-C24-C28-C29
8	A	607	PGV	O12-C04-C05-O05
8	D	601	PGV	O12-C04-C05-O05
7	A	604	DLX	C35-C36-C37-C38
7	A	604	DLX	C32-C33-C34-C35
7	D	605	DLX	C35-C36-C37-C38
7	D	605	DLX	C32-C33-C34-C35
8	D	606	PGV	C03-O11-P-O12
8	A	605	PGV	C03-O11-P-O12
8	C	101	PGV	C7-C8-C9-C10
8	F	101	PGV	C7-C8-C9-C10
9	D	607	3PE	C28-C29-C2A-C2B
9	A	606	3PE	C28-C29-C2A-C2B
8	A	607	PGV	O12-C04-C05-C06
8	D	601	PGV	O12-C04-C05-C06
9	D	607	3PE	C22-C23-C24-C25
9	A	606	3PE	C22-C23-C24-C25
8	D	606	PGV	C4-C5-C6-C7
8	A	605	PGV	C4-C5-C6-C7
8	A	607	PGV	C28-C29-C30-C31
8	D	601	PGV	C28-C29-C30-C31
8	A	607	PGV	C30-C31-C32-C33
8	D	601	PGV	C30-C31-C32-C33
8	A	605	PGV	C3-C4-C5-C6
8	D	606	PGV	C3-C4-C5-C6
8	A	607	PGV	C13-C14-C15-C16
8	D	601	PGV	C13-C14-C15-C16
8	D	606	PGV	C7-C8-C9-C10
8	A	605	PGV	C7-C8-C9-C10
8	D	601	PGV	C24-C25-C26-C27
9	D	607	3PE	C27-C28-C29-C2A
9	A	606	3PE	C27-C28-C29-C2A
8	A	607	PGV	C24-C25-C26-C27
8	A	607	PGV	C04-C05-C06-O06
8	D	601	PGV	C04-C05-C06-O06
9	D	607	3PE	C38-C39-C3A-C3B
9	A	606	3PE	C38-C39-C3A-C3B

Continued on next page...

CONFIDENTIAL VALIDATION REPORT

Continued from previous page...

Mol	Chain	Res	Type	Atoms
9	D	607	3PE	C36-C37-C38-C39
9	A	606	3PE	C36-C37-C38-C39
8	C	101	PGV	C28-C29-C30-C31
8	F	101	PGV	C28-C29-C30-C31
7	A	604	DLX	C44-C27-C28-C29
7	D	605	DLX	C44-C27-C28-C29
8	A	607	PGV	O05-C05-C06-O06
8	D	601	PGV	O05-C05-C06-O06
9	D	607	3PE	C33-C34-C35-C36
9	A	606	3PE	C33-C34-C35-C36
7	A	604	DLX	C37-C38-C39-C40
7	D	605	DLX	C37-C38-C39-C40
9	D	607	3PE	C39-C3A-C3B-C3C
9	A	606	3PE	C39-C3A-C3B-C3C
8	C	101	PGV	C19-C20-C21-C22
8	F	101	PGV	C19-C20-C21-C22
7	A	604	DLX	C26-C27-C28-C29
7	D	605	DLX	C26-C27-C28-C29
8	D	601	PGV	C25-C26-C27-C28
8	A	607	PGV	C25-C26-C27-C28
9	A	606	3PE	C37-C38-C39-C3A
9	D	607	3PE	C37-C38-C39-C3A
7	A	604	DLX	C27-C28-C29-C30
7	D	605	DLX	C27-C28-C29-C30
8	A	605	PGV	O01-C02-C03-O11
7	A	604	DLX	C04-C05-C13-C14
7	D	605	DLX	C04-C05-C13-C14
8	A	607	PGV	C15-C16-C17-C18
8	D	601	PGV	C15-C16-C17-C18
7	A	604	DLX	C19-C20-C21-C22
7	D	605	DLX	C19-C20-C21-C22
8	D	606	PGV	O01-C02-C03-O11
8	F	101	PGV	C12-C13-C14-C15
9	D	607	3PE	C35-C36-C37-C38
9	D	607	3PE	C2C-C2D-C2E-C2F
9	A	606	3PE	C35-C36-C37-C38
9	A	606	3PE	C2C-C2D-C2E-C2F
7	A	604	DLX	C34-C35-C36-C37
7	D	605	DLX	C34-C35-C36-C37
8	D	601	PGV	C5-C6-C7-C8
9	D	607	3PE	C2A-C2B-C2C-C2D
9	A	606	3PE	C2A-C2B-C2C-C2D

Continued on next page...

CONFIDENTIAL VALIDATION REPORT

Continued from previous page...

Mol	Chain	Res	Type	Atoms
8	A	607	PGV	C5-C6-C7-C8
8	C	101	PGV	C12-C13-C14-C15
8	C	101	PGV	C01-C02-C03-O11
8	F	101	PGV	C01-C02-C03-O11
8	D	601	PGV	C1-C2-C3-C4
8	A	607	PGV	C1-C2-C3-C4
8	A	607	PGV	C2-C1-O01-C02
8	A	607	PGV	C9-C10-C11-C12
8	D	601	PGV	C9-C10-C11-C12
8	C	101	PGV	O03-C01-C02-C03
8	A	607	PGV	O03-C01-C02-C03
8	F	101	PGV	O03-C01-C02-C03
8	D	601	PGV	O03-C01-C02-C03
8	D	601	PGV	C2-C1-O01-C02
8	C	101	PGV	C2-C3-C4-C5
8	F	101	PGV	C2-C3-C4-C5
8	C	101	PGV	C4-C5-C6-C7
8	A	607	PGV	O01-C02-C03-O11
8	D	601	PGV	O01-C02-C03-O11
8	F	101	PGV	C4-C5-C6-C7
9	D	607	3PE	C3E-C3F-C3G-C3H
9	A	606	3PE	C3E-C3F-C3G-C3H
8	A	607	PGV	O02-C1-O01-C02
8	D	601	PGV	O02-C1-O01-C02
7	A	604	DLX	C37-C38-C39-C41
7	D	605	DLX	C37-C38-C39-C41
8	D	606	PGV	C9-C10-C11-C12
8	A	605	PGV	C9-C10-C11-C12
8	C	101	PGV	C5-C6-C7-C8
8	C	101	PGV	C24-C25-C26-C27
8	F	101	PGV	C5-C6-C7-C8
8	F	101	PGV	C24-C25-C26-C27
8	A	607	PGV	C3-C4-C5-C6
8	D	601	PGV	C3-C4-C5-C6
8	A	607	PGV	C01-C02-C03-O11
8	D	601	PGV	C01-C02-C03-O11
9	D	607	3PE	C2-C1-O11-P
9	A	606	3PE	C2-C1-O11-P
8	C	101	PGV	C04-O12-P-O11
8	F	101	PGV	C04-O12-P-O11
8	D	606	PGV	C01-C02-C03-O11
8	A	605	PGV	C01-C02-C03-O11

Continued on next page...

CONFIDENTIAL VALIDATION REPORT

Continued from previous page...

Mol	Chain	Res	Type	Atoms
8	D	606	PGV	C11-C10-C9-C8
8	A	605	PGV	C11-C10-C9-C8
9	A	606	3PE	C24-C25-C26-C27
9	D	607	3PE	C24-C25-C26-C27
8	C	101	PGV	O01-C02-C03-O11
8	F	101	PGV	O01-C02-C03-O11
4	D	602	HAS	C4D-C3D-CAD-CBD
4	A	601	HAS	C4D-C3D-CAD-CBD
8	A	607	PGV	O03-C01-C02-O01
8	D	601	PGV	O03-C01-C02-O01
8	D	606	PGV	C11-C12-C13-C14
8	A	605	PGV	C11-C12-C13-C14
8	D	606	PGV	C25-C26-C27-C28
8	A	605	PGV	C25-C26-C27-C28
4	D	602	HAS	C15-C16-C17-C18
4	A	601	HAS	C15-C16-C17-C18
7	A	604	DLX	C36-C37-C38-C39
7	D	605	DLX	C36-C37-C38-C39
4	D	602	HAS	C11-C12-C13-C14
4	A	601	HAS	C11-C12-C13-C14
8	D	606	PGV	C04-O12-P-O11
8	C	101	PGV	C03-O11-P-O12
8	F	101	PGV	C03-O11-P-O12
8	A	605	PGV	C04-O12-P-O11
7	A	604	DLX	C47-C15-C16-C17
7	D	605	DLX	C47-C15-C16-C17
8	A	607	PGV	C11-C12-C13-C14
8	D	601	PGV	C11-C12-C13-C14
9	D	607	3PE	C26-C27-C28-C29
9	A	606	3PE	C26-C27-C28-C29
8	C	101	PGV	O03-C01-C02-O01
8	F	101	PGV	O03-C01-C02-O01
8	C	101	PGV	C26-C27-C28-C29
8	F	101	PGV	C26-C27-C28-C29
8	C	101	PGV	C20-C21-C22-C23
8	F	101	PGV	C20-C21-C22-C23
9	D	607	3PE	C1-O11-P-O13
9	A	606	3PE	C1-O11-P-O13
8	C	101	PGV	C9-C10-C11-C12
8	C	101	PGV	C11-C12-C13-C14
8	F	101	PGV	C9-C10-C11-C12
8	F	101	PGV	C11-C12-C13-C14

Continued on next page...

CONFIDENTIAL VALIDATION REPORT

Continued from previous page...

Mol	Chain	Res	Type	Atoms
9	D	607	3PE	C29-C2A-C2B-C2C
9	A	606	3PE	C29-C2A-C2B-C2C
7	A	604	DLX	C28-C29-C30-C31
7	D	605	DLX	C28-C29-C30-C31
9	D	607	3PE	C1-O11-P-O14
9	A	606	3PE	C1-O11-P-O14
8	A	607	PGV	C03-C02-O01-C1
8	D	601	PGV	C03-C02-O01-C1
7	A	604	DLX	C06-C05-C13-C14
7	D	605	DLX	C06-C05-C13-C14
7	A	604	DLX	C23-C24-C25-C26
7	D	605	DLX	C23-C24-C25-C26
8	A	607	PGV	O03-C19-C20-C21
8	D	601	PGV	O03-C19-C20-C21

There are no ring outliers.

14 monomers are involved in 133 short contacts:

Mol	Chain	Res	Type	Clashes	Symm-Clashes
8	D	606	PGV	2	0
5	A	602	HEM	8	0
8	C	101	PGV	4	0
8	A	607	PGV	27	0
4	D	602	HAS	11	0
7	A	604	DLX	3	0
8	F	101	PGV	4	0
4	A	601	HAS	11	0
8	A	605	PGV	3	0
5	D	603	HEM	8	0
8	D	601	PGV	27	0
9	D	607	3PE	34	0
9	A	606	3PE	36	0
7	D	605	DLX	3	0

The following is a two-dimensional graphical depiction of Mogul quality analysis of bond lengths, bond angles, torsion angles, and ring geometry for all instances of the Ligand of Interest. In addition, ligands with molecular weight > 250 and outliers as shown on the validation Tables will also be included. For torsion angles, if less then 5% of the Mogul distribution of torsion angles is within 10 degrees of the torsion angle in question, then that torsion angle is considered an outlier. Any bond that is central to one or more torsion angles identified as an outlier by Mogul will be highlighted in the graph. For rings, the root-mean-square deviation (RMSD) between the ring in question and similar rings identified by Mogul is calculated over all ring torsion angles. If the

CONFIDENTIAL VALIDATION REPORT

average RMSD is greater than 60 degrees and the minimal RMSD between the ring in question and any Mogul-identified rings is also greater than 60 degrees, then that ring is considered an outlier. The outliers are highlighted in purple. The color gray indicates Mogul did not find sufficient equivalents in the CSD to analyse the geometry.

Ligand PGV D 606

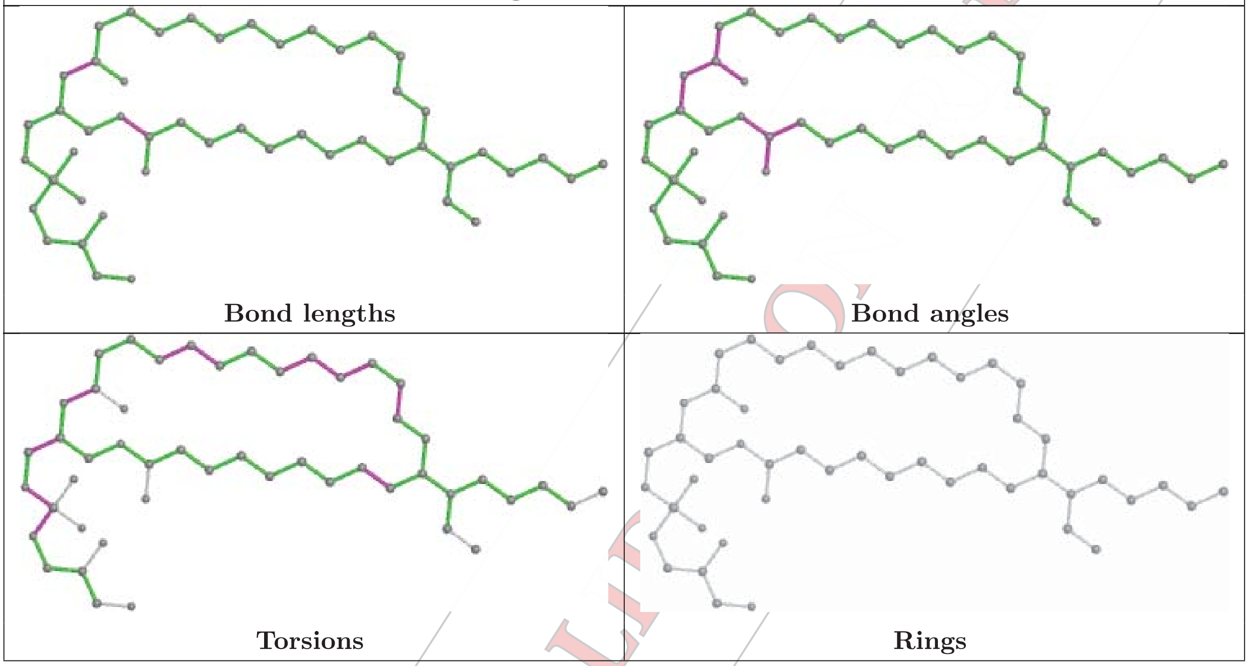

CONFIDENTIAL VALIDATION REPORT

Ligand HEM A 602

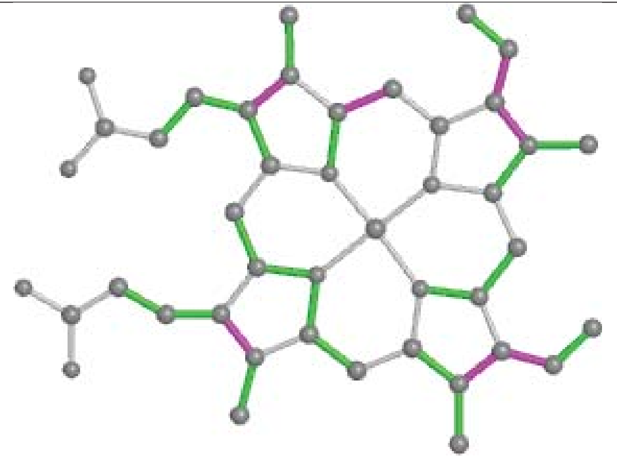

Bond lengths

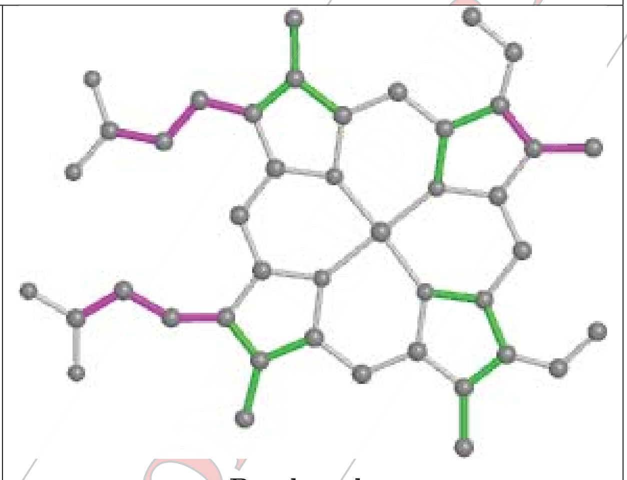

Bond angles

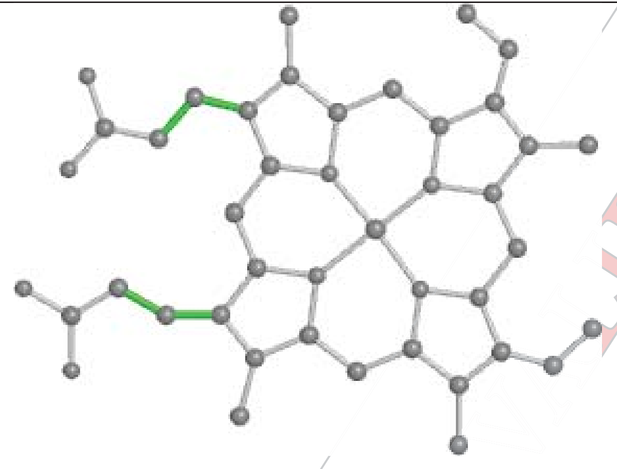

Torsions

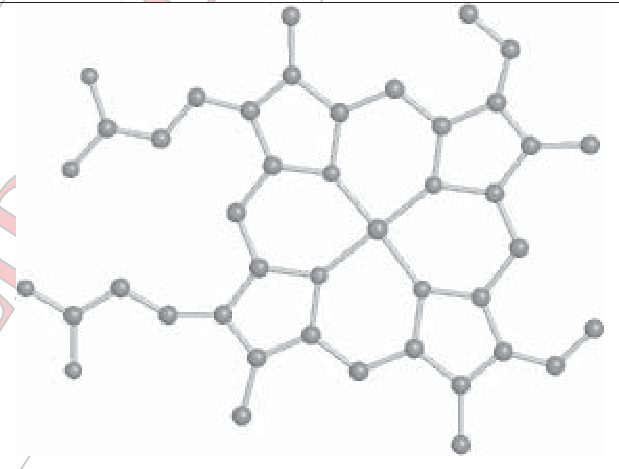

Rings

Ligand PGV C 101

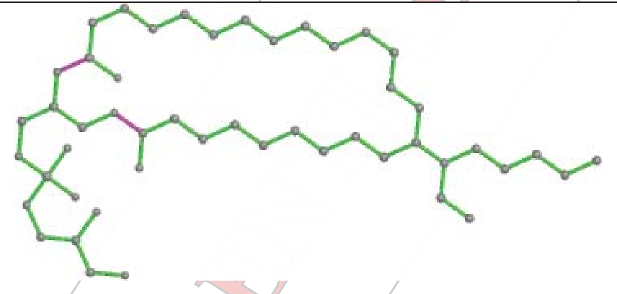

Bond lengths

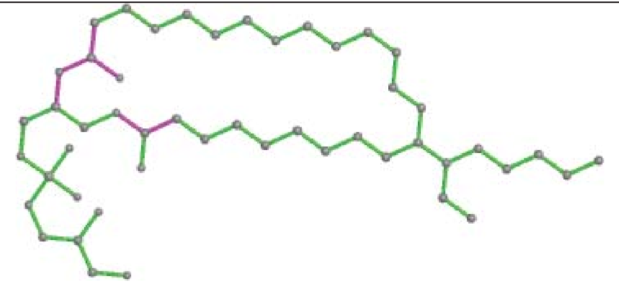

Bond angles

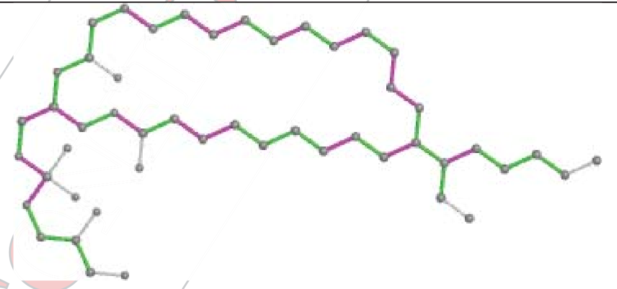

Torsions

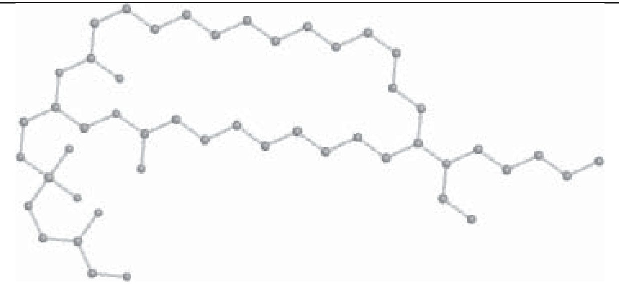

Rings

Ligand PGV A 607

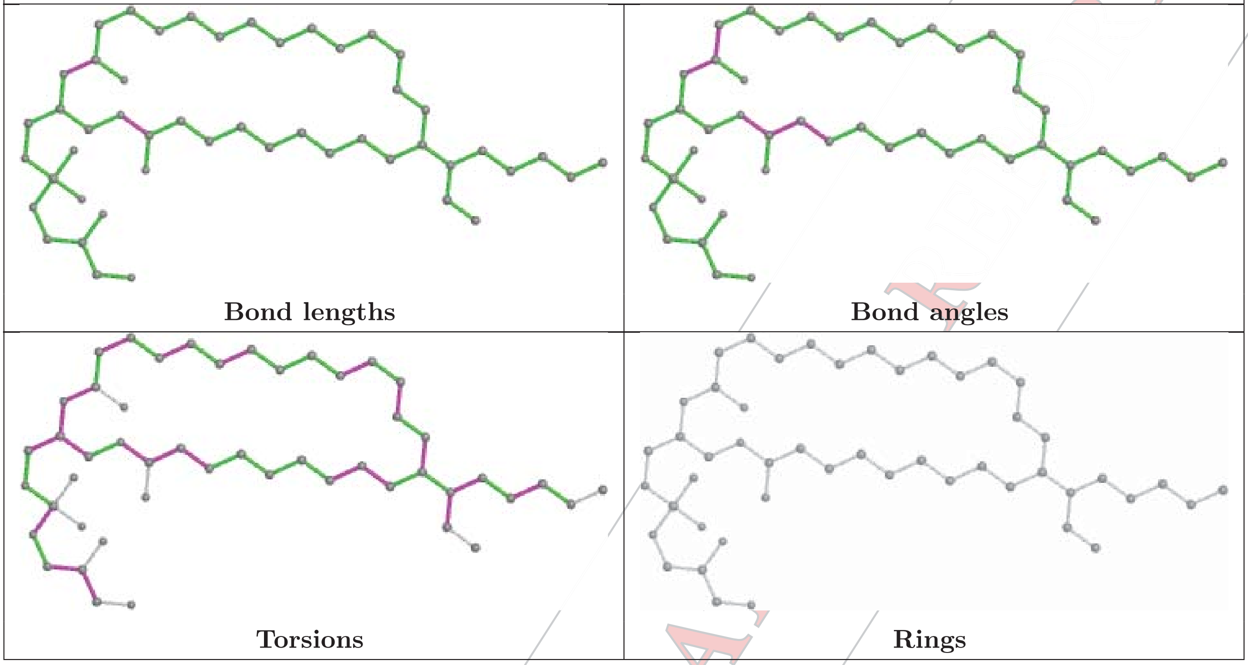

Ligand HAS D 602

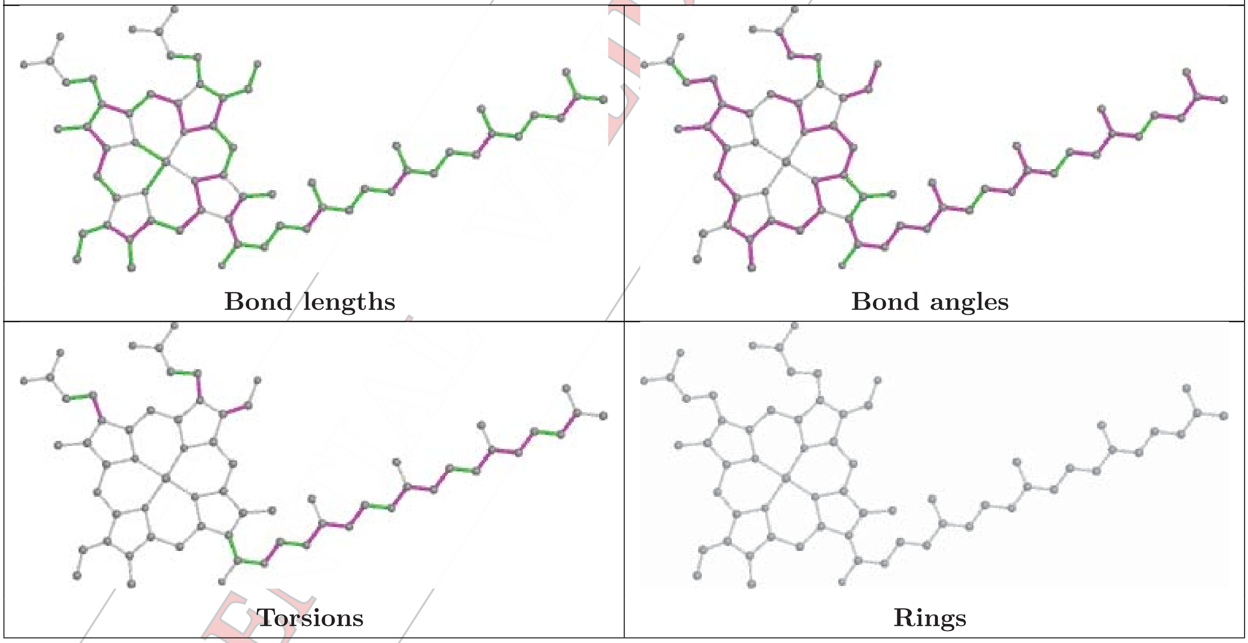

CONFIDENTIAL REPORT

Ligand DLX A 604

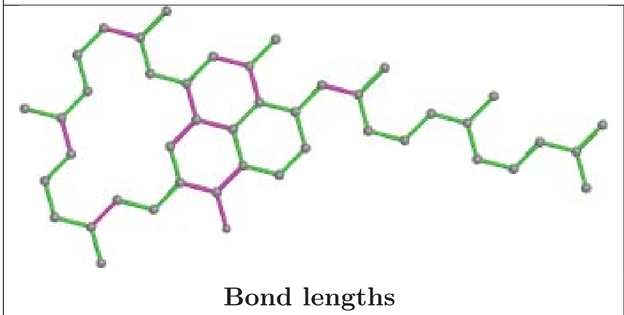

Bond lengths

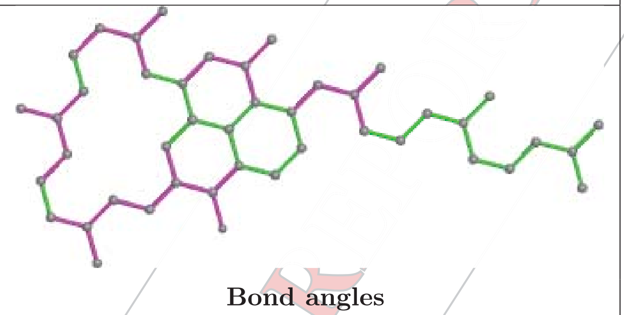

Bond angles

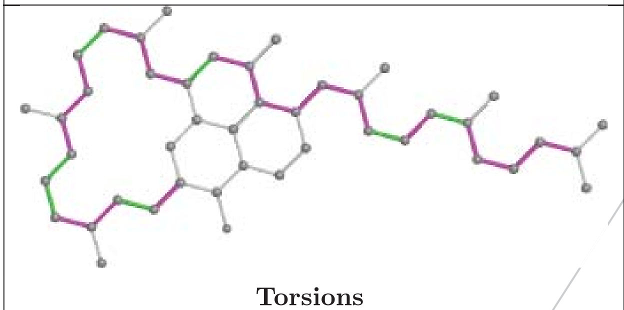

Torsions

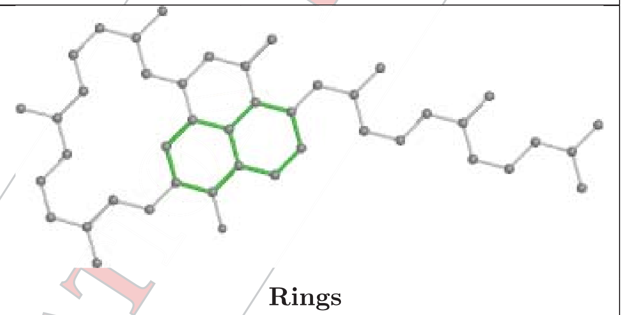

Rings

Ligand PGV F 101

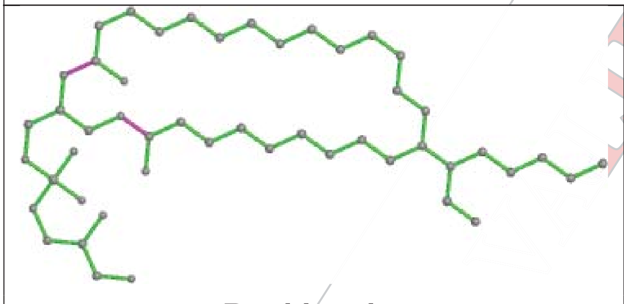

Bond lengths

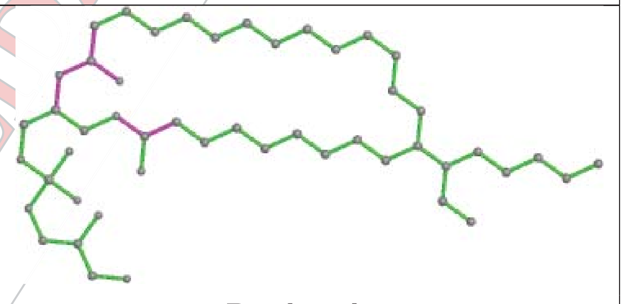

Bond angles

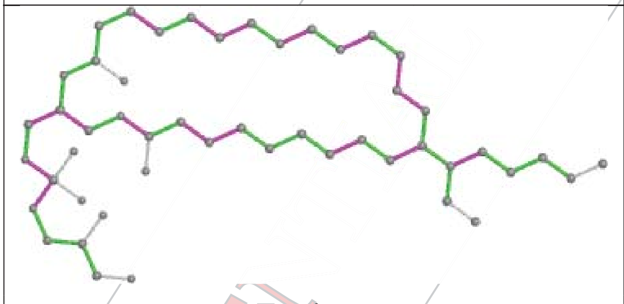

Torsions

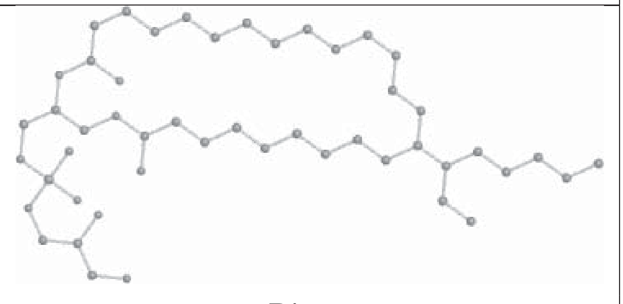

Rings

CONFIDENTIAL VALIDATION REPORT

Ligand HAS A 601

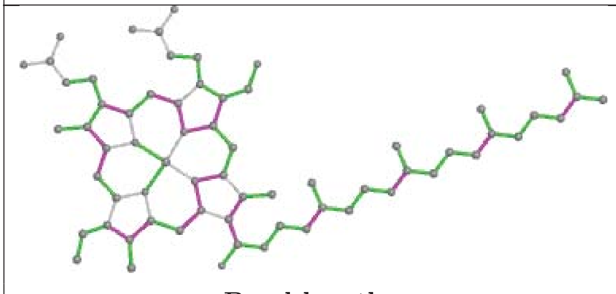
Bond lengths

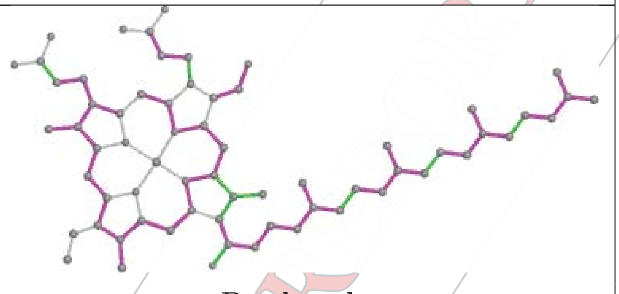
Bond angles

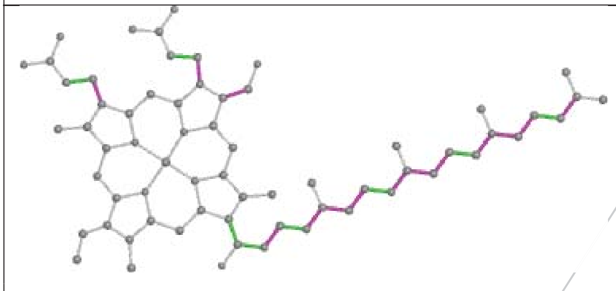
Torsions

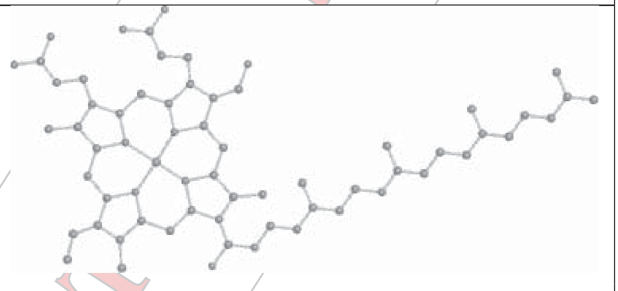
Rings

Ligand PGV A 605

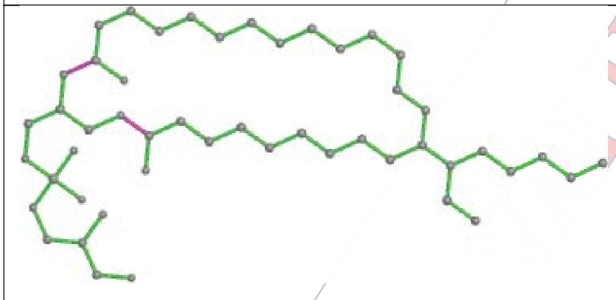
Bond lengths

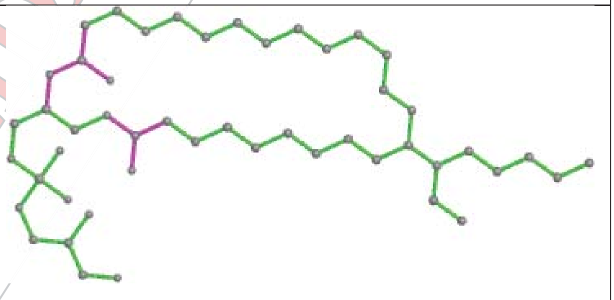
Bond angles

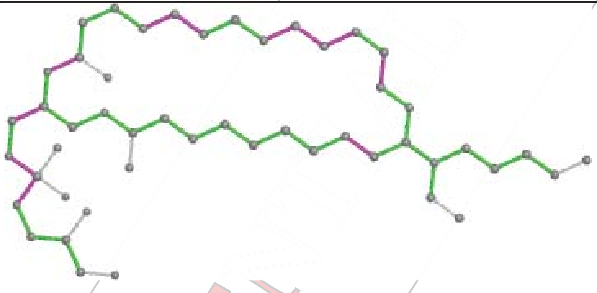
Torsions

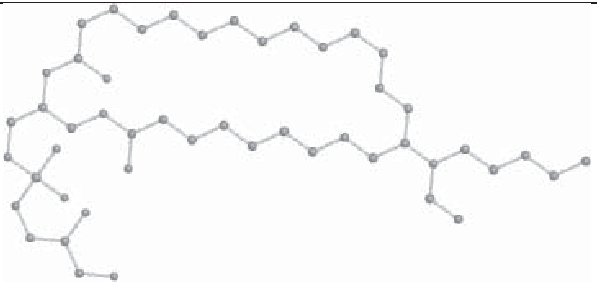
Rings

Ligand HEM D 603

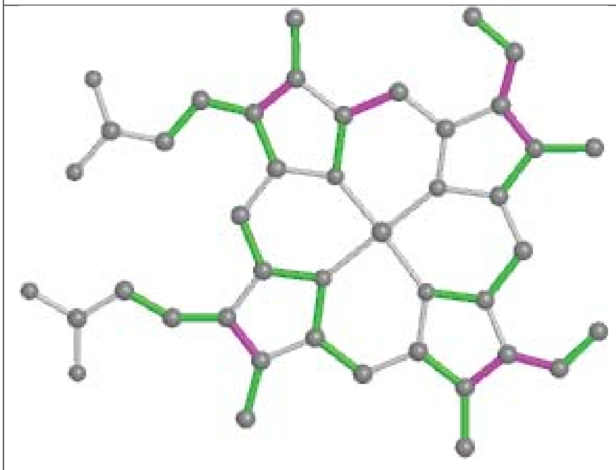

Bond lengths

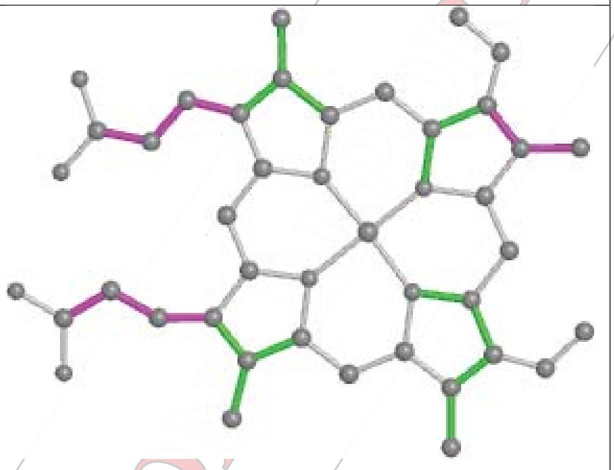

Bond angles

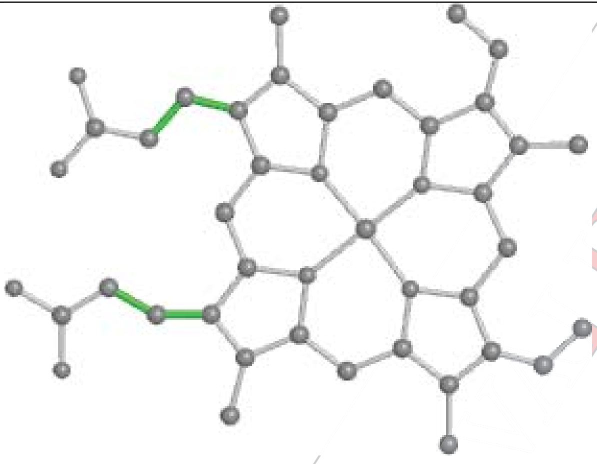

Torsions

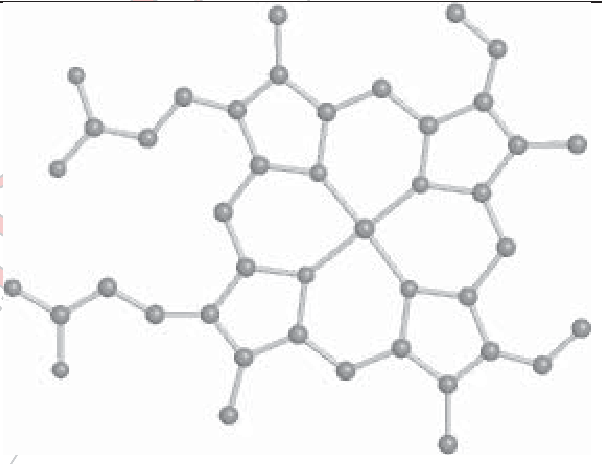

Rings

Ligand PGV D 601

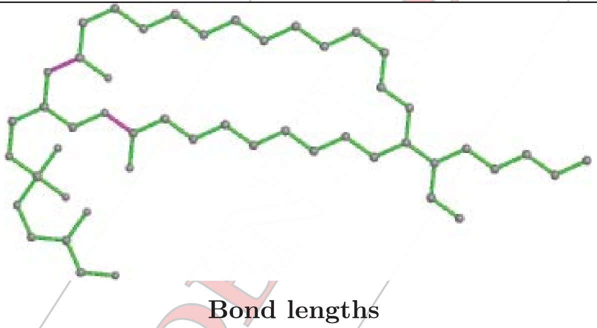

Bond lengths

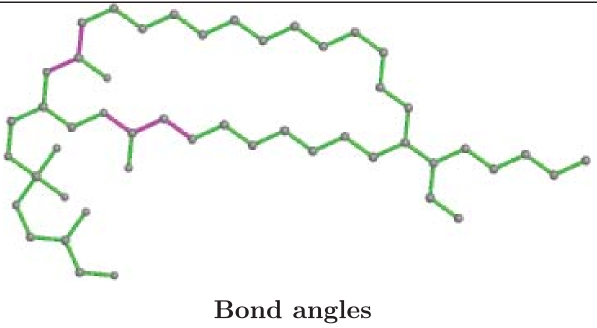

Bond angles

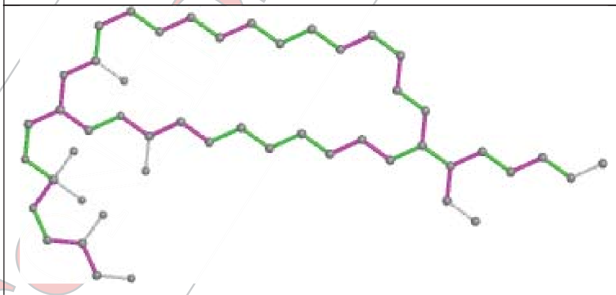

Torsions

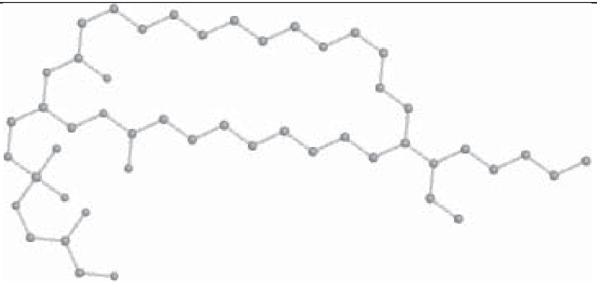

Rings

Ligand 3PE D 607

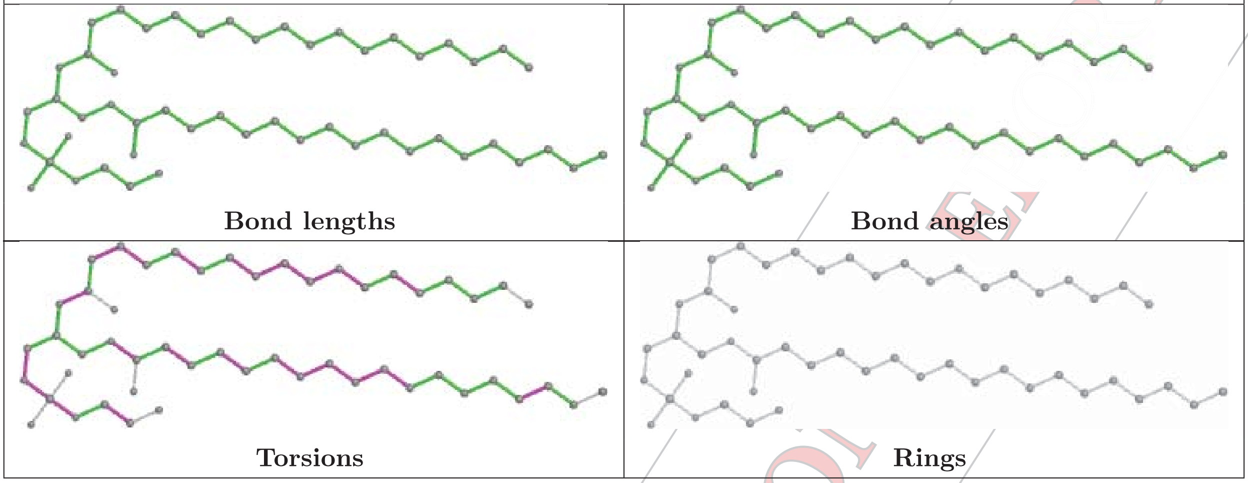

Ligand 3PE A 606

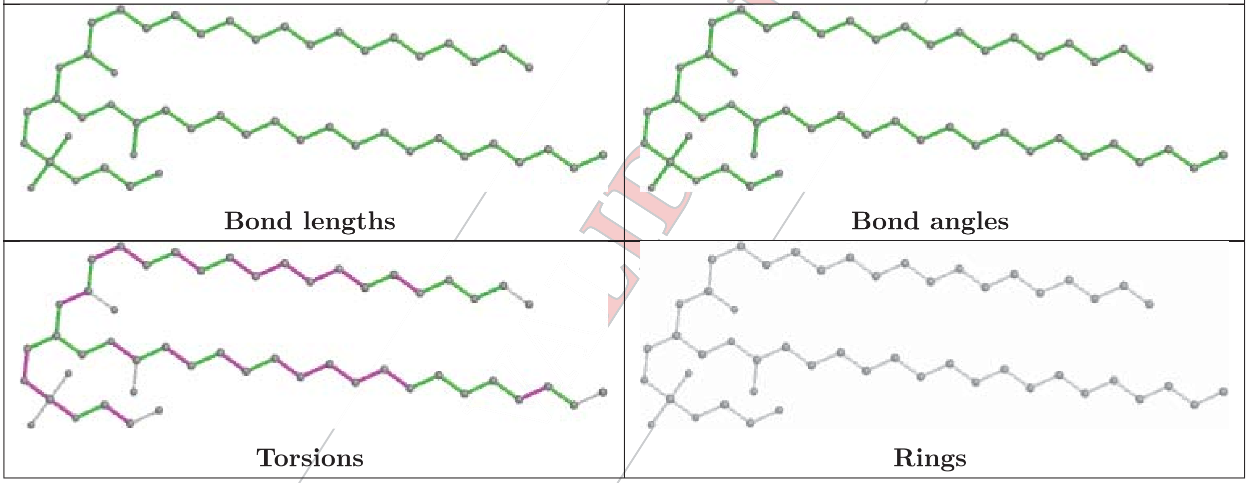

Ligand DLX D 605

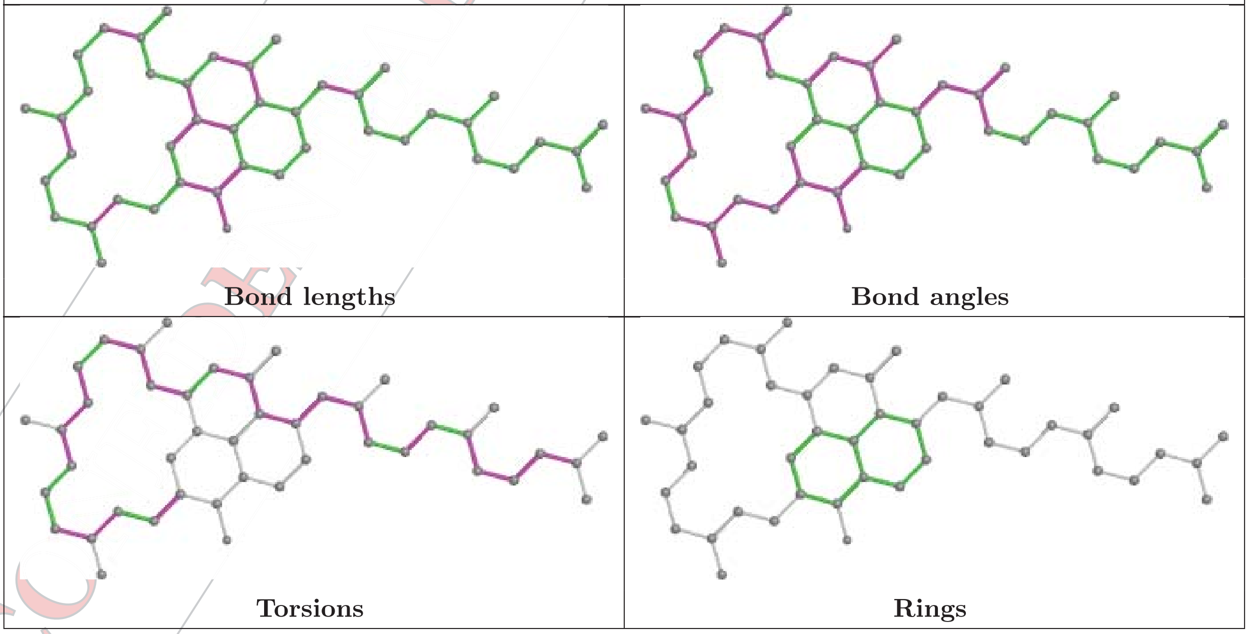

5.7 Other polymers ⓘ

There are no such residues in this entry.

5.8 Polymer linkage issues ⓘ

There are no chain breaks in this entry.

CONFIDENTIAL VALIDATION REPORT

6 Map visualisation (i)

This section contains visualisations of the EMDB entry EMD-30657. These are intended to permit visual inspection of the internal detail of the map and identification of artifacts.

6.1 Orthogonal projections (i)

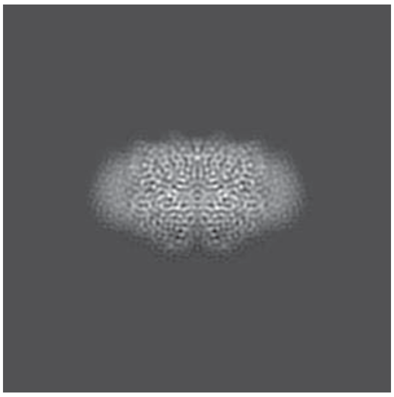

X

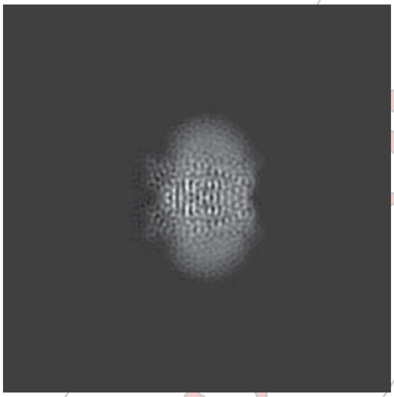

Y

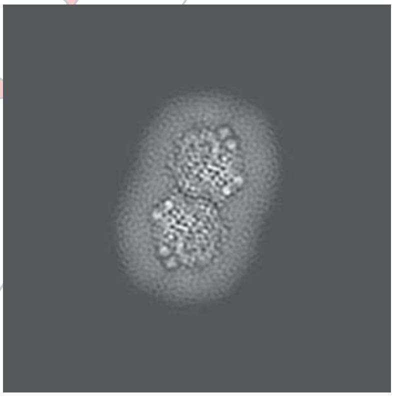

Z

The images above show the map projected in three orthogonal projections, in greyscale.

6.2 Central slices (i)

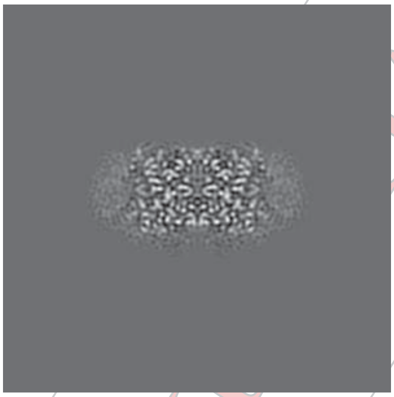

X

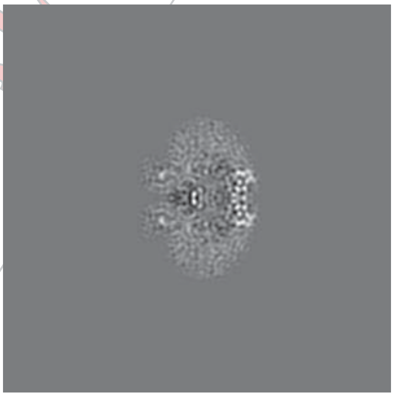

Y

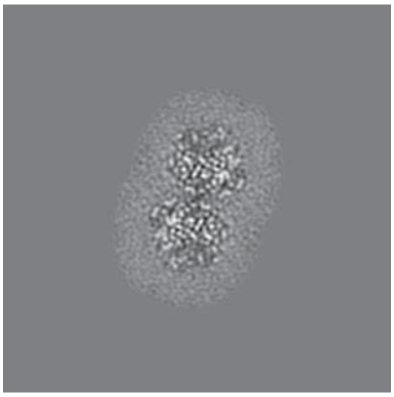

Z

The images above show central slices of the map in three orthogonal directions, in greyscale.

CONFIDENTIAL VALIDATION REPORT

6.3 Largest variance slices (i)

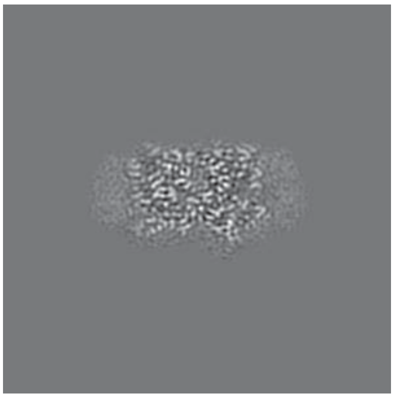

X Index: 131

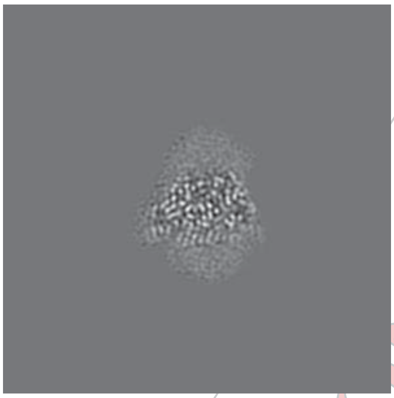

Y Index: 115

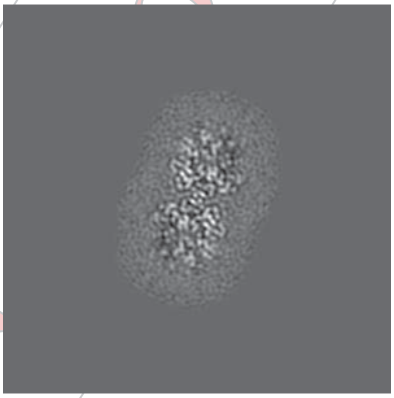

Z Index: 121

The images above show the highest variance slices of the map in three orthogonal directions, in greyscale.

6.4 Orthogonal surface views (i)

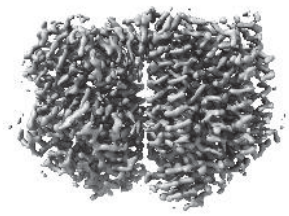

X

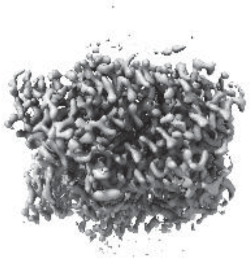

Y

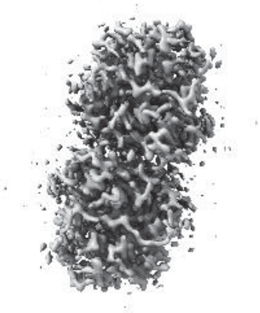

Z

The images above show the 3D surface view of the map at the recommended contour level 0.02. This in conjunction with the slice images can indicate whether an appropriate contour level has been selected.

6.5 Mask visualisation (i)

This section was not generated. No masks were provided.

CONFIDENTIAL VALIDATION REPORT

7 Map analysis ⓘ

This section contains the results of statistical analysis of the map.

7.1 Map-value distribution ⓘ

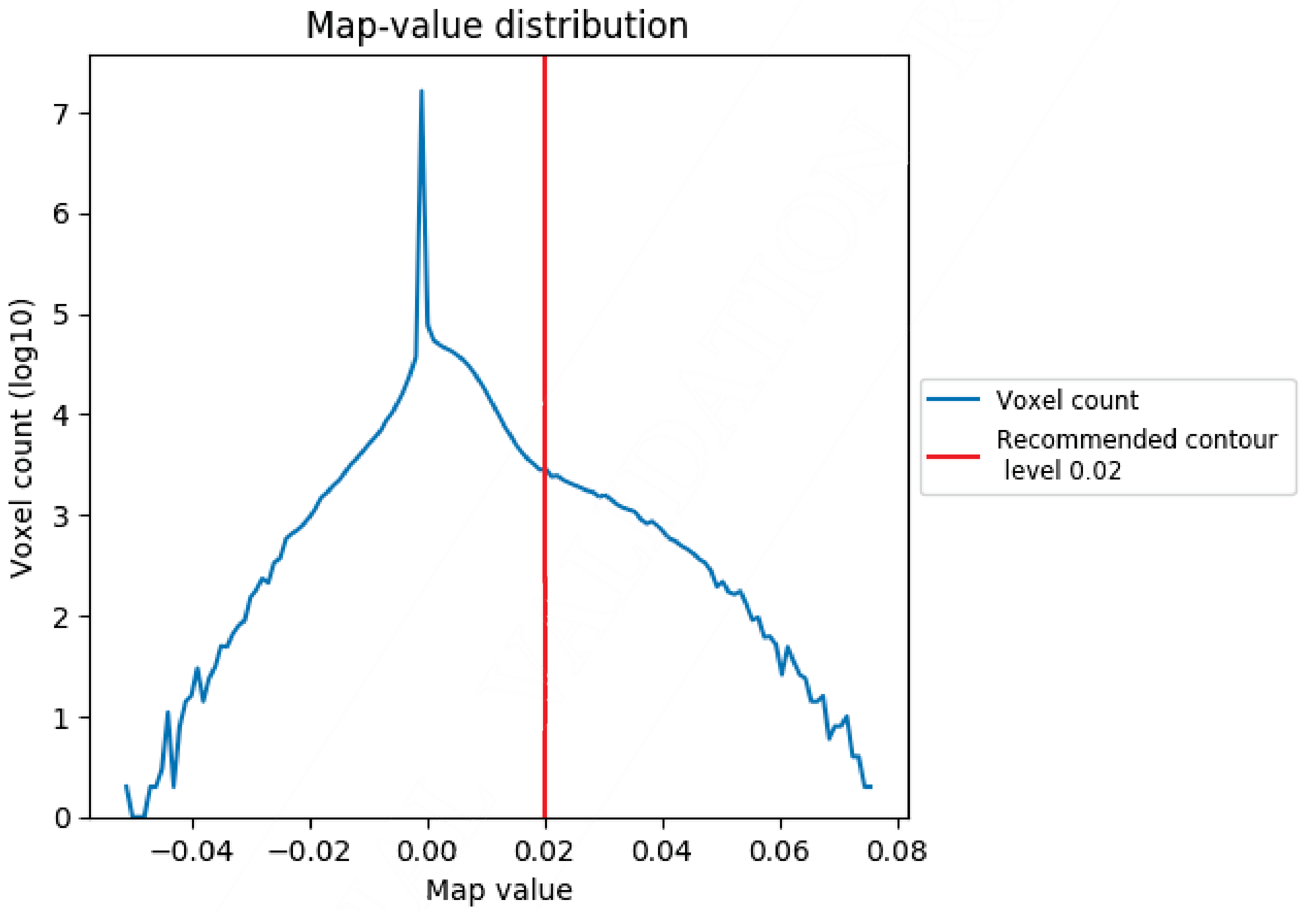

The map-value distribution is plotted in 128 intervals along the x-axis. The y-axis is logarithmic. A spike in this graph at zero usually indicates that the volume has been masked.

CONFIDENTIAL VALIDATION REPORT

7.2 Volume estimate (i)

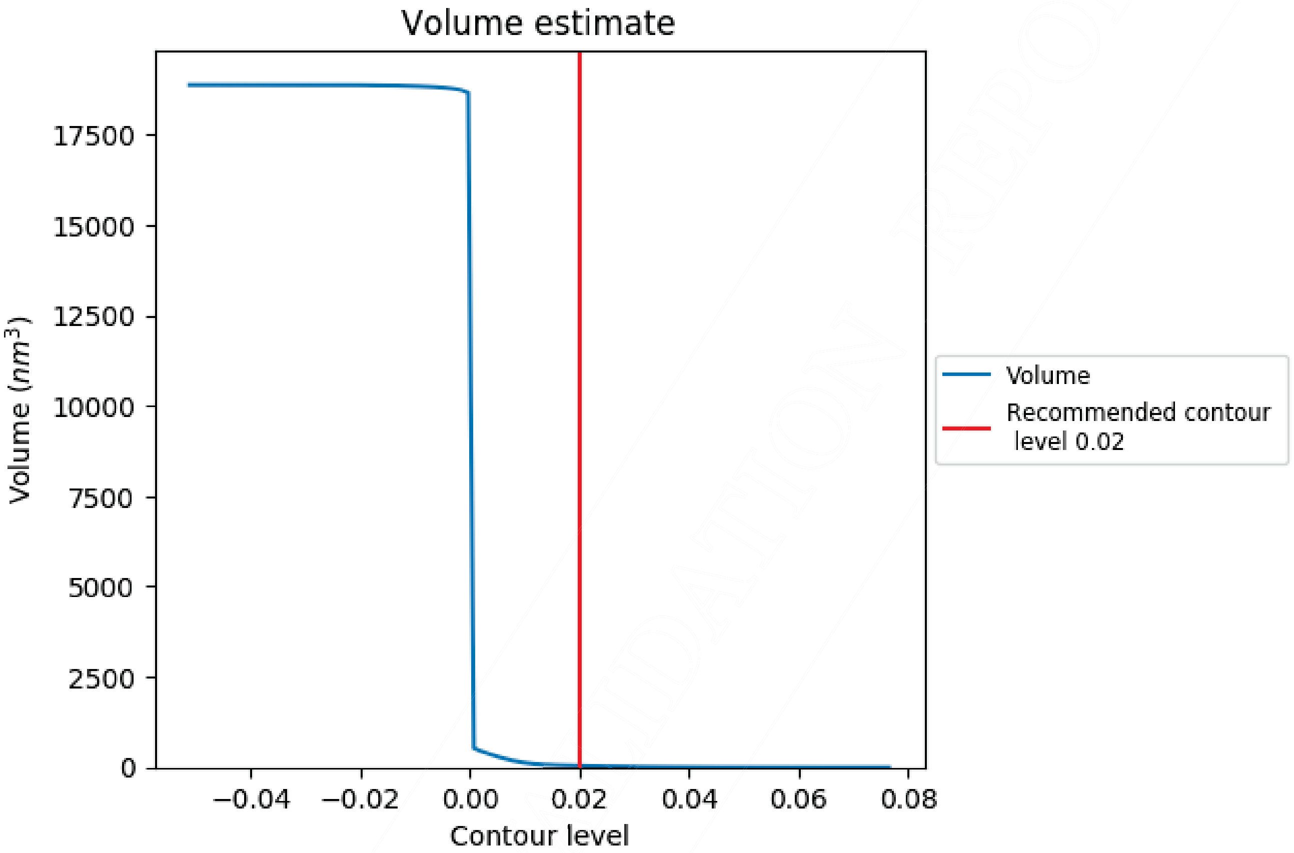

The volume at the recommended contour level is 43 nm^3; this corresponds to an approximate mass of 39 kDa.

The volume estimate graph shows how the enclosed volume varies with the contour level. The recommended contour level is shown as a vertical line and the intersection between the line and the curve gives the volume of the enclosed surface at the given level.

CONFIDENTIAL VALIDATION REPORT

7.3 Rotationally averaged power spectrum ⓘ

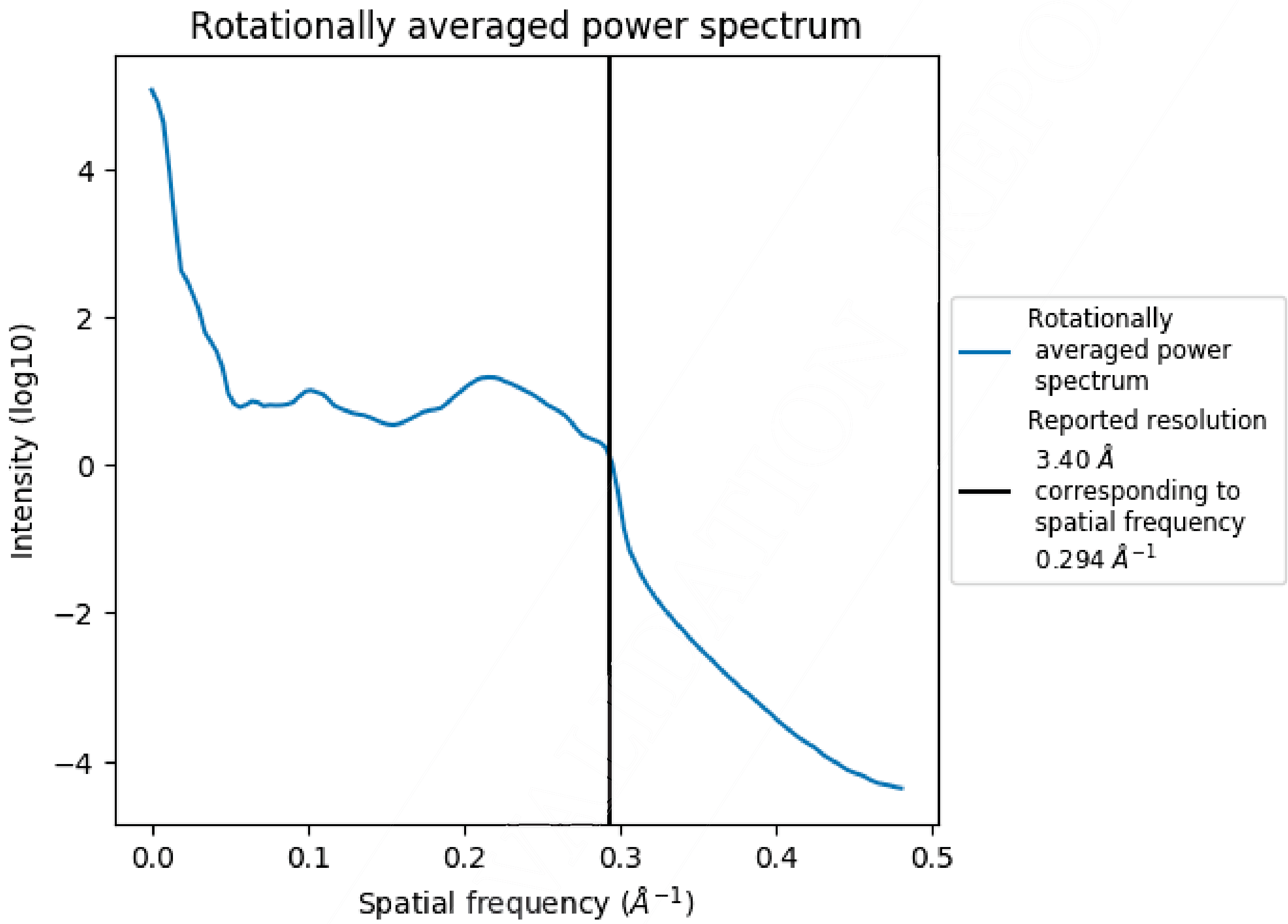

CONFIDENTIAL VALIDATION REPORT

8 Fourier-Shell correlation (i)

Fourier-Shell Correlation (FSC) is the most commonly used method to estimate the resolution for single-particle and subtomogram-averaging methods. The shape of the curve depends on the imposed symmetry, mask and whether or not the two 3D reconstructions used were processed from a common reference. The reported resolution is shown as a black line. Curves are displayed for 3σ, 1-bit and 1/2-bit in addition to lines showing the 0.143 gold standard cut-off, 0.333 cut-off and legacy 0.5 cut-off.

8.1 Resolution estimates (i)

These are global values for the map.

Source	Criterion	Resolution estimate (Å)
Reported value	FSC 0.143 CUT-OFF	3.40
Author-provided FSC	FSC 0.5 CUT-OFF	3.97
Author-provided FSC	FSC 1 BIT CUT-OFF	3.77
Author-provided FSC	FSC 0.33 CUT-OFF	3.76
Author-provided FSC	FSC 1/2 BIT CUT-OFF	3.49
Author-provided FSC	FSC 0.143 CUT-OFF	3.45
Author-provided FSC	FSC 3 SIGMA CUT-OFF	3.03

8.2 Calculated FSC (i)

This section was not generated. Half-maps were not provided.

CONFIDENTIAL VALIDATION REPORT

8.3 Author-provided FSC (i)

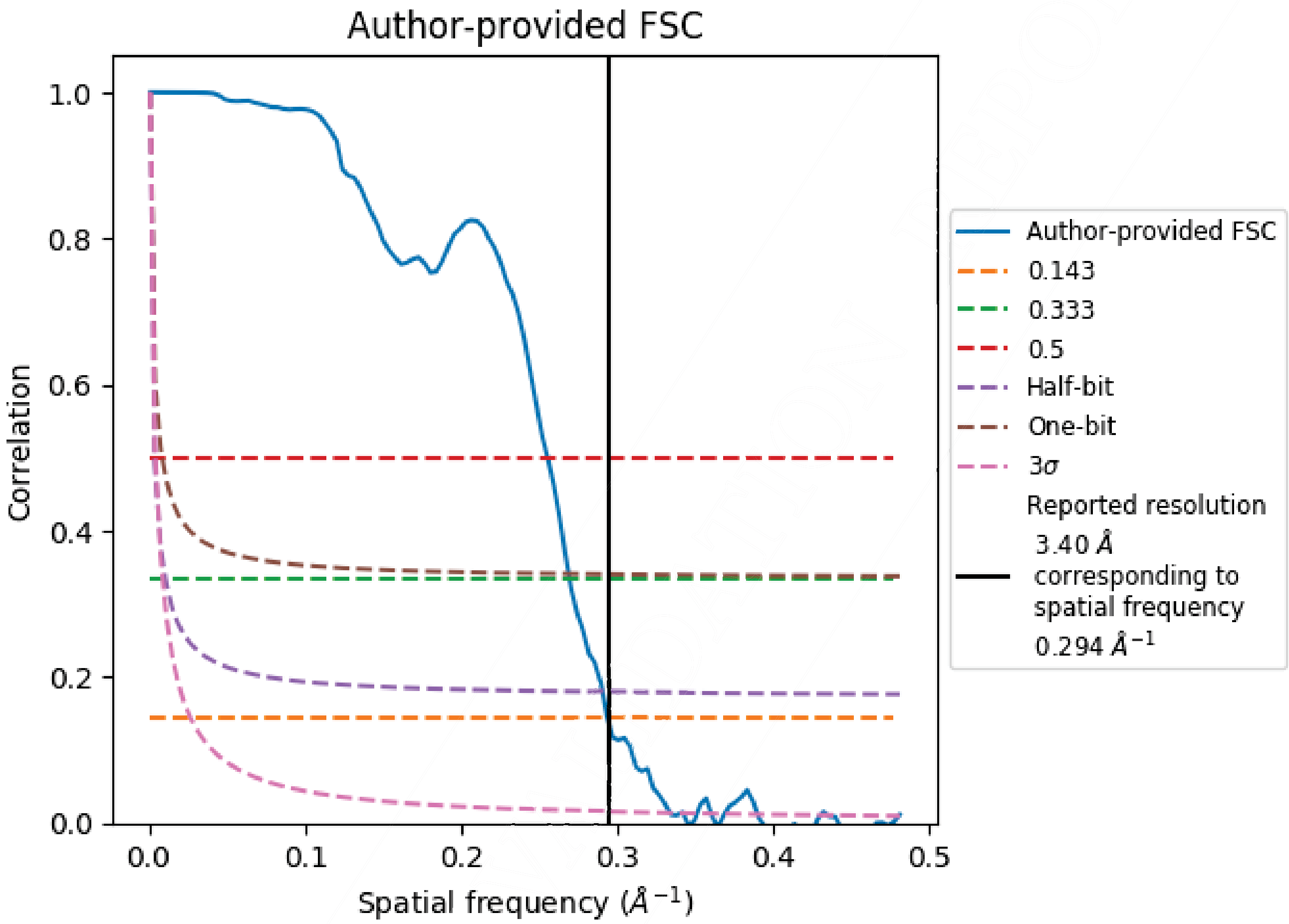

This FSC information was provided by the depositor.

CONFIDENTIAL REPORT

9 Map-model fit (i)

This section contains information regarding the fit between EMDB map EMD-30657 and PDB model 7DEG. Per-residue inclusion information can be found in section 3 on page 9.

9.1 Map-model overlay (i)

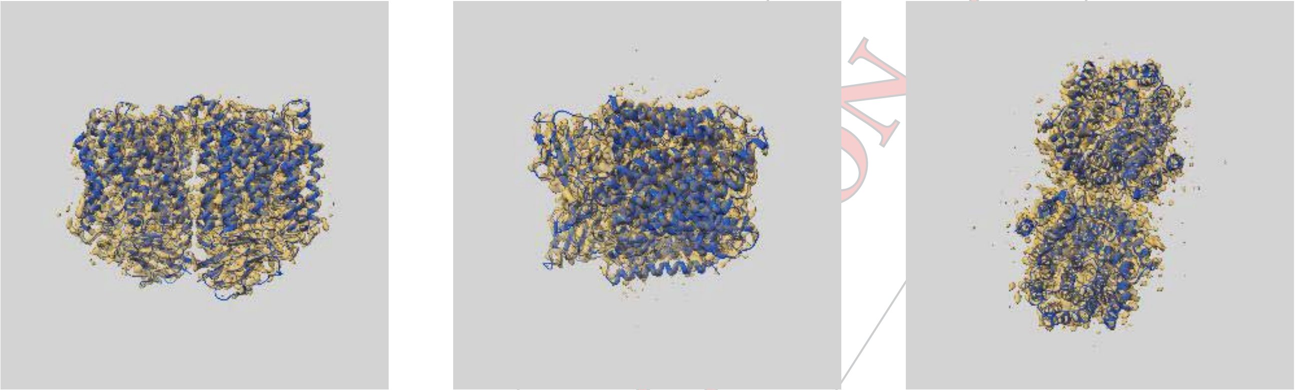

X Y Z

The images above show the 3D surface view of the map at the recommended contour level 0.02 at 50% transparency in yellow overlaid with a ribbon representation of the model coloured in blue. These images allow for the visual assessment of the quality of fit between the atomic model and the map.

CONFIDENTIAL VALIDATION REPORT

9.2 Atom inclusion (i)

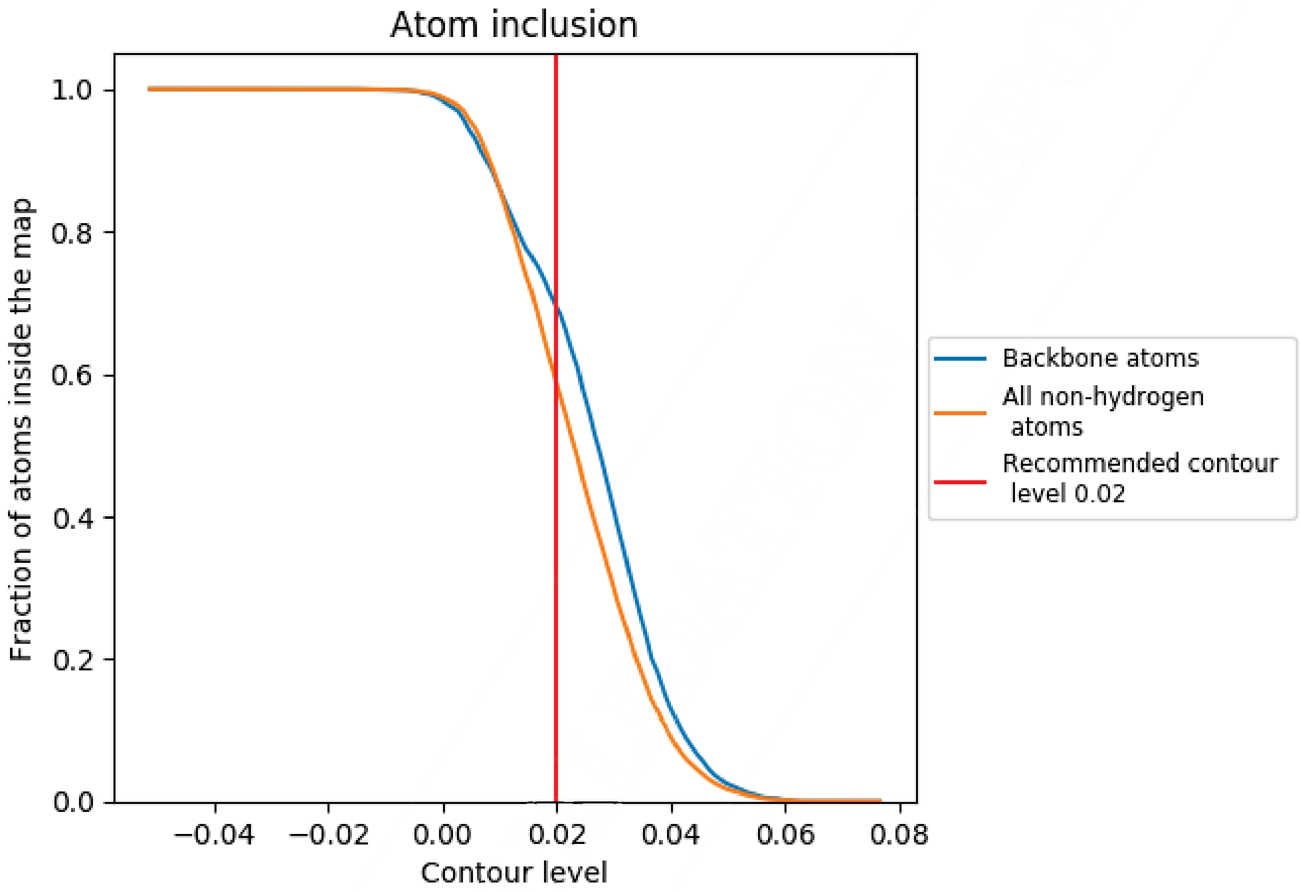

At the recommended contour level, 69% of all backbone atoms, 59% of all non-hydrogen atoms, are inside the map.

CONFIDENTIAL VALIDATION REPORT
